# The mitochondrial genome of the mountain wooly tapir, *Tapirus pinchaque* and a formal test of the effect of altitude on the adaptive evolution of mitochondrial protein coding genes in odd-toed ungulates

**DOI:** 10.1186/s12864-023-09596-8

**Published:** 2023-09-06

**Authors:** Edgar G. Gutiérrez, Jorge Ortega, Avery Savoie, J. Antonio Baeza

**Affiliations:** 1 Laboratorio de Bioconservación y Manejo, Posgrado en Ciencias Químicobiológicas, Departamento de Zoología, Escuela Nacional de Ciencias Biológicas, Instituto Politécnico Nacional, Prolongación Carpio y Plan de Ayala S/N, Col. Santo Tomás, Ciudad de México, C.P. 11340 Mexico; 2 Department of Biological Sciences, 132 Long Hall, Clemson University, Clemson, SC 29634 USA; 3 Smithsonian Marine Station at Fort Pierce, 701 Seaway Drive, Fort Pierce, FL 34949 USA; 4 Departamento de Biología Marina, Facultad de Ciencias del Mar, Universidad Católica del Norte, Larrondo 1281, Coquimbo, Chile

**Keywords:** Adaptive evolution, Mitochondrial genes, Odd-toed ungulates, Selection, Selective pressure, *Tapirus pinchaque*

## Abstract

**Background:**

The harsh conditions of high-altitude environments are known to drive the evolution of physiological and morphological traits in endothermic animals. These conditions are expected to result in the adaptive evolution of protein coding genes encoded in mitochondrial genomes that are vital for the oxidative phosphorylation pathway. In this study, we formally tested for signatures of adaptive evolution on mitochondrial protein coding genes in *Tapirus pinchaque* and other odd-toed ungulates inhabiting high-elevation environments.

**Results:**

The AT-rich mitochondrial genome of *T. pinchaque* is 16,750 bp long. A phylomitogenomic analysis supports the monophyly of the genus *Tapirus* and families in the Perissodactyla. The ratio of non-synonymous to synonymous substitutions demonstrated that all mitochondrial genes undergo purifying selection in *T. pinchaque* and other odd ungulates living at high elevations. Over this negative background selection, Branch Models suggested that *cox3* and *nad6* might be undergoing stronger purifying selection than other mitochondrial protein coding genes. Furthermore, Site Models suggested that one and four sites in *nad2* and *nad5*, respectively, could be experiencing positive selection. However, these results were supported by Likelihood Ratio Tests but not Bayesian Empirical Bayes posterior probabilities. Additional analyses (in DataMonkey) indicated a relaxation of selection strength in *nad6*, evidence of episodic diversifying selection in *cob*, and revealed episodic positive/diversifying selection signatures for two sites in *nad1*, and one site each in *nad2* and *nad4*.

**Conclusion:**

The mitochondrial genome of *T. pinchaque* is an important genomic resource for conservation of this species and this study contributes to the understanding of adaptive evolution of mitochondrial protein coding genes in odd-toed ungulates inhabiting high-altitude environments.

**Supplementary Information:**

The online version contains supplementary material available at 10.1186/s12864-023-09596-8.

## Background

During their evolutionary radiation, mammals have colonized a wide variety of environments, including high-altitude habitats characterized by extreme conditions, such as low atmospheric pressure, reduced oxygen (including hypoxia), high solar radiation, and low temperatures [1, 2]. Consequently, high-altitude mammals exhibit a set of specialized morphological and physiological traits that have been considered adaptations to the harsh conditions characteristic of these environments [3]. Typical convergent putative adaptations in high-altitude organisms include, among others, small body sizes (relative to their closely related species) that decrease energy demand, and dense long coats to minimize heat dissipation (e. g., in some sheep breeds) [3–5]. Physiological strategies to cope with hypoxic conditions at high altitudes include hypoxia-tolerance coupled with low metabolic rates to reduce oxygen demand [6, 7] and high hemoglobin concentrations plus increased hemoglobin binding-affinity for oxygen, as reported in highland mammals such as the yak [8], the alpaca [9], and the deer mouse [10], among others [11]. Most recently, specialized endothelial cells in the lungs of yaks have been discovered that might help individuals to thrive in the cold, low-oxygen conditions characteristic of high-altitude environments [12].

The genomic underpinnings of adaptation to high altitude environments are not well understood but have been extensively studied during the last decade (in the yak; *Bos grunniens*, the Tibetan gray wolf; *Canis lupus chanco*, and sheep; *Ovis aries*) [12–15]. For instance, in the nuclear genome, candidate genes that have contributed to adaptation to high altitudes include EPAS1 (endothelial PAS domain protein 1; also known as HIF-2A) and EGLN1 (egl-9 family hypoxia inducible factor 1; also known as HIF-prolylhydroxylase 2). These two genes are involved in the response to hypoxic stress [14, 15] and have been shown to experience strong signatures of positive selection [7, 11, 14, 16]. The EPAS1 gene directly regulates key genes such as erythropoietin and vascular endothelial growth factor, while the EGLN1 gene negatively regulates the activity of hypoxia-inducible factor-1 alpha (HIF-1A), a transcriptional complex that plays a central role in oxygen homeostasis in mammals [14, 17]. Adaptive evolution in mitochondrial genome (mtDNA) of mammals inhabiting high-altitude environments has been explored in a few species (Tibetan horses [18], alpacas [19], and camelids [20], among a few others).

Mitochondria are responsible for supplying cellular energy to the cells in the form of adenosine triphosphate (ATP) through the oxidative phosphorylation system (OXPHOS) carried out by the electron transport chain and ATP synthase [21]. Furthermore, ATP is used for generating heat and maintaining body temperature in endothermic animals, including mammals [1]. Importantly, hypoxic conditions, characteristic of high-altitude environments, are known to affect physiological processes involving metabolic performance and increases in Reactive Oxygen Species (ROS) [22]. ROS produces superoxide (O_2_.^−^) and hydrogen peroxide (H_2_O_2_) that triggers oxidative damage to lipids, proteins, and deoxyribonucleic acid (DNA) [23, 24]. Similarly, hypoxia inhibits the transcription of mitochondrial DNA and damages the structure and function of mitochondria. Consequently, mitochondrial protein-coding genes are expected to experience selective pressures in species that are continuously exposed to harsh environmental conditions [2, 25, 26]

The mammalian mtDNA is a circular, double-stranded molecule that hosts its own genetic material [23, 27]. Invariably mammalian mtDNA contains 22 transfer RNA genes (tRNA), two ribosomal genes (rRNA), and 13 protein coding genes (PCG´s). The latter genes encode polypeptides that have a key role in the OXPHOS [2, 19]. Specifically, these OXPHOS-related polypeptides (7 subunits of the NADH dehydrogenase complex, 3 subunits of the cytochrome c oxidase, 2 subunits of ATP synthase, and the cytochrome b subunit of the cytochrome bc1 complex) are involved in electron transfer and aerobic respiration [22]. Changes in mitochondrial function driven by mutations in genes involved in oxidative phosphorylation, are likely essential for adaptation to adverse conditions (including hypoxia) typical of high-altitude environments [23].

The order Perissodactyla comprises the odd-toed ungulates and contains three families: Equidae, Rhinocerotidae, and Tapiridae [28]. The family Tapiridae comprises a single extant genus, *Tapirus*, represented by four extant species [29]; two exclusively distributed in South America (*Tapirus terrestris* and *Tapirus pinchaque*); *T. bairdii* which is distributed in Southern Mexico, Central America, and the Pacific coast of Colombia, and *Tapirus indicus*, that inhabits Southern Thailand and through peninsular Malaysia to Indonesia [30–32]. A fifth species of *Tapirus* was proposed nearly a decade ago [33]; however, analysis using mitochondrial markers (i. e., *cox1, cox2* and *cob* genes) demonstrated that haplotypes from individuals identified as *T. kabomani* clustered together with haplotypes from individuals identified as *T. terrestris*, and haplotypes did not segregate according to putative species [34]. Thus, *T. kabomani* is currently considered a synonym of *T. terrestris*.

**Fig. 1:**
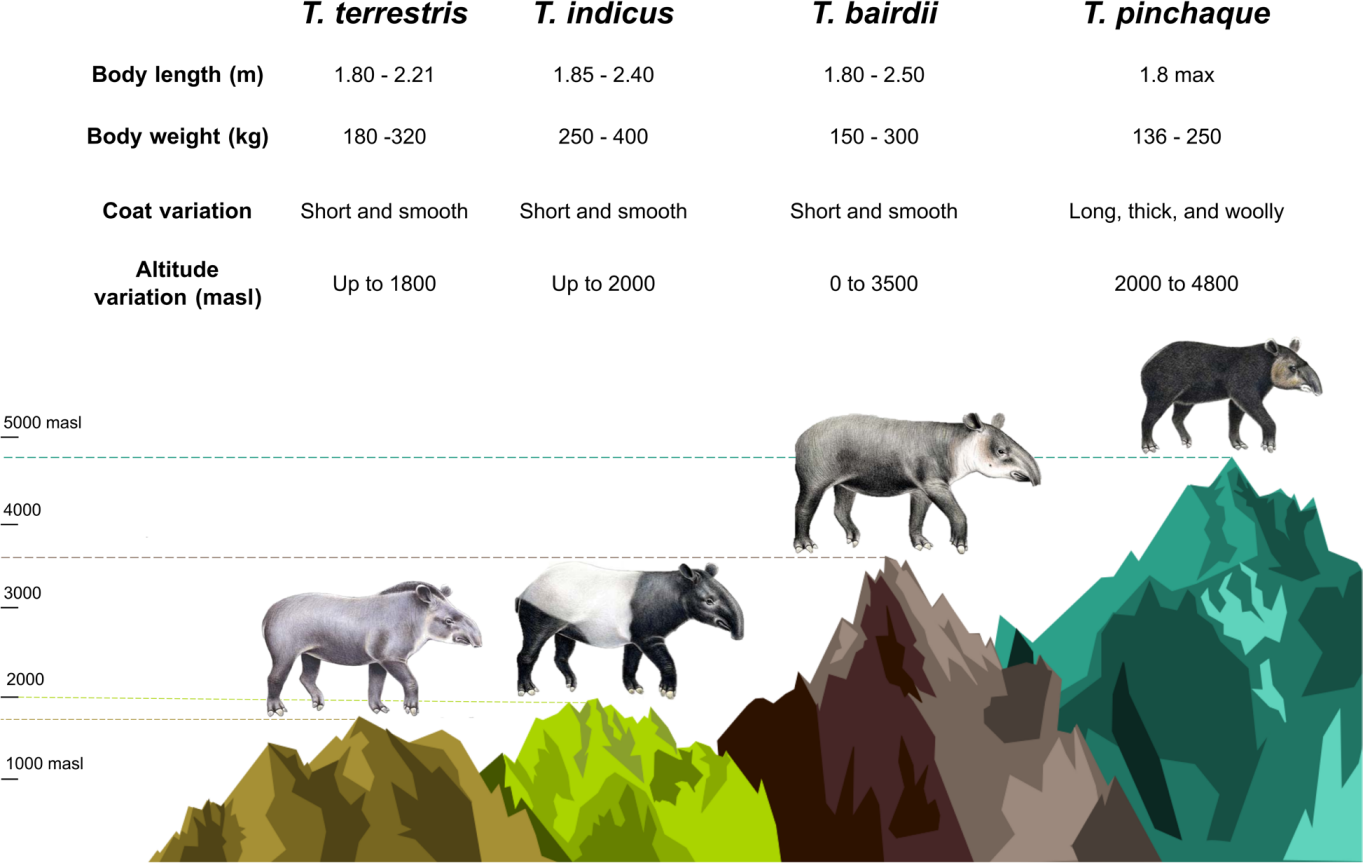
Ecological and phenotypic characteristics of *Tapirus pinchaque* and congeneric species. Tapir species ilustration credit: Stephen Nash (published with permission)

Among tapirs, the mountain or wooly tapir, *Tapirus pinchaque* inhabits the tropical montane Andean forests and ‘paramos’ between 2,000 and 4,800 m above sea level (masl) in Colombia, Ecuador, and northern Peru [35, 36] (Fig. 1). By contrast to *T. pinchaque*, all other congeneric species primarily prefer relatively lower altitude habitats, e. g., *Tapirus terrestris* inhabits lowland forests up to 1,800 masl [37, 38] while *T. indicus* can be found up to 2,000 masl, with a greater presence in lower altitude forests (125 to 1000 m) [39]. Lastly, the distribution of *T. bairdii* is limited to the states of Campeche, Chiapas, Oaxaca, Quintana Roo, and Veracruz in Mexico, from sea level to altitudes not higher than 2000 masl [40]. However, much of the literature describes the distribution in Central America from sea level up to 3600 m [41–43]. Other than a white line around its lips, *T. pinchaque* is distinguished from other relatives by a thick wooly coat (composed of 2–4 cm long hair) and the presence of a thick inner layer of fat [35, 44] that might well be interpreted as adaptations to high altitude. T*apirus pinchaque* is also the smallest species (1.8 m maximum length) [45] in the genus (*T. terrestris*, *T. bairdii*, and *T. indicus* range in body length between 1.8 to 2.5 m in adult individuals) [37, 40, 46] (Fig. 1). The smaller size of *T. pinchaque* is probably related to lower energy expenditure, which has been suggested to be another adaptive phenotypic trait for living at high altitudes [4]. Furthermore, mountain tapirs often swim and wallow in water bodies, a behavior likely used to cool their bodies due to high radiation during the day at high altitude [47]. The genomic basis of such adaptations in *T. pinchaque*, however, remains unknown. We focus on the mtDNA and we are particularly interested in studying the effect of altitude on the adaptive evolution of protein coding genes in the mountain tapir and odd-toed ungulates.

This study aims to explore adaptive evolution in the mtDNA of *T. pinchaque* and other species belonging to the order Perissodactyla that inhabit high-altitude environments. To achieve the aforementioned goal, first, we assembled and characterized in detail the complete mitochondrial genome of the mountain tapir *Tapirus pinchaque* profiting from low-pass (= low coverage) next generation short-read sequencing. We describe the mitochondrial genome of this species in detail following suggestions in Baeza [48]. Specifically, the nucleotide composition of the entire mtDNA, the codon usage of PCG´s, the secondary structure of each tRNA, the secondary structure and tandem repeats within the putative control region (CR) were calculated, and then compared against other congeneric extant species. After an analysis of mitogenomic features in the genus *Tapirus*, we explored the phylogenetic position of *T. pinchaque* among extant congeneric species and generated a phylogenetic hypothesis for the Perissodactyla based on mitochondrial protein-coding genes. Lastly, we used a set of bioinformatic tools to estimate the ratio ω of non-synonymous (d_N_) to synonymous (d_S_) substitutions rate (ω = d_N_/d_S_) in order to identify signatures of adaptive evolution (i. e., positive selection) on PCG´s in members of the Perissodactyla adapted to live in high altitude environments. High-altitude species, including *T. pinchaque* were compared against species living below 2,000 masl, using Branch Models (BM), Site Models (SM), and Branch-Site Models (BSM), along sequences or across branches of a phylogeny [49, 50]. We predict that the protein coding genes in the mitocondrial genome of *T. pinchaque* and other species belonging to the order Perissodactyla that inhabit high-altitude environments exhibit signatures of positive selection compared to closely related species inhabiting low-altitude environments. The genomic resource generated in this study and analyses provide new insights into the molecular mechanisms responsible for adaptation to high-altitude environments in *T. pinchaque* and other representatives of Perissodactyla.

Importantly, other than its peculiar high-altitude lifestyle, *T. pinchaque* is considered an important member of its community: more than 50 species of seeds have been found in feces from individuals inhabiting the Ecuadorian Andes highlighting the putative role of this large mammal in seed dispersal [34, 51]. Despite the potentially crucial ecological role of this species, there is a very small number of genetic studies for *T. pinchaque* [34]. Thus, the assembly and detailed description of its mitochondrial genome coupled with the formal test of adaptive evolution in mitochondrial protein coding genes in this and other odd-toed ungulates represents a step forward to improve genomic resources that can aid in the conservation of this species currently experiencing major environmental issues [52, 53].

## Results and discussion

**Fig. 2:**
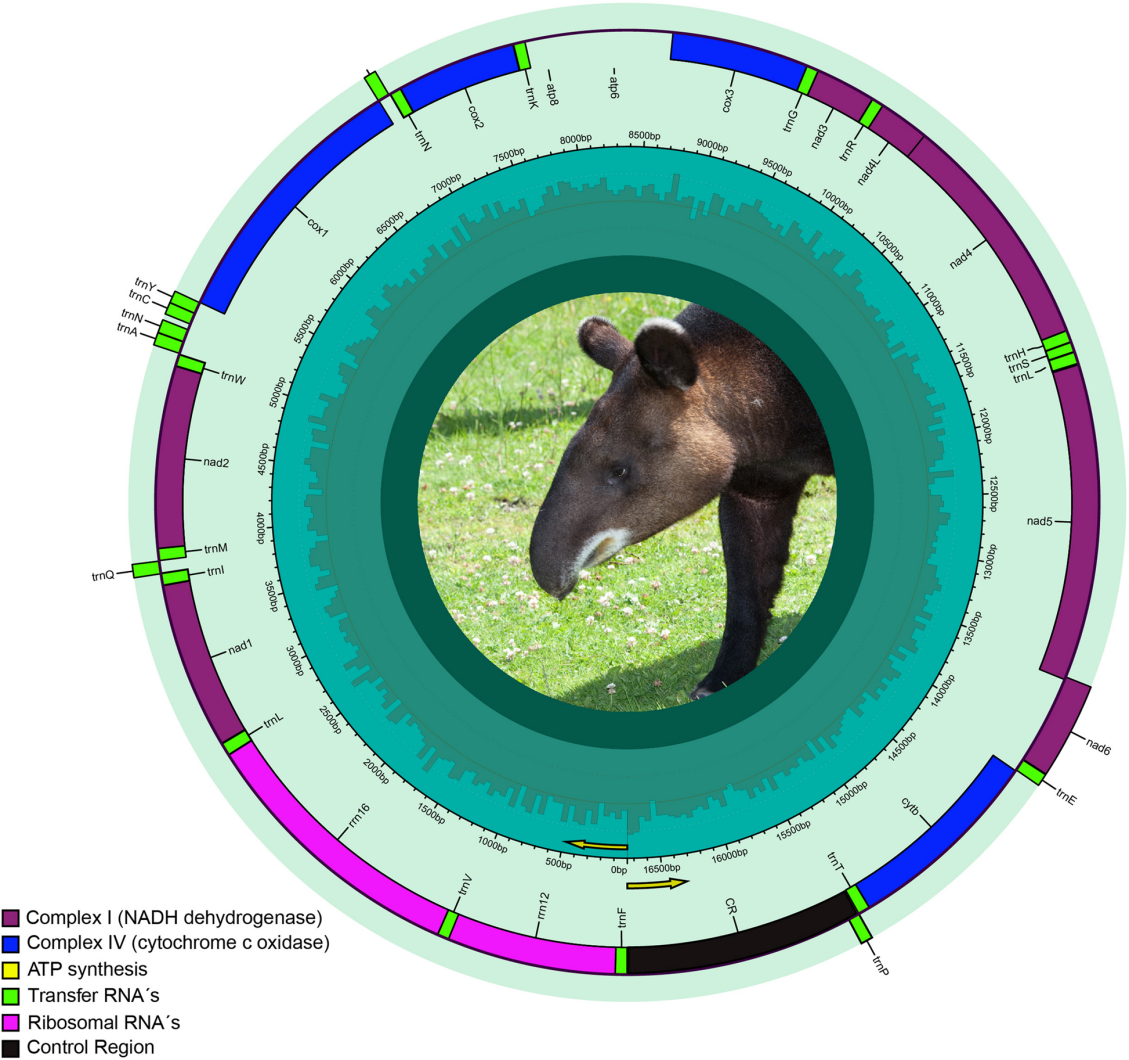
Circular representation of the complete mitochondrial genome in *Tapirus pinchaque.* The 13 PCG´s, 22 tRNA's, 2 rRNA's, and the control region are annotated. Photo credit: David Sifry (published with permission)

The pipeline GetOrganelle [54] assembled (with an average coverage of 169.5 × per nucleotide) and circularized the complete mitochondrial chromosome (GenBank accession no. OQ420428) of *T. pinchaque* (Fig. 2). The complete sequence of *T. pinchaque* mtDNA is 16,750 bp long, and exhibits a length similar to that found in *T. terrestris* (16,772 bp) [55], *T. bairdii* (16,697 bp) [56], *T. indicus* (16,717 bp) [57], as well as in other members of the order Perissodactyla (e. g., Rhinocerotidae: *Ceratotherium simum* – 16,832 bp and *Rhinoceros unicornis* – 16,829 bp; Equidae: *Equus asinus* – 16,813 bp and *Equus caballus* – 16,504 bp) [58–61].

The mitochondrial genome of *T. pinchaque* encodes 13 PCG´s, two rRNA genes (rrnS [12S ribosomal RNA] and rrnL [16S ribosomal RNA]), 22 tRNAs, and exhibits a relatively long (1,302 bp) non-coding putative CR or D-Loop (Table 1). In the mtDNA of *T. pinchaque*, all PCG´s are encoded in the positive or heavy strand (H), except *nad6* which is located in the negative or light strand (L) (Fig. 2). Equally, the two rRNA’s and 14 tRNA genes are encoded in the H strand. The remaining eight tRNA genes are located in the L strand (Fig. 2). The gene order herein reported for *T. pinchaque* is identical to that previously observed in the congeneric *T. terrestris* [55], *T. bairdii* [56], and *T. indicus* [57]. Likewise, mitochondrial synteny in the genus *Tapirus* is identical to that reported for members of the closely related families Equidae (e. g., Tibetan wild ass; *Equus kiang* and Qingyang donkey; *Equus asinus*) [62, 63] and Rhinocerotidae (e. g., *Rhinoceros sondaicus* and *Diceros bicornis*) [64] with complete mitochondrial genomes available in GenBank.

**Table 1:** Mitochondrial genome of *Tapirus pinchaque*. Arrangement and annotation. * = incomplete stop codon

Name	Type	Start	Stop	Strand	Length (bp)	Start	Stop	Continuity
***trnF (ttc)***	tRNA	1	68	+				0
***rrnS***	rRNA	69	1037	+				-1
***trnV(gta)***	tRNA	1037	1103	+				-2
***rrnL***	rRNA	1102	2682	+				0
***trnL2(tta)***	tRNA	2683	2757	+				2
***nad1***	PCG	2760	3716	+	957	ATG	*TAA*	0
***trnI(atc)***	tRNA	3716	3785	+				-3
***trnQ(caa)***	tRNA	3783	3855	-				2
***trnM(atg)***	tRNA	3858	3926	+				0
***nad2***	PCG	3927	4968	+	1042	ATA	Taa*	0
***trnW(tga)***	tRNA	4969	5037	+				5
***trnA(gca)***	tRNA	5043	5111	-				1
***trnN(aac)***	tRNA	5113	5185	-				32
***trnC(tgc)***	tRNA	5219	5284	-				0
***trnY(tac)***	tRNA	5285	5351	-				1
***cox1***	PCG	5353	6897	+	1545	ATG	*TAA*	-3
***trnS2(tca)***	tRNA	6895	6963	-				7
***trnD(gac)***	tRNA	6971	7037	+				0
***cox2***	PCG	7038	7721	+	684	ATG	*TAA*	3
***trnK(aaa)***	tRNA	7725	7791	+				1
***atp8***	PCG	7793	7996	+	204	ATG	*TAA*	-43
***atp6***	PCG	7954	8634	+	681	ATG	*TAA*	-1
***cox3***	PCG	8634	9417	+	784	ATG	Taa*	0
***trnG(gga)***	tRNA	9418	9486	+				0
***nad3***	PCG	9487	9832	+	346	ATA	Taa*	1
***trnR(cga)***	tRNA	9834	9901	+				0
***nad4l***	PCG	9902	10,198	+	297	ATG	*TAA*	-7
***nad4***	PCG	10,192	11,569	+	1378	ATG	Taa*	0
***trnH(cac)***	tRNA	11,570	11,638	+				0
***trnS1(agc)***	tRNA	11,639	11,697	+				0
***trnL1(cta)***	tRNA	11,698	11,767	+				6
***nad5***	coding	11,774	13,588	+	1815	ATA	*TAA*	-17
***nad6***	coding	13,572	14,096	-	525	ATG	*TAA*	3
***trnE(gaa)***	tRNA	14,100	14,168	-				5
***cob***	coding	14,174	15,313	+	1140	ATG	*AGA*	0
***trnT(aca)***	tRNA	15,314	15,382	+				0
***trnP(cca)***	tRNA	15,383	15,448	-				
***CR***		15,449	16,750	+				

Nucleotide usage in the complete mitochondrial sequence of *T. pinchaque* was as follows: T = 27.98%, C = 25.25%, A = 34.36%, G = 12.41%. The G + C content was 37.66%, while the A + T content was 62.3%. The aforementioned A + T content value is similar to those previously reported in the genus *Tapirus* (i. e., *T. terrestris*; 62.22%, and *T. bairdii*; 62.00%; Table 2). The A + T content in *T. indicus* (NC023838) has not been reported [57]. Herein, we estimated nucleotide usage for *T. indicus*, that exhibits the lowest AT-content (61.9%) compared to the rest of the congeneric species. A somewhat lower AT-content has been reported in representatives of the sister clade Rhinocetoridae: *Rhinoceros unicornis* (59.8%) [59], *Rhinoceros sondaicus* (59.8%) [64], and *Dicerorhinus sumatrensis* (58.4%) [65]. The AT-rich nucleotide usage in *Tapirus* and allies is often assumed to be driven by the asymmetric nature of the replication process in mtDNA [66]. Furthermore, AT-content might be influenced by differential mutation pressure at synonymous and non-synonymous sites in PCG´s [67].

**Table 2:** Nucleotide usage values in different Perissodactyla species, including the *Tapirus* species analyzed in this study

*Species*	T(U)%	C%	A%	G%	Length (bp)
***Tapirus pinchaque***	27.9	25.3	34.4	12.4	16,750
***Tapirus indicus***	27.2	25.8	34.6	12.3	16,717
***Tapirus bairdii***	29.8	25.6	32.2	12.3	16,697
***Tapirus terrestris***	27.8	25.4	34.4	12.4	16,766
***Rhinoceros unicornis***	26.2	27.5	33.7	12.7	16,829
***Dicerorhinus sumatrensis***	25.3	28.3	33.1	13.3	16,466
***Diceros bicornis***	25.9	27.9	33.5	12.6	16,594
***Rhinoceros sondaicus***	26.3	27.4	33.5	12.8	16,315

In *Tapirus pinchaque*, 10 PCG´s used ATG as the start codon and the remaining three PCG´s (*nad2*, *nad3*, and *nad5*) used ATA as start codons. Eight of the PCG´s used the conventional TAA stop codon and *cob* gene used AGA as stop codon. The remaining PCG´s (*nad2*, *cox3*, *nad3*, and *nad4*) ended with an incomplete stop codon (T). The relative abundance of start and stop codons in PCG´s of *T. pinchaque* agrees with that previously reported for other species of *Tapirus* (i. e., *T. terrestris*, *T. bairdii*, and *T. indicus*) [55–57] as well as for other representatives of the order Perissodactyla [60, 61]. Similarly, incomplete stop codons (T), like those found in four PCG´s (*nad2*, *cox3*, *nad3*, and *nad4*), have been previously observed in *T. terrestris* [55], *T. bairdii* [56], *T. indicus* [57], as well as in *Ceratotherium simum* [58], in *Rhinoceros unicornis* [59], and in other odd-toed ungulates (e. g., *Equus africanus somalies*) [68].

**Fig. 3:**
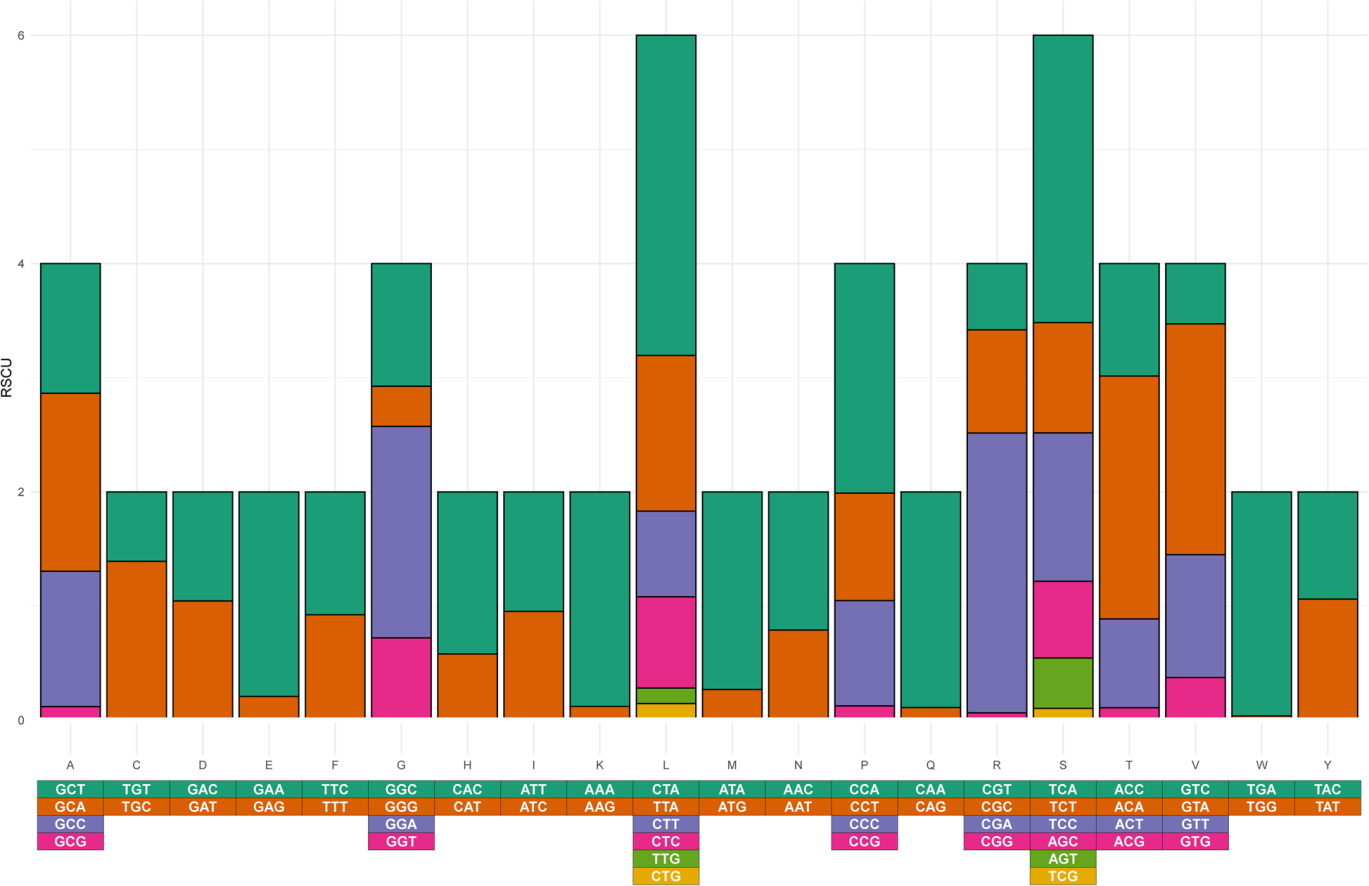
Relative synonymous codon usage (RSCU) analysis in all 13 mitochondrial protein-coding genes of *Tapirus pinchaque*

In the mitochondrial PCG´s of *T. pinchaque*, the eight codons with the highest Relative Synonymous Codon Usage (RSCU) values (= overrepresented codons) were CTA(Leu) (2.77), TCA(Ser) (2.66), CGA(Arg) (2.48), GTA(Val) (2.23), ACA(Thr) (2.10), TGA(Trp) (1.94), CAA(Gln) (1.93), AAA(Lys) (1.92) (Fig. 3, Supplementary Table S1). In turn, underrepresented codons included CGG(Arg) (0.06), TGG(Trp) (0.06), GCG(Ala) (0.07), CAG(Gln) (0.07), and AAG(Lys) (0.08) (Fig. 3, Supplementary Table S1). This is the second time that a detailed analysis of codon usage has been conducted in the family Tapiridae; the first analysis was conducted in *Tapirus bairdii*, and our results are consistent with those reported there [56]. No studies focusing on codon usage of mitochondrial PCG´s were found in other Perissodactyla species, however, the results described above are consistent with studies in other mammals [69, 70]. It should be noted that the RSCU results showed that most of the overrepresented codons (RSCU > 1.9) are A-ending, while underrepresented codons (RSCU < 0.06) are G-ending. The result of the RSCU analysis supports the notion that nucleotide usage is affected by patterns of mutational bias or natural selection, being a driving force of variation in codon usage [71].

**Fig. 4:**
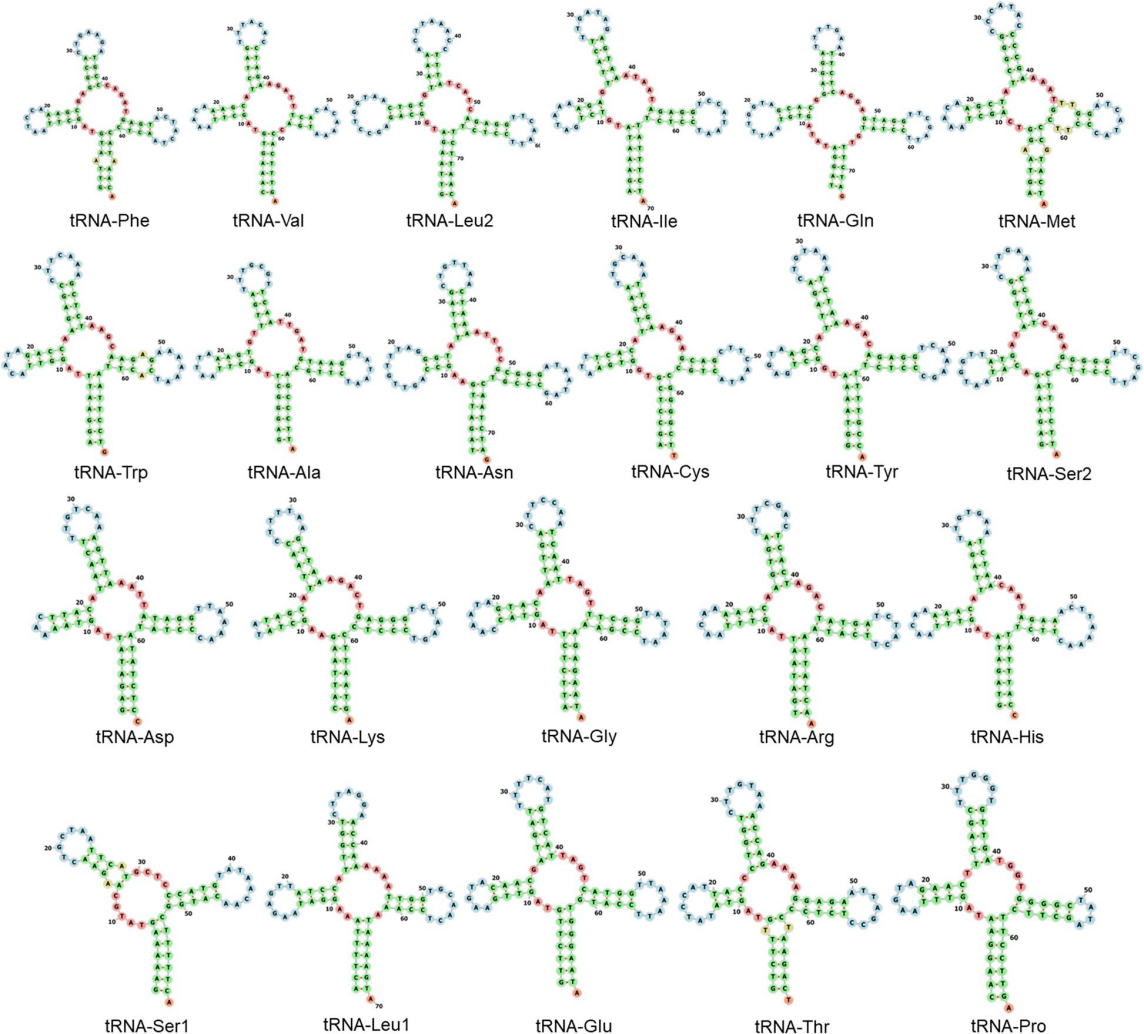
Secondary structure of the 22 tRNA genes present in the mitochondrial genome of *Tapirus pinchaque*

The mtDNA of *T. pinchaque* encoded 22 tRNA genes. The tRNA’s ranged in length from 59 bp (tRNA-Ser1) to 75 bp (tRNA-Leu2). The secondary structures of the tRNA’s detected here (Fig. 4), were very similar to those reported in *T. bairdii* [56]. All tRNA in the *T. pinchaque* mtDNA, except for tRNA-Ser1, exhibited a standard ‘cloverleaf’ secondary structure as indicated by the web server Forna [72]. In *T. pinchaque*, the tRNA-Ser1 has a missing D-arm, and subsequently, it is a relatively short gene (59 bp in length) compared to the other tRNA lengths. Importantly, the loss of stem-loop structures in the D-arm of the tRNA-Ser1 gene appears to be a common feature reported in eumetazoans, including mammals [73]. This is the second study in which the secondary structures of tRNA’s are described in the family Tapiridae.

The *rrnS* (12S) and *rrnL* (16S) genes in the mitochondrial genome of *T. pinchaque* are 969 and 1,579 bp in length, respectively. The 12S gene is located between the *trnF* and *trnV* genes and exhibits a nucleotide content equal to A = 38.6%, T = 23.1%, C = 22.1%, G = 16.2% while the 16S gene is located between *trnV* and *trnL2* and has a nucleotide content equal to A = 38.3%, T = 24.6%, C = 21.5%, G = 15.6%. The two rRNA genes are AT-rich (*rrnS* = 61.7% and *rrnL* = 62.9%). These AT-rich values are consistent with those previously reported for *T. terrestris* (12S = 61.9% and 16S*rrnL* = 62.9%) [55] and *T. bairdii* (12S = 61.7% and 16S = 62.6%) [56]. It should be noted that the nucleotide composition for the 12S gene in *T. indicus* is A = 38.6%, C = 22.6%, T = 22.1%, and G = 16.7% [57]. Therefore, this gene is AC-rich (61.2%), a genomic feature that differs from that reported in other species of *Tapirus*, including *T*. *pinchaque*. The 16S gene of *T. indicus* exhibits an AT-content (63.5%) [57] similar to that reported for other species of *Tapirus*. Interestingly, several Old World odd-toed ungulates exhibit a very similar nucleotide use in both rRNA genes, such as in *Rhinoceros unicornis* (12S = 61.4% A + C, and 16S = 63.1% A + T) [59], *Rhinoceros sondaicus* (12S = 60.9% A + C, and 16S = 62.8% A + T) [64], and in *Dicerorhinus sumatrensis* (12S = 62.0% A + C, and 16S = 61.0% A + T) [65].

The length of the CR in the mitochondrial sequence of *T. pinchaque* is 1,301 bp long and exhibits a nucleotide composition equal to A = 33.8%, T = 28.3%, C = 25.1%, and G = 12.7%. We noted that the length of the control region in *T. pinchaque* is slightly longer than that of *T. bairdii* (1,247 bp) [56] and *T. indicus* (1,268 bp) [57]. The length of this non-coding region varies moderately in the order Perissodactyla and ranges from a minimum length of 1,086 bp in the Turkish Anatolian donkey; *Equus asinus* [74] to a maximum of 1,376 bp in the Indian rhinoceros; *R*. *unicornis* [59]. The length of the CR is known to vary extensively among mammals [75], and the differences in length are likely driven by the fast mutation rate reported for this region, especially when compared to that of mitochondrial PCG´s [76].

**Fig. 5:**
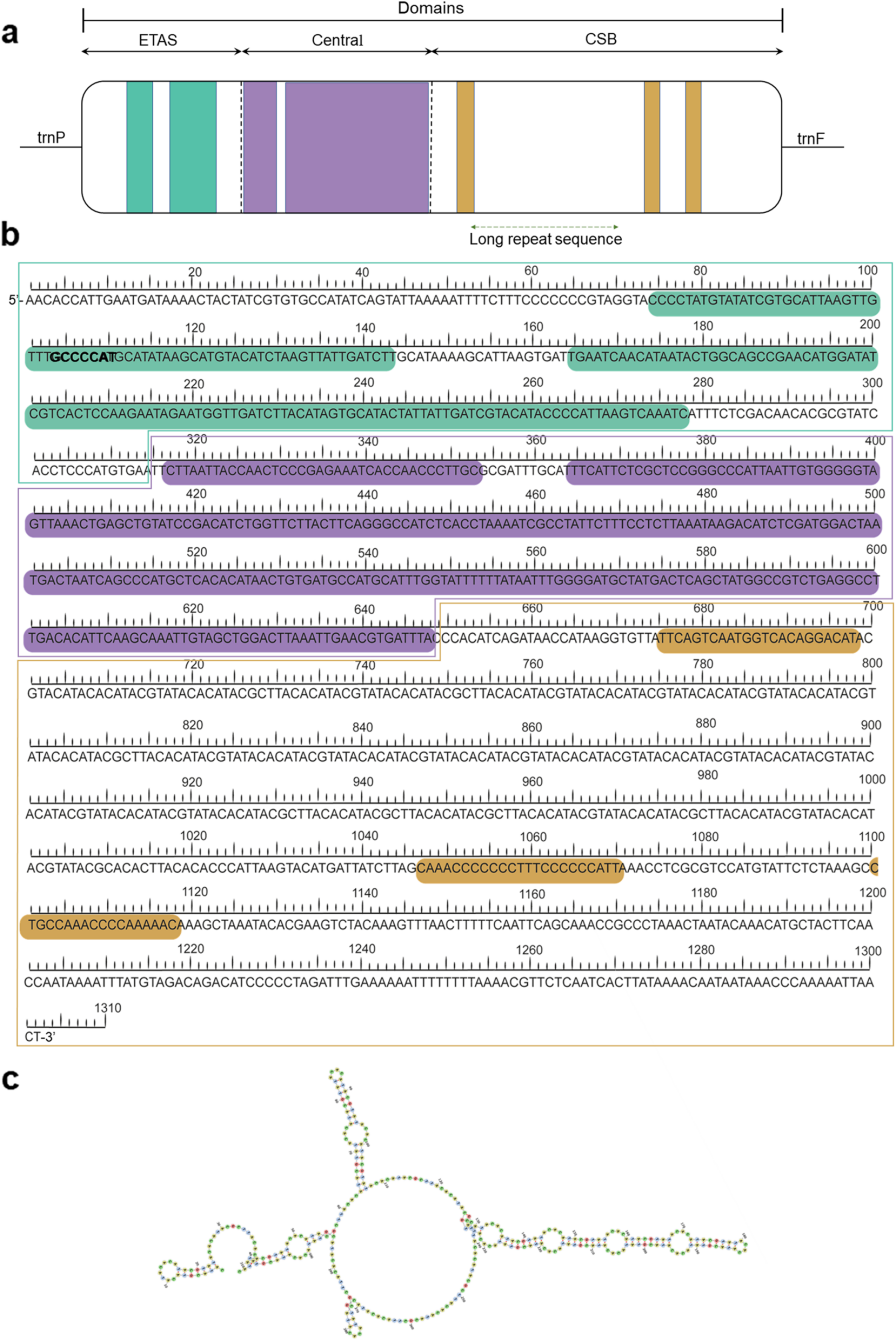
Organization and structure of the control region in the mitochondrial genome of *Tapirus pinchaque.* **a** Visual representation of the CR and the three functional domains (ETAS, central, and CSB domains). **b** Nucleotide sequence of the complete control region; locations of the ETAS-1 and ETAS-2 (underlined in green), the functional conserved motif 5'-GCCCCAT-3’ (in bold); the large highly conserved regions within the central domain (underlined in purple), as well as the CSB1, CSB2, and CSB3 blocks (underlined in orange) are shown. The long repetitive motif is indicated by the dotted green line and **c**) A possible secondary structure for this repetitive region is represented

Within the CR of *T. pinchaque*, eight microsatellites were detected, most of them dinucleotide motifs usually repeated three times (Supplementary Table S2). The existence of microsatellite repeats in the CR is characteristic of the mitochondrial genome in mammals [75, 77]. A tandem repeat sequence in the CR [5'-(ACA TAC GTA TAC)_26_–3' motif] was found to begin at position 16,143 and end at 16,456 of the complete mtDNA sequence (Fig. 5, Supplementary Table S3). Tandem repeats are also reported in the congeneric species *T. indicus* and *T. bairdii* [56, 57] as well as in other representatives of the Perissodactyla (e. g., *Equus caballus* and *Rhinoceros unicornis*) [59, 78]. We note that all these tandem repeats exhibit purine/pyrimidine alternation, a feature conserved in mammalian mtDNA’s [59, 74, 77]. A total of 20 possible secondary structures, all of them containing variable number and sizes of stem-loops (Supplementary Fig. S1), were revealed for the CR of *T. pinchaque*. The Gibbs free energy [ΔG] of these 20 structure predictions ranged in values between [ΔG] = -288.9 kcal/mol to [ΔG] = -287.6 kcal/mol (Supplementary Fig. S1).

The three functional domains within the CR, commonly reported in mammals [79], were recognized in *T. pinchaque* (Fig. 5). These are the central conserved domain and two flanking variable domains, namely the Extended Termination Associated Sequence (ETAS) domain and the Conserved Sequence Block (CSB) domain. The CSB, Central, and ETAS domains are 654, 334, and 315 bp in length, respectively. These domain lengths are within the range of those found in other tapir species, such as *T. indicus* and *T. bairdii* [56, 57].

The ETAS domain, located at the 5'-end of the CR, is A + T rich (63.2%) and contain extended ending associated sequence 1 (ETAS-1) and a second sequence homologous to the termination-associated sequence 2 (ETAS-2; Fig. 4). Within the ETAS-1 sequence, a functional conserved motif 5'-GCCCCAT-3' was identified (Fig. 5), which is known as a D-loop stop point [80]. Previous studies have also identified this motif in another mammalian CR [81, 82]. The Central domain sequence is also A + T rich (57.2%). A high degree of conservation was observed in the Central domain of the mtDNA of *T. pinchaque* and other species of *Tapirus* as well as other members of the Perissodactyla whose CR structure has been analyzed in detail [56, 79]. The CSB domain, positioned at the 5'-end of the CR, is A + T rich (64.2%) and contains three conserved sequence blocks (CSB-1, CSB-2, and CSB -3) that have been suggested to be functionally important for mtDNA replication and transcription [83]. We note that the relatively long tandem repeat [with motif: 5'-(ACATACGTATAC)_26_–3'] is located between the CSB-1 and CSB-2 regions (Fig. 4), as reported before in other odd-toed ungulates [56, 57, 78]. In general, detailed analyses of the CR in the order Perissodactyla are rare. Additional studies focusing on the CR organization are needed to further improve the understanding of the function of this region in mammals and beyond.

### Phylomitogenomics of odd-toed ungulates

**Fig. 6:**
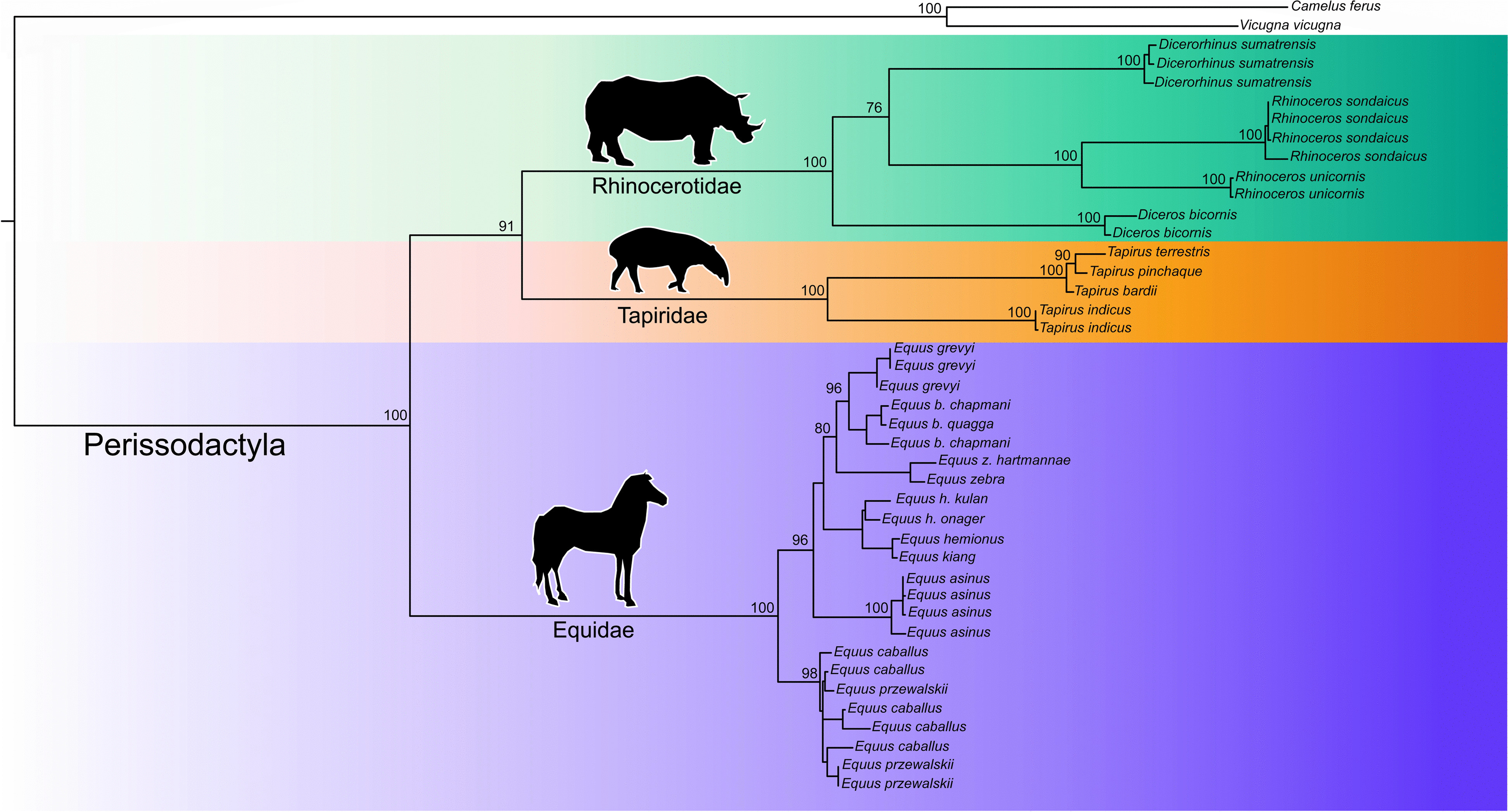
Phylogenetic position of *Tapirus pinchaque* and phylogenetic relationships in the order Perissodactyla. Phylogenetic analysis generated by Maximum Likelihood based on a concatenated alignment of amino acids belonging to the 13 protein-coding genes present in the mitochondrial genome of 40 representatives of the order Perissodactyla plus two outgroup species. Numbers near internal nodes above the branches represent bootstrap values

Our phylogenetic analysis using a Maximum Likelihood approach confirmed the monophyly of the order Perissodactyla; all representatives of the family Tapiridae, including *T. bardii*, *T*. *indicus*, *T. terrestris*, and *T. pinchaque*, all members of the family Equidae, and all species belonging to the family Rhinocerotidae used in our analysis clustered together into a single fully supported (bootstrap value [bv] = 100) clade (Fig. 6). In the Perissodactyla, the family Equidae occupied an early branching position; it was sister to a second clade comprising members of the families Rhinocerotidae and Tapiridae (bv = 91). Within the family Tapiridae, *T. indicus* occupied an early branching position; it was sister to a second fully supported monophyletic clade (bv = 100) comprising all three remaining species of *Tapirus*. In the latter clade, *T. bardii* was sister to a well-supported clade (bv = 98) containing *T*. *terrestris* and *T. pinchaque*. The phylogenetic relationships among the different species of Equidae and Rhinocerotidae are identical to those found by previous studies [56, 64, 84].

### The effect of altitude on mitochondrial adaptive evolution

**Fig. 7:**
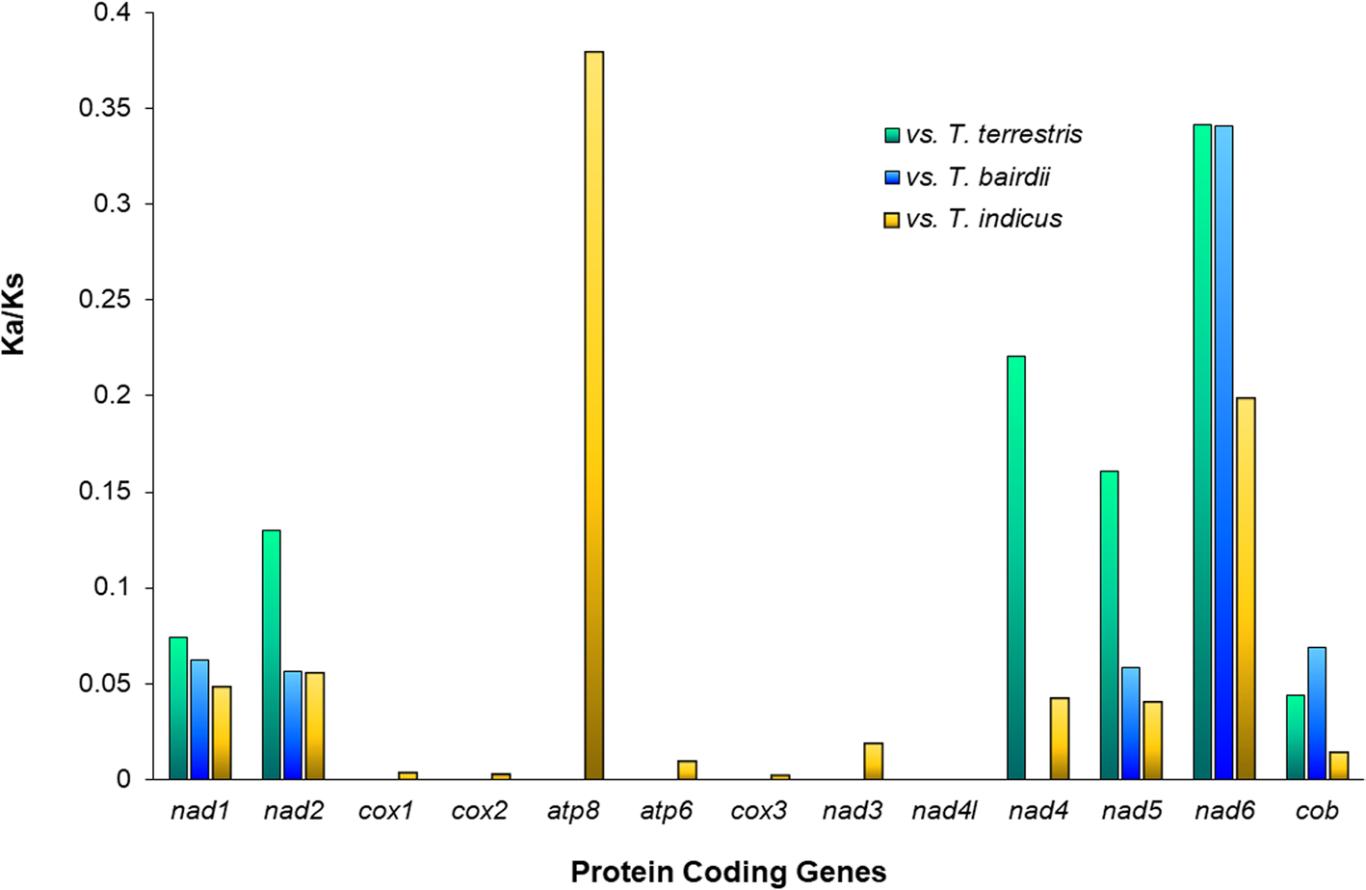
Analysis of selective pressure in the PCG´s of *Tapirus pinchaque vs*. other species of *Tapirus*. Value of the K_A_/K_S_ ratio for each of the 13 protein-coding genes present in the mitochondrial genome are provided

In our first selective pressure analysis, all the Ka/Ks ratios for all the 13 PCG´s of *T. pinchaque* exhibited values < 1, suggesting stronger purifying selection and evolutionary constraints in these mitochondrial genes (Fig. 7). Our results of the Ka/Ks comparisons between *T. pinchaque* and the other tapir species showed that *atp8* (Ka/Ks < 0.4; *p* > 0.05) and *nad6* (Ka/Ks < 0.35; *p* < 0.05) were the genes with the highest Ka/Ks values (Fig. 7). These results could be explained by the action of less intense purifying selection in *atp8* and *nad6* compared to the remaining mitochondrial PCG´s [85]. Regarding the lowest values (Ka/Ks ≤ 0; *p* ≤ 0.05), they were observed in the *nad4l* and in the cytochrome c oxidase subunit genes (*cox1*, *cox2*, and *cox3*; Fig. 7). Therefore, we suggest that the last four mentioned genes experience the strongest purifying selection. The results of the selective pressure analyses reported here are consistent with the results in all the PCG´s reported for *Tapirus bairdii* [56]. Until now, there have been few analyses of selective pressure in mitochondrial PCG´s within the family Tapiridae. Similarly, mitochondrial PCG´s selective pressure analyses are rare in the order Perissodactyla. A single previous report found a similar pattern of purifying selection of mitochondrial PCG´s in members of the genus *Equus* [86]. In general, previous research in mammals is also in agreement with the general pattern of mtDNA genes experiencing purifying selection, e. g., the representatives of the Caprini tribe [1], and several species of the family Mustelidae [87]. Our results agree with the hypothesis that purifying selection constraints, in general, the evolution of PCG´s, probably to maintain functionality of the OXPHOS pathway [19, 88].

In the second analysis performed to examine for signatures of positive selection in all 13 mitochondrial genes using BM’s in EasyCodeML, we observed that all the 13 mitochondrial PCG´s have values of ω < 1 in the foreground branches (Table 3). Based on likelihood ratio tests, nonetheless, the two-ratio model provided a statistically significant better fit than the one-ratio model in two genes (*cox3* [ω = 0.1136; *p* = 0.0489], and *nad6* [ω = 0.3150; *p* = 0.0155] Table 3). Our results suggest that *cox3* and *nad6* could be experiencing stronger purifiying selection than other mitochondrial PCG’s, and that putative amino acid changes occurring in these two genes might be fine-tuning the efficiency of proton translocation, modulation of the redox potential of Complex I, and the production of ROS in odd-toed ungulates inhabiting high altitude environments [89]. Our results contrast to what has been reported in other studies; subunits of the *COX* gene complex (e. g., *cox1* and *cox2* genes) and NADH gene complex (e. g., *nad2*, *nad3*, *nad5*, and *nad6* genes) may be under positive selection, at least to some extent, in the high-altitude Tibetan antelope (*Pantholops hodgsonii*), Tibetan horse (*E. caballus*), and plateau pika (*Ochotona curzoniae*) [2, 18, 90].

**Table 3:** Branch-Model results with EasyCodeML. Selective pressure analysis of the mitochondrial protein-coding genes (four high-altitude species as the foreground branches)

Gene	Model	Ln L	Parameter estimates		LRT *p*-value	ω for foreground branches
***atp6***	M2	-3635.876	ω0 = 0.033	ω1 = 0.010	0.482	-
	M0	-3636.123	ω = 0.033			
***atp8***	M2	-863.670	ω0 = 0.289	ω1 = 2.267	0.337	-
	M0	-863.208	ω = 0.288			
***cox1***	M2	-7087.656	ω0 = 0.005	ω1 = 0.010	0.6183	-
	M0	-7087.780	ω = 0.005			
***cox2***	M2	-3217.650	ω0 = 0.012	ω1 = 0.010	0.561	-
	M0	-3217.819	ω = 0.012			
***cox3***	M2	-3345.092	ω0 = 0.005	ω1 = 0.114	0.048	ω1 = 0.11356
	M0	-3347.030	ω = 0.006			
***cytb***	M2	-6018.723	ω0 = 0.015	ω1 = 0.063	0.116	-
	M0	-6019.97	ω = 0.015			
***nad1***	M2	-4453.074	ω0 = 0.011	ω1 = 0.114	0.123	-
	M0	-4454.265	ω = 0.011			
***nad2***	M2	-5078.249	ω0 = 0.036	ω1 = 0.042	0.896	-
	M0	-5078.257	ω = 0.036			
***nad3***	M2	-1915.324	ω0 = 0.022	ω1 = 119.627	0.983	-
	M0	-1915.324	ω = 0.021			
***nad4***	M2	-6990.917	ω0 = 0.021	ω1 = 0.010	0.309	-
	M0	-6991.435	ω = 0.021			
***nad4l***	M2	-1616.032	ω0 = 0.026	ω1 = 0.010	0.641	-
	M0	-1616.141	ω = 0.026			
***nad5***	M2	-9747.676	ω0 = 0.034	ω1 = 0.080	0.335	-
	M0	-9748.141	ω = 0.034			
***nad6***	M2	-2826.731	ω0 = 0.049	ω1 = 0.315	0.015	ω1 = 0.31501
	M0	-2829.661	ω = 0.052			

We also performed a BSM analysis to detect signatures of positive selection in the four high-altitude species (i. e., foreground branches) when compared to low-altitude species (background branches). For all PCG´s, model A (which allows positive selection; ω > 1) did not represent a better fit to our dataset compared to model A_null_ (which allows negative and neutral selection; 0 < ω < 1), as indicated by a Likehood Ratio Test (LRT) in all PCG´s (*p* > 0.05, Supplementary Table S4). Unexpectedly, and in contrast to our results, Wang et al. [91] detected signatures of positive selection, using the same BSM analysis, in a single mitochondrial gene (*nad5*) but using a disparate set of species that included mammal, aves, reptiles, amphibian, and ray-finned fishes.

Finally, SMs were used to identify sites of the sequences under positive selection in each PCG of the four high-altitude species of Perissodactyla. The M7 *vs.* M8 comparison showed high statistical significance (LRT test *p* < 0.01) in five different amino acid residues belonging to two PCG´s (Supplementary Table S5): the *nad2* gene with one amino-acid residue (Valine in position 157, Supplementary Table S5) and the *nad5* gene with four amino-acid residues (Serine in position 27; Asparagine in position 149; Threonine in position 420, and Glutamic Acid in position 532; Supplementary Table S5). Therefore, the existence of positive selection in these two genes is inferred; however, none of the sites exhibited significant posterior probability values greater than 0.95, as indicated by the Bayes empirical Bayes (BEB) approach. A previous study by Ning et al. [2] on different horse *Equus caballus* populations inhabiting different altitudes detected signatures of positive selection in two *nad6* residues (118 K and 162 L) from populations living above 2,000 masl.

In the web server DataMonkey [92], the RELAX analysis [93] indicated that *nad6* experienced a relaxation in selection strength (*k* = 0.34; LRT = 4.57; *p* = 0.032; Table 4) in high-altitude odd-toed ungulates. In turn, no indication that the strength of natural selection relaxed or intensified along branches leading to high-altitude odd-toed ungulates was found in the other 12 mitochondrial PCG´s (all LRT´s with *p* > 0.05; Table 4). The adaptive Branch-Site Random Effects Likelihood (aBSREL) [94] analysis found no evidence of episodic diversifying selection in any of the 13 mitochondrial PCG´s in high-altitude odd-toed ungulates. On the other hand, the Branch-Site Unrestricted Statistical Test for Episodic Diversification (BUSTED) analysis [95] (that included site-to-site synonymous rate variation) provided evidence of episodic diversifying selection in *cob* (LRT, *p* = 0.038) in high-altitude odd-toed ungulates (Table 4). The Mixed Effects Model of Evolution (MEME) analysis [96] revealed statistically significant episodic positive / diversifying selection signature in two sites (245; *p* = 0.048 and 257; *p* = 0.040) in *nad1*, one site (152; *p* = 0.046) in *nad2*, and one site (100; *p* = 0.040) in *nad4* gene (Table 4). The Fast Unconstrained Bayesian AppRoximation (FUBAR) analysis [97] found no evidence of sites experiencing positive selection in any of the mitochondrial PCG´s. Instead, a large number of sites under negative selection was detected in most of the 13 mitochondrial genes, with *cox1* exhibiting the largest number of sites (*n* = 176) (Table 4) followed by *cob* and *nad4* (164 and 104 sites, respectively) under purifying selection (Table 4). Lastly, a Single-Likelihood Ancestor Counting (SLAC) analysis [98] found evidence of codons undergoing purifying selection (ω < 1) at sites 158 (*p* = 0.037) and 184 (*p* = 0.049) of the *cob* and *nad5* genes, respectively (Table 4).

**Table 4:** Estimation of selection signatures using different models in the webserver DataMonkey. K denotes selection intensity parameter; ω < 1 indicates purifying selection; ω > 1 indicates diversifying selection; > 0.95 indicates significant posterior probability

**gene**	**BM**	**BSM**		**SM**		
	**RELAX**	**aBSREL**	**BUSTED**	**MEME**	**FUBAR**	**SLAC**
	**K value (*p*-value;LR)**	**# sites (*p*-value)**		**sites ω > 1 (*p*-value)**	**# sites ω < 1 (> 0.95)**	**site ω < 1 (*p*-value)**
***atp6***	33.54 (0.337; 0.92)	–	–	–	27	–
***atp8***	0.23 (0.229; 1.45)	–	–	–	1	–
***cox1***	0.82(0.88; 0.02)	–	–	–	176	–
***cox2***	11.77 (0.723; 0.13)	–	–	–	62	–
***cox3***	5.82 (0.080; 3.07)	–	–	–	18	–
***cob***	1.99 (0.156; 2.01)	–	**1 (0.038)**	–	164	**158 (0.037)**
***nad1***	1.68 (0.261; 1.26)	–	–	**245 (0.048), 257 (0.040)**	37	–
***nad2***	5.43 (0.899; 0.02)	–	–	**152 (0.046)**	33	–
***nad3***	0.93 (1.0; 0.00)	–	–	–	10	–
***nad4***	1.09 (0.924; 0.01)	–	–	**100 (0.040)**	104	–
***nad4l***	43.17 (0.316; 1.0)	–	–	–	5	–
***nad5***	0.41 (0.205; 1.61)	–	–	–	82	**184 (0.049)**
***nad6***	**0.34 (0.032; 4.57)**	–	–	–	9	–

We have explored if adaptive evolution occurs in mitochondrial PCG´s of *T*. *pinchaque* and other odd-toed ungulates inhabiting high-altitude environments and found a signature of positive selective pressure at various residues in different PCG´s. Our results are in line with previous studies examining adaptive evolution in PCG´s of odd-toed ungulates (e. g., [13, 18, 99]) and other vertebrates and invertebrates colonizing harsh environments (e. g., [100–102]). Furthermore, previous studies have found evidence of adaptive evolution in mitochondrial PCG´s in bats [103], birds [104], snakes [105], and penguins [106], among other vertebrates [19]. Overall, our studies support the notion that changes in physiological/metabolic demand and other environmental conditions (e. g., temperature, oxygen availability) in vertebrates may result in selective pressures acting upon mitochondrial-encoded genes to meet the demands of the animal´s lifestyles and habitats in which they live [88]. The potential for adaptive evolution of mitochondrial PCG´s needs to be considered when exploring for evolutionary significant units in odd-toed ungulates and other species of conservation concern.

## Conclusions

In summary, we have assembled, annotated, and characterized in detail the complete mtDNA of the smallest *Tapirus* species, the mountain wooly tapir, *T. pinchaque*. We inferred that, overall, all 13 mitochondrial PCG´s are exposed to negative / purifying selection. Nonetheless, further analyses of adaptive molecular evolution indicated that some residues experience adaptive evolution in species of odd-toed ungulates that have colonized high-altitude environments. Specifically, Branch Models indicated that *cox3* and *nad6* might be undergoing strong purifying selection in ungulates colonizing high-altitude habitats. Furthermore, Site Models suggested that one and four sites in *nad2* and *nad5*, respectively, could be experiencing positive selection. Lastly, additional analyses (RELAX, aBSREL, BUSTED, MEME, FUBAR, and SLAC) indicated a relaxation of selection strength in *nad6*, evidence of episodic diversifying selection in *cob*, and revealed episodic positive/diversifying selection signatures for two sites in *nad1*, one site each in *nad2* and *nad4*. The complete mitochondrial genome of *T. pinchaque* will support conservation initiatives in this remarkable mammal (i. e., non-intrusive bioprospecting and biomonitoring, see [107]) and our results indicate that the colonization of high-altitude habitats have resulted, at least to some extent, in the adaptive evolution of mitochondrial PCG´s in *T. pinchaque* and other odd-toed ungulates.

## Methods

### Field collection and sequencing

A hair sample preserved in 70% ethanol belonging to one specimen of *T. pinchaque* was donated to one of us (JO) by the Instituto de Ciências Biológicas da Universidade Federal de Minas Gerais, Minas Gerais, Brazil. Total genomic DNA was extracted from the hair sample with the Qiagen Blood and Tissue Kit (Qiagen, Hilden, Germany) using the manufacturer's instructions. Extracted DNA was then shipped to the Savannah River Ecology Laboratory, University of Georgia, Aiken, for next-generation sequencing. Illumina paired-end (PE) shotgun libraries were prepared using the standard protocol of the Nextera™ DNA Sample Prep Kit (Epicentre®) and sequenced in an Illumina HiSeq sequencer (Illumina, San Diego, CA, USA) using 2 × 200 cycles. A total of 5,507,122 pairs of reads were generated for *T. pinchaque* and made available in FASTQ format by the sequencing facility. The totality of these reads was used for assembling the mitochondrial genome.

### Mitochondrial genome assembly

The mitochondrial genome of *T. pinchaque* was de novo-assembled using the pipeline GetOrganelle v1.6.4 [54]. The mitochondrial genome of the congeneric *T. terrestris*, available in GenBank (KJ417810), was used as a seed. The run used k-mer sizes of 21, 55, 85, and 115.

### Mitochondrial genome annotation and characterization

The newly assembled mitochondrial genome was first annotated in silico using the web servers MITOS and MITOS2 (http://mitos2.bioinf.uni-leipzig.de/index.py) [108] with the vertebrate genetic code. Annotation and manual curation of all the features found in the mitochondrial genome, including the PCG´s start and stop codons corrections, were carried out using the ExPASy v. 3.0 translate tool (https://web.expasy.org/translate/) [109] and MEGA v. X [110]. Subsequent visualization of each manually curated mitochondrial genome was performed with the Chloroplot webserver (https://www.frontiersin.org/articles/10.3389/fgene.2020.576124/full) [111].

The nucleotide composition of the mitochondrial genome was estimated in the software MEGA v. X. A codon usage analysis of the PCG´s from the studied species was conducted using the Sequence Manipulation Suite: Codon Usage online tool v. 2 (https://www.bioinformatics.org/sms2/codon_usage.html) [112]. Next, EZcodon, as implemented in the web server EZmito v. 2021.11 (http://ezmito.unisi.it/ezcodon) [113], was used to calculate Relative Synonymous Codon Usage (RSCU). RSCU is defined as the ratio of the observed frequency of codons to the expected frequency given that all the synonymous codons for the same amino acid are used equally [69, 71].

The secondary structure of each tRNA was predicted using the software MiTFi [114] as implemented in MITOS2 (http://mitos2.bioinf.uni-leipzig.de/ index.py) [115] and the web server Forna (http://rna.tbi.univie.ac.at/forna/) [72] was used for visualization of each tRNA.

The putative CR of the mitochondrial genomes was examined in detail, first using the BioPHP-microsatellite repeat finder (http://insilico.ehu.es/mini_tools/microsatellites/) [116] web server to detect microsatellites. The CR was then analyzed using the web server Tandem Repeat Finder v. 4.09.1 (https://tandem.bu.edu/trf/trf.html) [117] to detect repetitive tandem sequences. Lastly, secondary structure predictions of the CR in the mitochondrial genome were modeled using the RNA structure Web Server tool v. 6.4 (http://rna.urmc.rochester.edu/RNAstructureWeb) [118]. All default options were used for exploring the secondary structure of the CR, except for the temperature that was set to 285.15°K for *T. pinchaque*, representing the ‘thermal’ environment inhabited by this species.

### Phylomitogenomics of odd-toed ungulates

We examined the phylogenetic position of *T. pinchaque* among other representatives of the genus *Tapirus* and Perissodactyla based on translated PCG´s, following the protocol detailed in Ennis et al. [56]. The newly assembled mitochondrial genome of *T. pinchaque* together with the mitochondrial genomes of 39 other specimens belonging to extant odd-toed ungulates available in the NCBI’s GenBank database were used for phylogenetic reconstruction using the software MitoPhAST v. 3 [119]. Two different species of artiodactyls (order Artiodactyla), namely, *Vicugna vicugna*, and *Camelus ferus*, belonging to the family Camelidae, were used as outgroups during the phylogenetic analysis. The software MitoPhAST first extracted all 13 PCG´s nucleotide sequences from the species available in GenBank as well as those from *T*. *pinchaque* and aligned each set of PCG nucleotide sequences after translating them into amino acids with the software Clustal Omega v. 1.2.4 [120]. Next, poorly aligned regions were removed with the software trimAl v. 1.2 [121] to then partition the dataset and estimate the best fitting models of sequence evolution using the program ProtTest v. 3.4.2 [122]. Finally, the concatenated and partitioned PCG amino acid alignments were used to conduct a maximum likelihood (ML) phylogenetic tree analysis in the software IQ-TREE v. 1.6.12 [123]. The robustness of the ML tree topology was ascertained by 1,000 bootstrap pseudoreplicates of the tree search.

### Selective pressure analyses

First, we measured selective pressures acting on each of the 13 PCG´s in the studied species. For this purpose, the number of non-synonymous substitutions per non-synonymous site (Ka), synonymous substitutions per synonymous site (Ks), and the Ka / Ks ratio were estimated using the software KaKs_calculator v. 2.0 [124]. Neutral selection is given by Ka /Ks = 1; while Ka / Ks < 1 denotes purifying (negative) selection, and Ka / Ks > 1 implies diversifying (positive) selection in a particular PCG [124]. For this analysis, Ka and Ks values were based on pairwise comparisons between each PCG sequence from the species herein studied, and all other species of *Tapirus* with mitochondrial genomes available in GenBank (*T. terrestris*, NC_053962.1; *T. indicus*, KJ417810.1 and *T. bairdii*, NC_063943). The γ-MYN was used during calculations to account for variable mutation rates along the length of each gene sequence [124].

Next, we examined for signatures of positive selection in all 13 mitochondrial PCG´s that could be driven by colonization of high-altitude environments in *T. pinchaque* and other representatives of the Perissodactyla using the program EasyCodeML v.1.0 [50, 125]. EasyCodeML is a codon-based method for detecting signatures of positive selection that estimates ω (d_N_/d_S_) across a phylogenetic tree. We used as input files, the phylogeny previously obtained with MitoPhAST and the multiple sequence alignments of each mitochondrial PCG´s with the software Muscle v. 5 as implemented in MEGA. The possible effect of a particular ecological condition (e. g., high-altitude in this study) on selective pressures proceeds after the user assigns ‘foreground’ and ‘background’ branches in the phylogenetic tree, with EasyCodeML [126]. Foreground branches correspond to those leading to terminal nodes (species) that live in high-altitude habitats: represented by *T. pinchaque*, *T. bairdii*, *Equus burchellii quagga*, and *Equus kiang* in our study. In turn, background branches are those leading to 36 other terminals from representatives of the Perissodactyla including in the phylogeny and inhabiting regions below 2000 masl.

In EasyCodeML, we first used Branch Models (BM) [126] to test for statistically significant differences in ω among branches of the phylogenetic tree. We fitted three different models to the sequence dataset (aligned amino acids strings for each PCG separately), namely, the one-ratio model, the two-ratio model, and the free-ratio model. The one-ratio model (M0) assumes that ω is constant throughout the phylogenetic tree, the free-ratio model allows an independent ω for each branch in the tree, and the two-ratio model assumes that specific (e. g., foreground) branches have an ω that is different from that throughout the rest of the (e. g., background) branches in the phylogenetic tree [125, 127]. We conducted pairwise comparisons among the three different models using independent LRT’s [128]. The degrees of freedom were the difference in the number of free parameters between models. Evidence for positive selection and the effect of a particular environmental condition on PCG´s adaptive evolution is provided when the two-ratio model provides a better fit than the M0 model to the sequence data [126]. Also, heterogeneity in the dN/dS ratio between lineages can be evidenced when comparing the free-ratio model against the M0. This heterogeneity between lineages may be due to positive selection or relaxed selection constraints [127].

Considering that positive selection can often act on only a few sites and during short periods of evolutionary time, we also used the BSM in EasyCodeML to test for statistically significant differences in ω among sites along pre-specified lineages in our phylogeny [49]. The BSM assume heterogeneous ω across sites and across branches [125]. Two models were fitted to our datasets (aligned PCG´s): the model A and the model A_null_ [49, 125]. Model A allows positive selection (with ω > 1) to occur along pre-specified branches (i. e., foreground branches) while for the other branches (i. e., background branches) only negative selection and neutral evolution (0 < ω < 1) is allowed. In turn, Model A_null_ allows negative selection (i. e., 0 < ω < 1) and neutral evolution (i. e., ω = 1) to occur along all branches [49, 125]. We conducted pairwise comparisons among the two different models using independent LRT’s [128]. We predicted Model A to be a better fit to our dataset than the null model if high-altitude environments drive adaptive evolution of codons along branches in odd-toed ungulates.

Lastly, we used the SM in EasyCodeML, to investigate selection pressure among sites of each mitochondrial gene sequence [126]. We fitted six different codon substitution models to our dataset: M0 (one ω ratio for all sites), M1a (nearly neutral, ω = 1), M2a (positive selection, ω > 1), M7 (ω varies according to β distribution, 0 < ω < 1), M8 (β and ω > 1), and M8a (β and ω = 1) in which ω-ratio is allowed to vary among sites [49, 126]. Two pairs of site model contrasts (M1a *vs*. M2a and M7 *vs.* M8) are particularly effective for the detection of positive selection [128]. Support for positive selection can be identified if M2a provides a better fit than M1a. Similarly, positive selection can be identified if the M8 model provides a better fit than M7 or M8a [125]. The M8 vs. M7 offers a very stringent test of positive selection [126; 129]. We conducted pairwise comparisons among the different models using independent LTR [128, 129]. Finally, we apply the BEB method to identify sites under positive selection or relaxed from purifying selection in foreground branches with significant LRTs in the SM test [129].

In addition to our analyses in EasyCodeML, we use codon models in the HyPhy v. 2.0 package [130] as implemented on the web server DataMonkey v. 1.10.0 (http://www.datamonkey.org/) [92] to detect signatures of adaptive evolution in mitochondrial PCG´s from odd-toed ungulates inhabiting high-altitude environments. One BM (i. e., RELAX) [93] and two BSMs (aBSREL [94] and BUSTED [95]) were executed with the same input dataset previously used in the EasycodeML analyses that contained aligned sequences from the four species of interest plus the other 38 odd-toed ungulates (including the two species as outgroup) from low-altitude habitats.

RELAX determines if the strength of selection has relaxed or intensified along pre-specified branches (i. e., leading to high-altitude odd-toed ungulates) in a phylogenetic tree [93]. RELAX fits two models (null, with three ω classes applied to the entire phylogeny vs. alternative, introducing the selection intensity parameter *k* and modified and fixed ω^*k*^ classes) to the dataset and uses the LRT to compare the null and alternative models. The parameter *k* estimated in RELAX represents a measure of selection strength with values greater or lower than 1 indicating that selection strength has intensified or relaxed, respectively [93]. A better fit of the alternative model to the dataset is useful for identifying shifts in the stringency of natural selection on a given gene [92]. Neighbor-Joining trees used for the calculation were automatically generated in the web server DataMonkey using the General Time-Reversible (GTR) substitution model.

aBSREL tests whether a proportion of sites have evolved under positive selection in pre-specified branches (i. e., leading to high-altitude odd-toed ungulates) in a phylogeny [92]. aBSREL models’ heterogeneity in ω at both site and branch levels and uses the corrected Akaike's Information Criterion (AICc) to estimate the ‘optimal’ number of ω rate classes for each branch in the phylogeny. After fitting a full adaptive model with the ‘optimal’ number of ω rate classes to the dataset, an LRT is performed to compare the fit of the full model against a null model that does not allow branches to experience positive selection in the phylogeny [93]. In turn, BUSTED identifies whether a gene has undergone positive selection in at least one site and at least one branch of interest in a phylogeny. As in previous methods, BUSTED compares the fit of two models to the dataset; an alternative model (also called unrestricted model) against a null model (or constrained model) that does not allow positive selection on the branches of interest. In addition, BUSTED calculates "Evidence Ratios" (ER) for each site. The ER is a likelihood ratio that the unrestricted (alternative) model represents a better fit to the dataset compared to the null (constrained) model [95].

Lastly, three different Site-Model analyzes were executed using only the four high-altitude odd-toed ungulates as an input file: MEME [96], FUBAR (Fast, Unconstrained Bayesian AppRoximation [97], and SLAC (Single-Likelihood Ancestor Counting [98]. MEME tests whether sites in a set of branches evolve under positive or diversifying episodic selection and employs a mixed-effects maximum likelihood approach to infer two ω-rate classes and the probability that each site will evolve under each respective ω-rate class [96]. We selected a *p*-value < 0.05 to decrease false positive rates in this test [96]. FUBAR uses a Bayesian approach to infer dN and dS per site in each alignment and reports posterior probabilities (ranging from 0 to 1) to determine the existence of positive selection [97]. We changed the default posterior probability value from 0.9 to 0.95 to minimize false positive results in our analyses [97]. SLAC identifies positive and negative selection at specific sites in a codon alignment through a combination of maximum likelihood and a counting (Suzuki-Gojobori modified version) approach to infer dN and dS per site [98].

### Supplementary Information

**Additional file 1:** **Table S1.** Codon usage analysis of the protein coding genes in the mitochondrial DNA of *Tapirus pinchaque*. **Table S2.** Characteristics of the microsatellite repeat sequences detected in the control region of the mtDNA of *T. Pinchaque*. **Table S3. **Tandem repeat sequence detected in the Control Region of the mtDNA of *Tapirus pinchaque*.

**Additional file 2:** **Supplementary Figure S1.** Secondary structure predictions of the Control Region.

**Additional file 3:** **Table S4.** Branch-Site Model results with EasyCodeML. Selective pressure analysis of the mitochondrial protein-coding genes (four high-altitude species as the foreground branches). **Table S5.** Site-Model results with EasyCodeML. Selective pressure analysis of the mitochondrial protein-coding genes (four high-altitude species as the foreground branches).

## Data Availability

Complete mitochondrial genome sequence data have been deposited in the NCBI GenBank with accession no. OQ420428.

## Abbreviations

aBSREL: Adaptive Branch-Site Random Effects Likelihood
AICc: Akaike's Information Criterion
Ala: Alanine amino acid
Arg: Arginine amino acid
ATP: Adenosine triphosphate
atp: Subunits of ATP synthase
BEB: Bayesian Empirical Bayes
BM: Branch Model
BSM: Branch-Site Model
BUSTED: Branch-Site Unrestricted Statistical Test for Episodic Diversification
bv: Bootstrap value
cm: Centimeters
cob: Cytochrome b subunit of the cytochrome bc1 complex
cox: Subunits of the cytochrome c oxidase
CR: Control Region
CSB: Conserved Sequence Block
d_N_: Non-synonymous substituions
d_S_: Synonymous substitutions
EGLN1: Egl-9 family hypoxia inducible factor 1
EPAS1: Endothelial PAS domain protein 1
ER: Evidence Ratio
ETAS: Extended Termination Associated Sequence
FUBAR: Fast, Unconstrained Bayesian AppRoximation
Gln: Glutamine aminoacid
GTR: General Time-Reversible
H: Heavy strand
H_2_O_2_: Hydrogen peroxide
HIF-1A: Hypoxia-inducible factor-1 alpha
Ka: Non-synonymous substitutions per non-synonymous site
Ks: Synonymous substitutions per synonymous site
L: Negative or light strand
Leu: Leucine aminoacid
LRT: Likehood Ratio Test
Lys: Lysine amino acid
m: Meters
M0: One-ratio model
masl: Meters above sea level
MEME: Mixed Effects Model of Evolution
ML: Maximun Likelihood
mtDNA: Mitochondrial genome
nad: Subunits of the NADH dehydrogenase complex
NCBI: National Center for Biotechnology Information
O_2_^−^: Superoxide
OXPHOS: Oxidative phosphorylation system
PCG´s: Protein coding genes
PE: Paired-end
ROS: Reactive oxygen species
rRNA: Ribosomal genes
rrnL: 16S ribosomal RNA
rrnS: 12S ribosomal RNA
RSCU: Relative Synonymous Codon Usage
Ser: Serine amino acid
SLAC: Single-Likelihood Ancestor Counting
SM: Site Model
T: Incomplete stop codon
Thr: Threonine amino acid
tRNA: Transfer RNA genes
Trp: Tryptophan amino acid
Val: Valine aminoacid
ω = d_N_/d_S_ = Ka/Ks: The ratio of non-synonymous to synonymous substitutions
