# Supplementary material for: The mitochondrial genome of the mountain wooly tapir, Tapirus pinchaque and a formal test of the effect of altitude on the adaptive evolution of mitochondrial protein coding genes in odd-toed ungulates
Source: BMC Genomics. 2023 Sep 6;24:527. doi: 10.1186/s12864-023-09596-8 (PMC10481570; doi:10.1186/s12864-023-09596-8)
Supplement: Supplementary file 1 — Additional file 1: Table S1. Codon usage analysis of the protein coding genes in the mitochondrial DNA of Tapirus pinchaque. Table S2. Characteristics of the microsatellite repeat sequences detected in the control region of the mtDNA of T. Pinchaque. Table S3. Tandem repeat sequence detected in the Control Region of the mtDNA of Tapirus pinchaque. [file 12864_2023_9596_MOESM1_ESM.pdf]

Conservation Genetics

The mitochondrial genome of the mountain wooly tapir, *Tapirus pinchaque* and a formal test of the effect of altitude on the adaptive evolution of mitochondrial protein coding genes in odd-toed ungulates

Edgar G. Gutiérrez[1], Jorge Ortega[1], and J. Antonio Baeza[2,3,4 *]

1Laboratorio de Bioconservación y Manejo, Posgrado en Ciencias Químicobiológicas, Departamento de Zoología, Escuela Nacional de Ciencias Biológicas, Instituto Politécnico Nacional, Prolongación Carpio y Plan de Ayala s/n, Col. Santo Tomás, C.P. 11340, Ciudad de México, México.
2 Department of Biological Sciences, 132 Long Hall, Clemson University, Clemson, SC 29634, USA.
3 Smithsonian Marine Station at Fort Pierce, 701 Seaway Drive, Fort Pierce, FL 34949, USA.
4 Departamento de Biología Marina, Facultad de Ciencias del Mar, Universidad Católica del Norte, Larrondo 1281, Coquimbo, Chile.

*Correspondence author: Email: baeza.antonio@gmail.com

Table S1. Codon usage analysis of the protein coding genes in the mitochondrial DNA of *Tapirus pinchaque*

AminoAcid	Codon	Number	/1000	Fraction
Ala	GCG	7.00	1.84	0.03
Ala	GCA	91.00	23.96	0.39
Ala	GCT	66.00	17.38	0.28
Ala	GCC	70.00	18.43	0.30
Cys	TGT	7.00	1.84	0.30
Cys	TGC	16.00	4.21	0.70
Asp	GAT	35.00	9.22	0.52
Asp	GAC	32.00	8.43	0.48
Glu	GAG	10.00	2.63	0.10
Glu	GAA	86.00	22.64	0.90
Phe	TTT	109.00	28.7	0.46
Phe	TTC	126.00	33.18	0.54
Gly	GGG	19.00	5.00	0.09
Gly	GGA	100.00	26.33	0.46
Gly	GGT	39.00	10.27	0.18
Gly	GGC	58.00	15.27	0.27
His	CAT	29.00	7.64	0.29
His	CAC	71.00	18.69	0.71
Ile	ATT	180.00	47.39	0.52
Ile	ATC	165.00	43.44	0.48
Lys	AAG	6.00	1.58	0.06
Lys	AAA	93.00	24.49	0.94
Leu	TTG	14.00	3.69	0.02
Leu	TTA	138.00	36.33	0.23
Leu	CTG	15.00	3.95	0.02
Leu	CTA	287.00	75.57	0.47
Leu	CTT	75.00	19.75	0.12
Leu	CTC	82.00	21.59	0.13

Met	ATG	32.00	8.43	0.14
Met	ATA	204.00	53.71	0.86
Asn	AAT	62.00	16.32	0.40
Asn	AAC	94.00	24.75	0.60
Pro	CCG	6.00	1.58	0.03
Pro	CCA	96.00	25.28	0.50
Pro	CCT	45.00	11.85	0.24
Pro	CCC	44.00	11.59	0.23
Gln	CAG	5.00	1.32	0.06
Gln	CAA	85.00	22.38	0.94
Arg	CGG	1.00	0.26	0.02
Arg	CGA	38.00	10.01	0.61
Arg	CGT	9.00	2.37	0.15
Arg	CGC	14.00	3.69	0.23
Ser	AGT	21.00	5.53	0.07
Ser	AGC	32.00	8.43	0.11
Ser	TCG	5.00	1.32	0.02
Ser	TCA	119.00	31.33	0.42
Ser	TCT	46.00	12.11	0.16
Ser	TCC	62.00	16.32	0.22
Thr	ACG	9.00	2.37	0.03
Thr	ACA	173.00	45.55	0.53
Thr	ACT	64.00	16.85	0.20
Thr	ACC	80.00	21.06	0.25
Val	GTG	17.00	4.48	0.09
Val	GTA	91.00	23.96	0.51
Val	GTT	49.00	12.9	0.27
Val	GTC	23.00	6.06	0.13
Trp	TGG	2.00	0.53	0.02
Trp	TGA	103.00	27.12	0.98
Tyr	TAT	70.00	18.43	0.053
Tyr	TAC	62.00	16.32	0.47
End	AGG	0.00	0.00	0.00
End	AGA	1.00	0.26	0.11

End	TAG	0.00	0.00	0.00
End	TAA	8.00	2.11	0.89

Table S2. Characteristics of the microsatellite repeat sequences detected in the control region of the mtDNA of *T. pinchaque*

Position	Cycle	Repeats	Sequence
59	2	3	CCCCCC
519	2	3	CACACA
550	2	3	TTTTTT
1016	2	3	ACACAC
1050	2	3	CCCCCC
1060	2	3	CCCCCC
1239	2	3	AAAAAA
1246	2	3	TTTTTT

Table S3. Tandem repeat sequence detected in the Control Region of the mtDNA of *Tapirus pinchaque*

Indices	Period Size	Copy Number	Consensus Size	Percent Matches	Percent Indels	Score	A	C	G	T	Entropy (0-2)
695-1008	12	26.0	12	92	7	498	39%	27%	8%	24%	1.84
