# Supplementary material for: The mitochondrial genome of the mountain wooly tapir, Tapirus pinchaque and a formal test of the effect of altitude on the adaptive evolution of mitochondrial protein coding genes in odd-toed ungulates
Source: BMC Genomics. 2023 Sep 6;24:527. doi: 10.1186/s12864-023-09596-8 (PMC10481570; doi:10.1186/s12864-023-09596-8)
Supplement: Supplementary file 2 — Additional file 2: Supplementary Figure S1. Secondary structure predictions of the Control Region. [file 12864_2023_9596_MOESM2_ESM.zip › 12864_2023_9596_MOESM3_ESM.pdf]

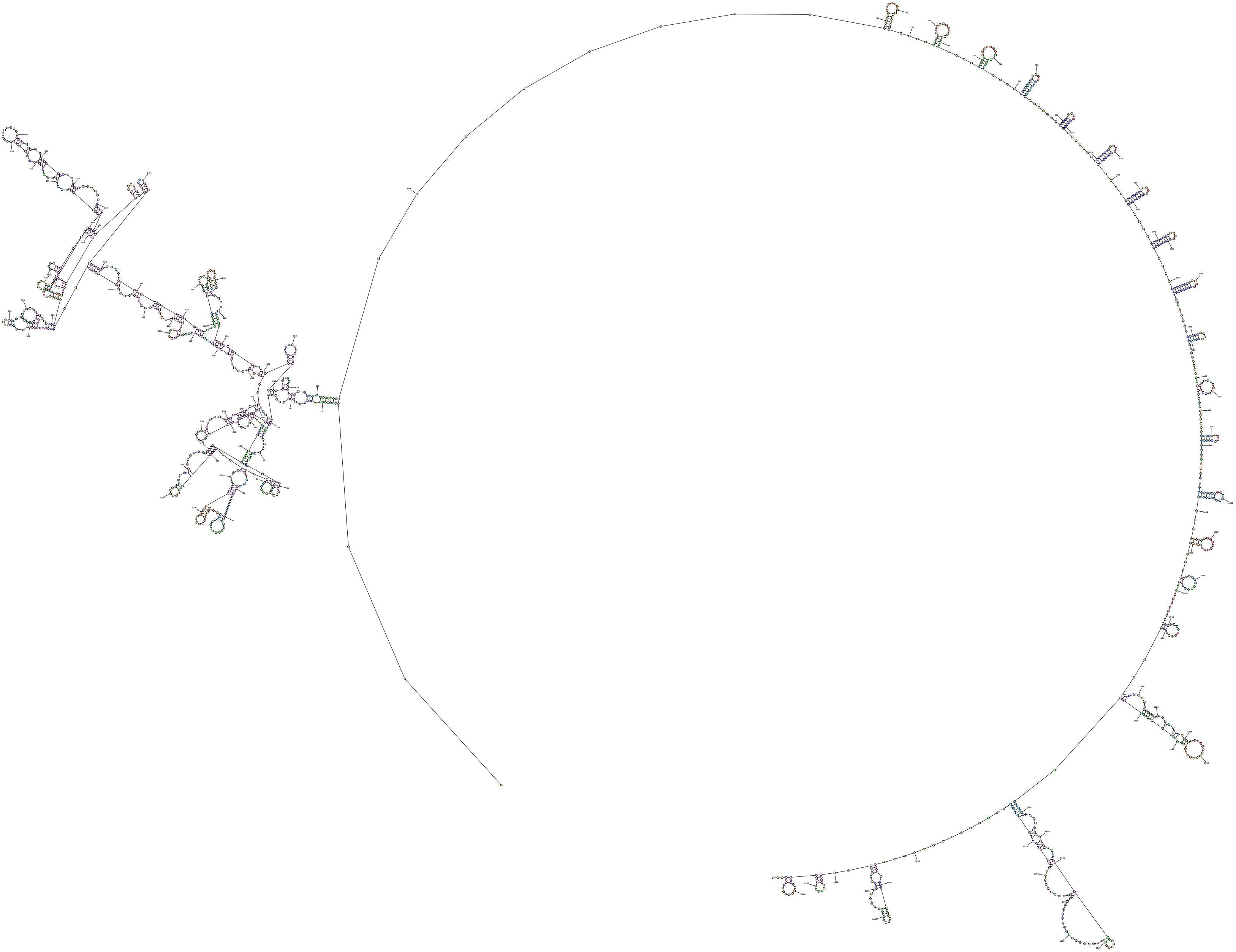

Probability >= 99%
99% > Probability >= 95%
95% > Probability >= 90%
90% > Probability >= 80%
80% > Probability >= 70%
70% > Probability >= 60%
60% > Probability >= 50%
50% > Probability

ENERGY = -288.9 CR_T_pinchaque

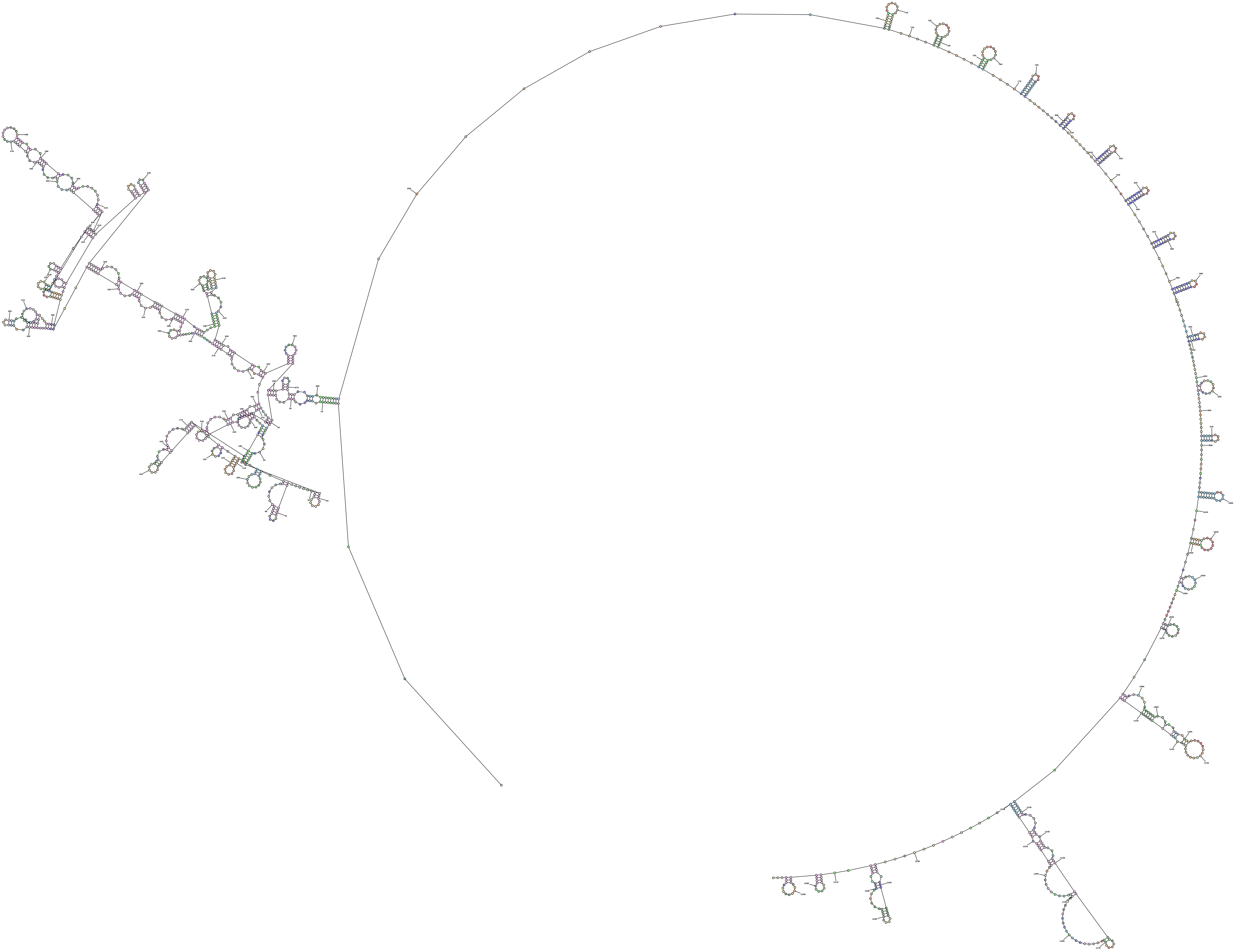

Probability >= 99%
99% > Probability >= 95%
95% > Probability >= 90%
90% > Probability >= 80%
80% > Probability >= 70%
70% > Probability >= 60%
60% > Probability >= 50%
50% > Probability

ENERGY = -288.9 CR_T_pinchaque

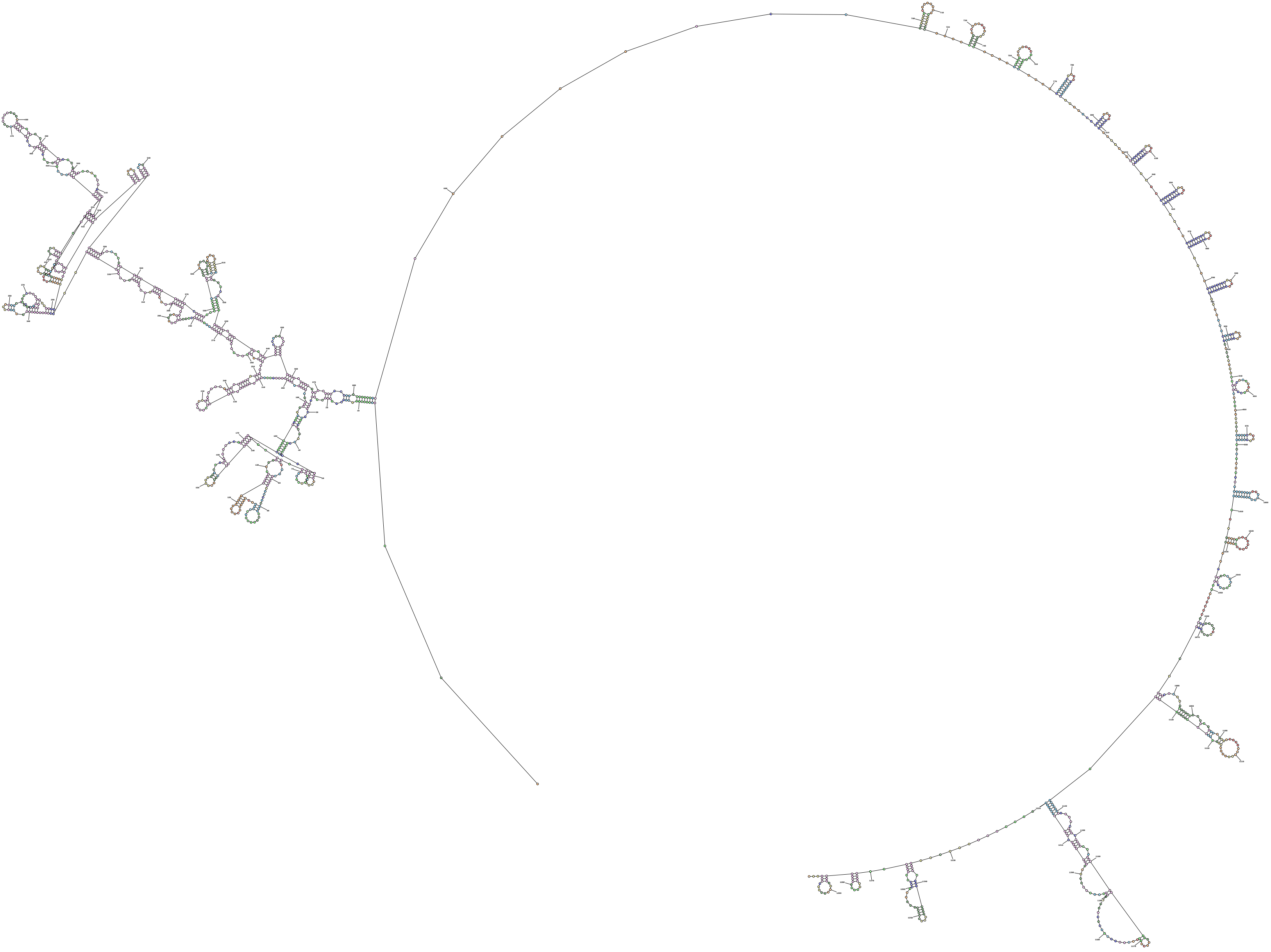

Probability >= 99%
99% > Probability >= 95%
95% > Probability >= 90%
90% > Probability >= 80%
80% > Probability >= 70%
70% > Probability >= 60%
60% > Probability >= 50%
50% > Probability

ENERGY = -288.6 CR_T_pinchaque

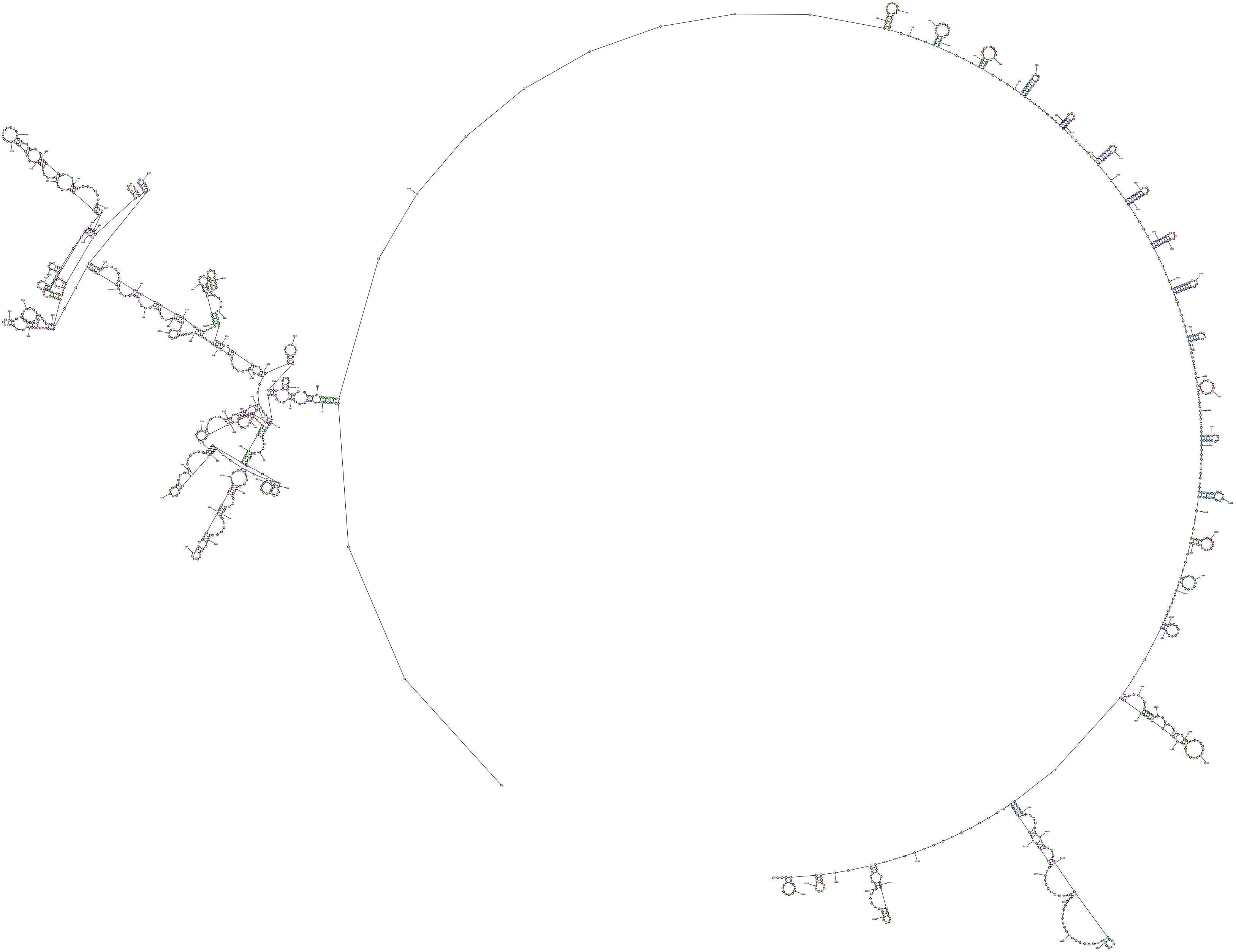

Probability >= 99%
99% > Probability >= 95%
95% > Probability >= 90%
90% > Probability >= 80%
80% > Probability >= 70%
70% > Probability >= 60%
60% > Probability >= 50%
50% > Probability

ENERGY = -288.5 CR_T_pinchaque

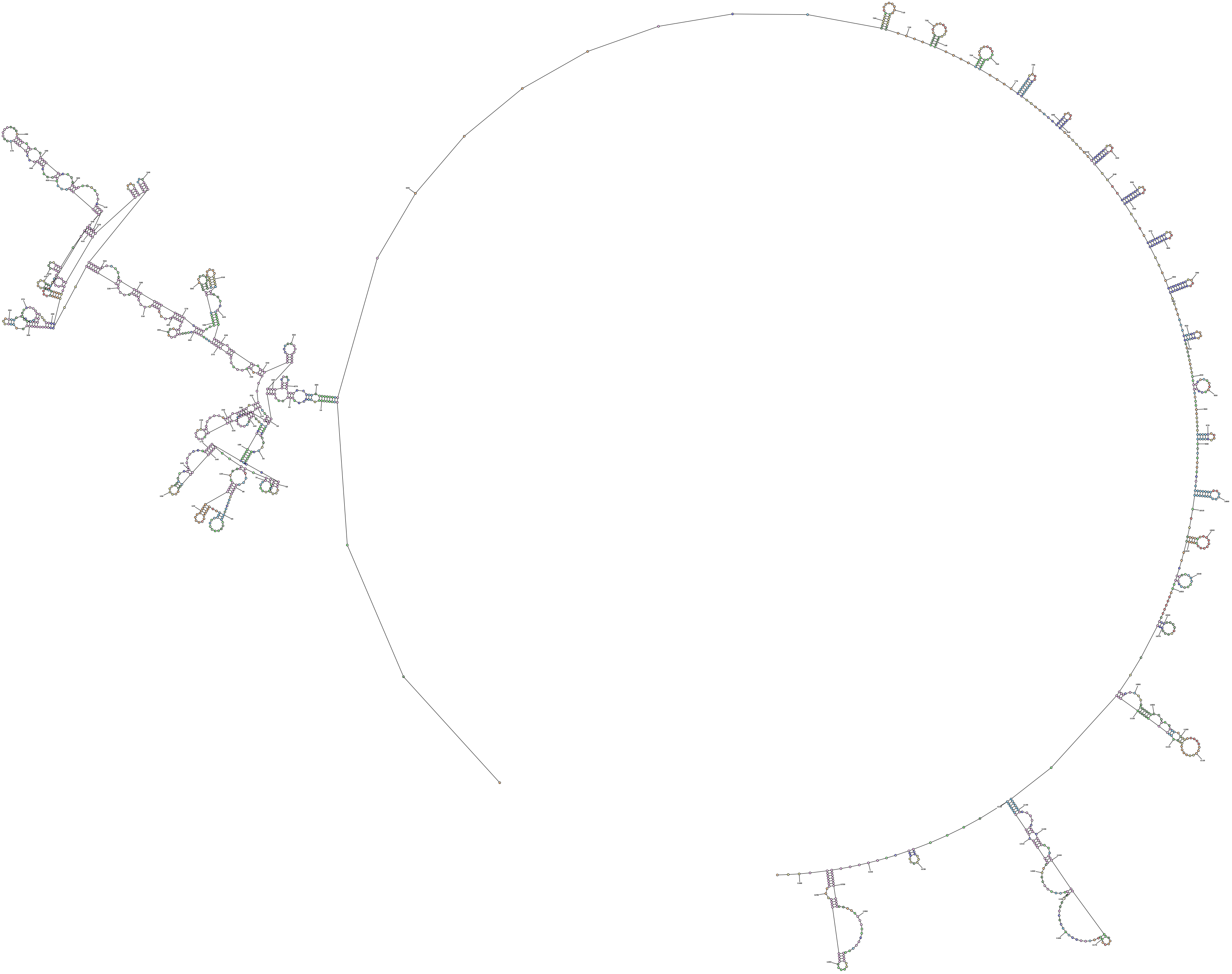

Probability >= 99%
99% > Probability >= 95%
95% > Probability >= 90%
90% > Probability >= 80%
80% > Probability >= 70%
70% > Probability >= 60%
60% > Probability >= 50%
50% > Probability

ENERGY = -288.1 CR_T_pinchaque

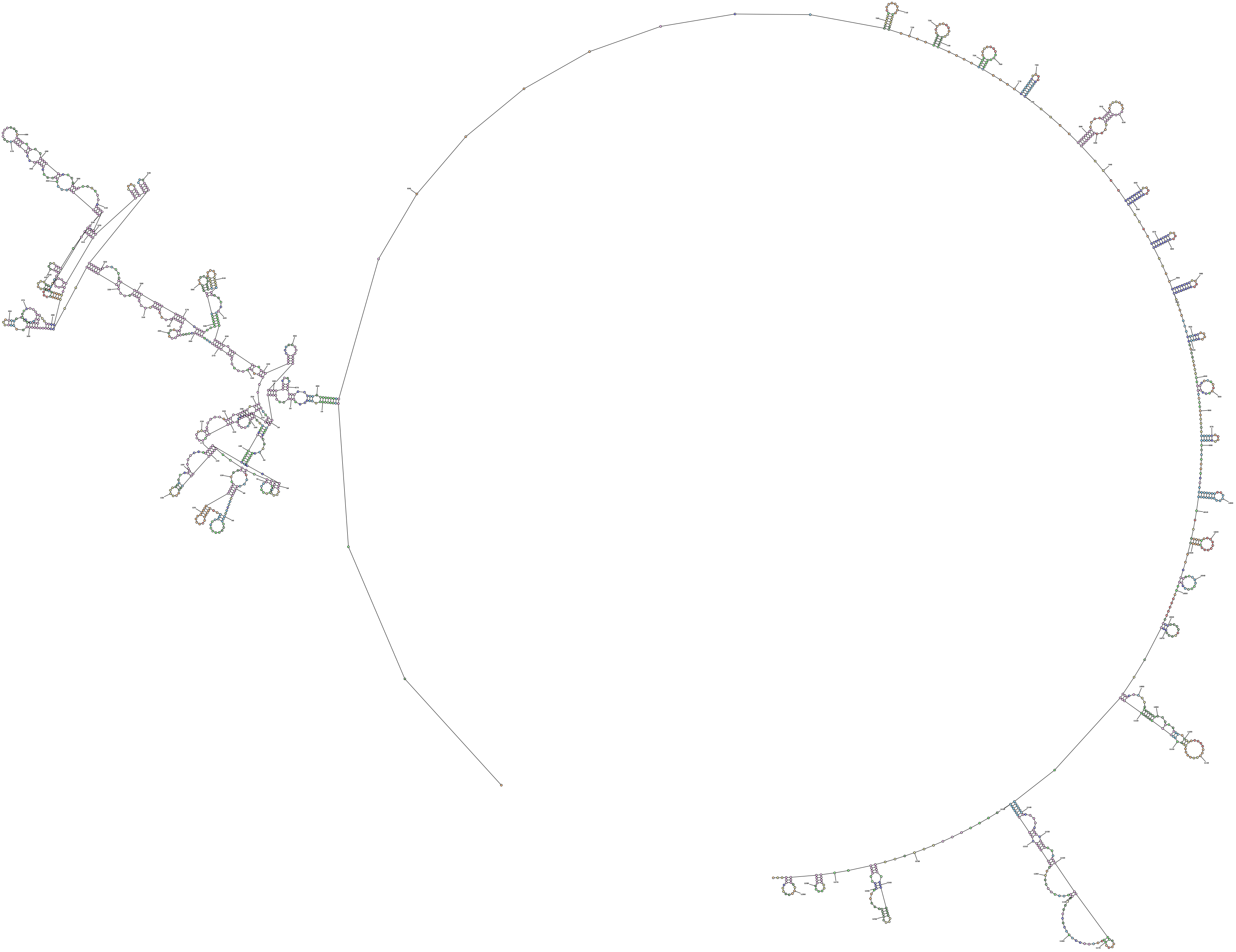

Probability >= 99%
99% > Probability >= 95%
95% > Probability >= 90%
90% > Probability >= 80%
80% > Probability >= 70%
70% > Probability >= 60%
60% > Probability >= 50%
50% > Probability

ENERGY = -287.9 CR_T_pinchaque

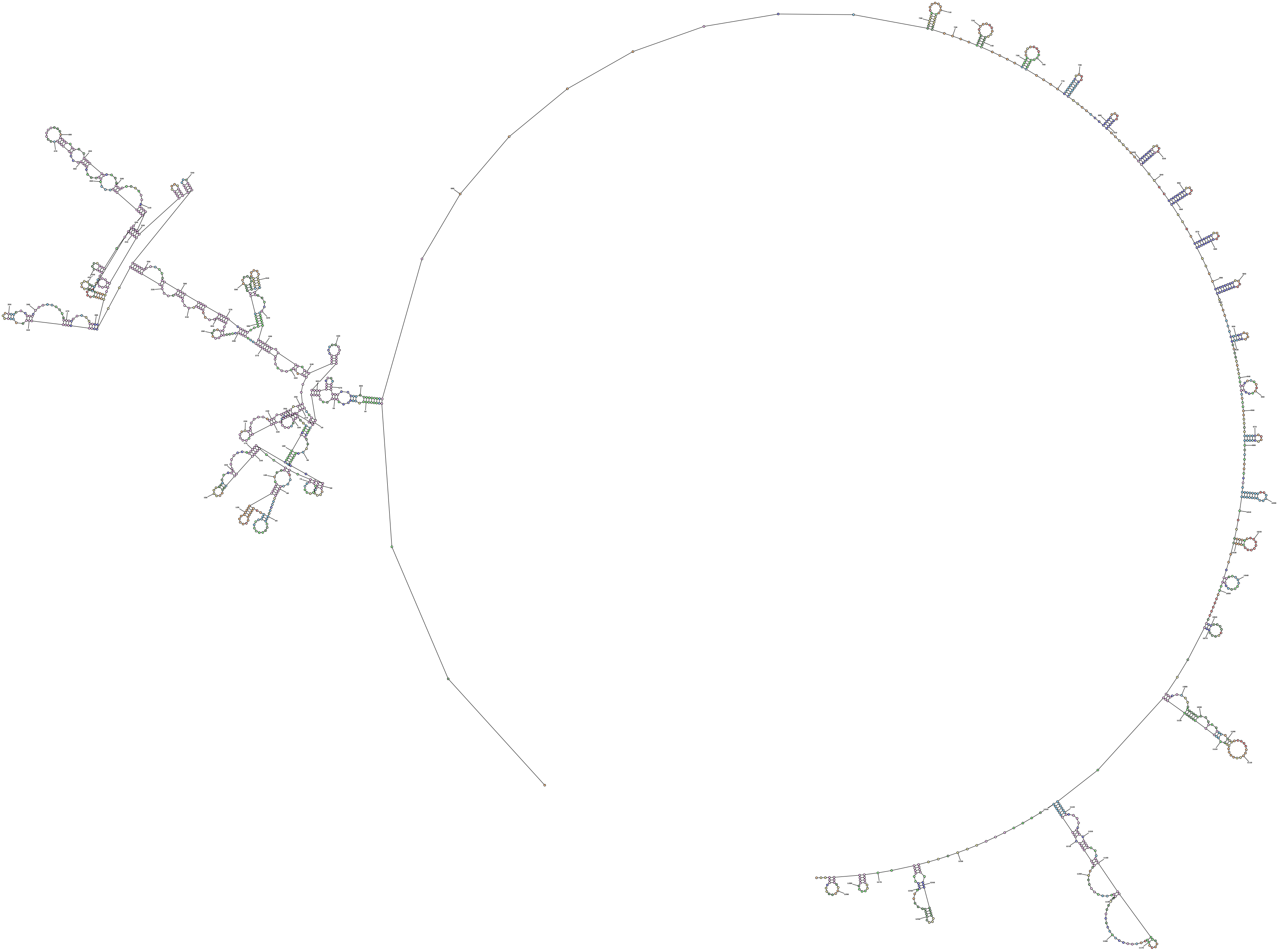

Probability >= 99%
99% > Probability >= 95%
95% > Probability >= 90%
90% > Probability >= 80%
80% > Probability >= 70%
70% > Probability >= 60%
60% > Probability >= 50%
50% > Probability

ENERGY = -287.9 CR_T_pinchaque

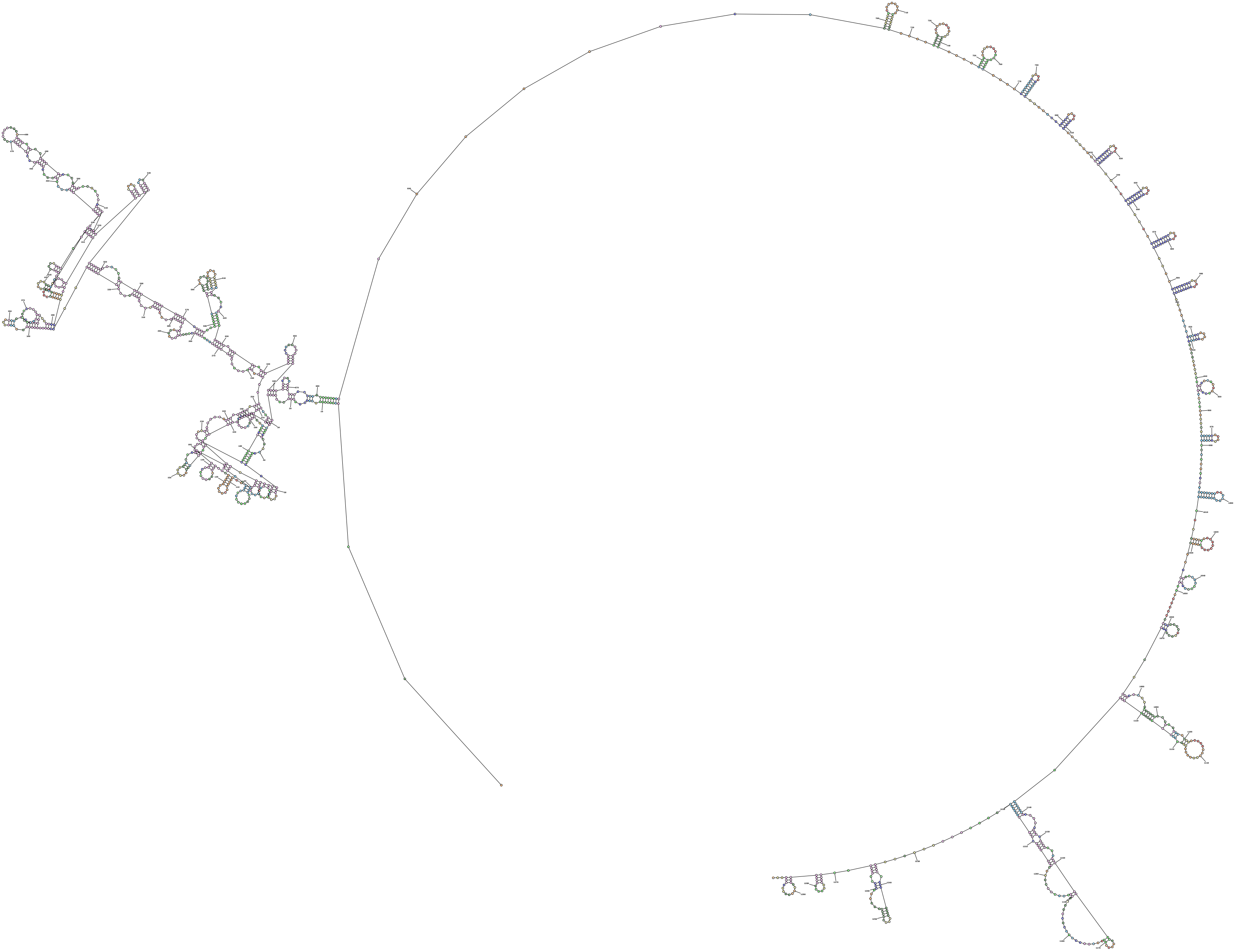

Probability >= 99%
99% > Probability >= 95%
95% > Probability >= 90%
90% > Probability >= 80%
80% > Probability >= 70%
70% > Probability >= 60%
60% > Probability >= 50%
50% > Probability

ENERGY = -287.8 CR_T_pinchaque

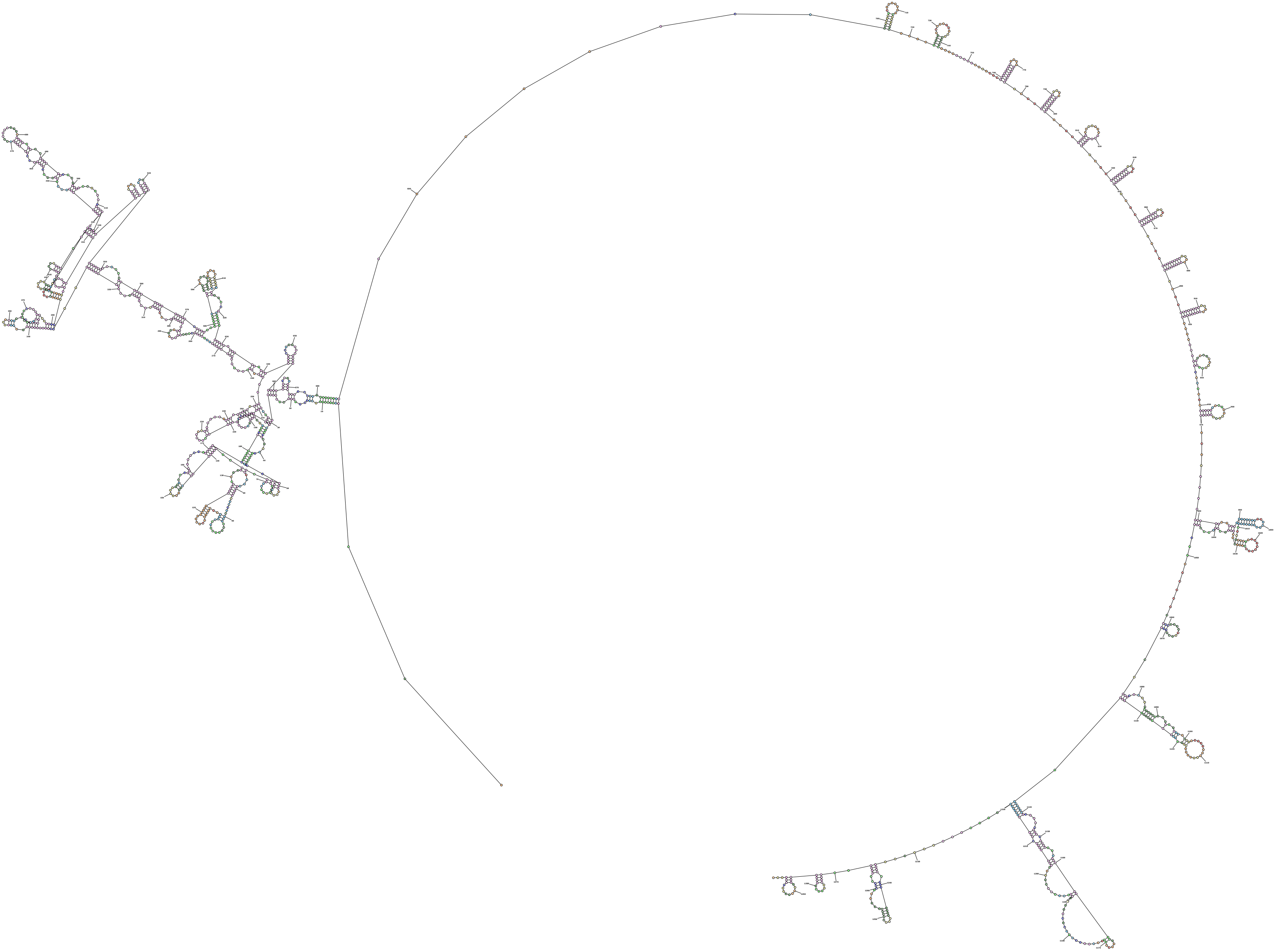

Probability >= 99%
99% > Probability >= 95%
95% > Probability >= 90%
90% > Probability >= 80%
80% > Probability >= 70%
70% > Probability >= 60%
60% > Probability >= 50%
50% > Probability

ENERGY = -287.8 CR_T_pinchaque

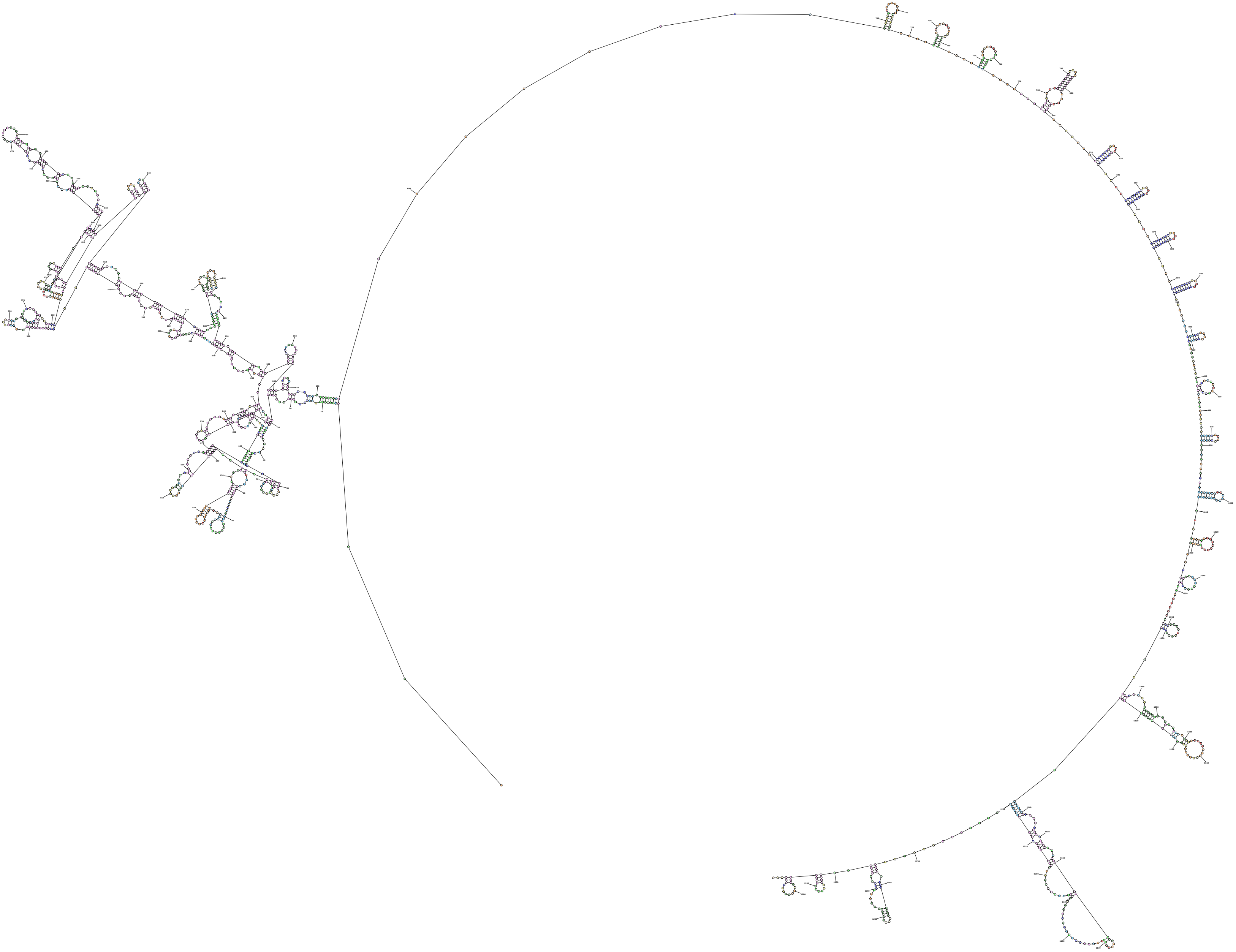

Probability >= 99%
99% > Probability >= 95%
95% > Probability >= 90%
90% > Probability >= 80%
80% > Probability >= 70%
70% > Probability >= 60%
60% > Probability >= 50%
50% > Probability

ENERGY = -287.8 CR_T_pinchaque

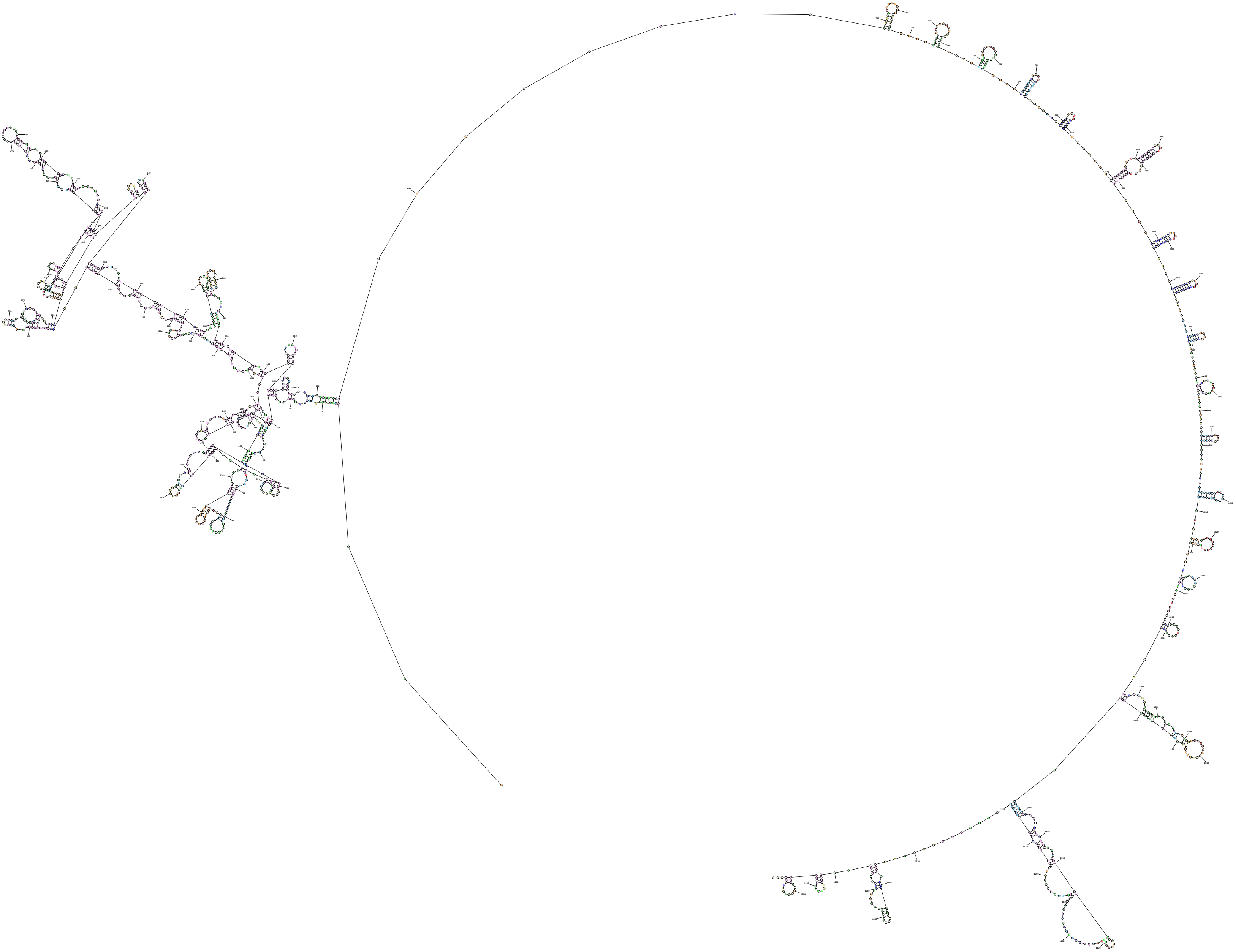

Probability >= 99%
99% > Probability >= 95%
95% > Probability >= 90%
90% > Probability >= 80%
80% > Probability >= 70%
70% > Probability >= 60%
60% > Probability >= 50%
50% > Probability

ENERGY = -287.8 CR_T_pinchaque

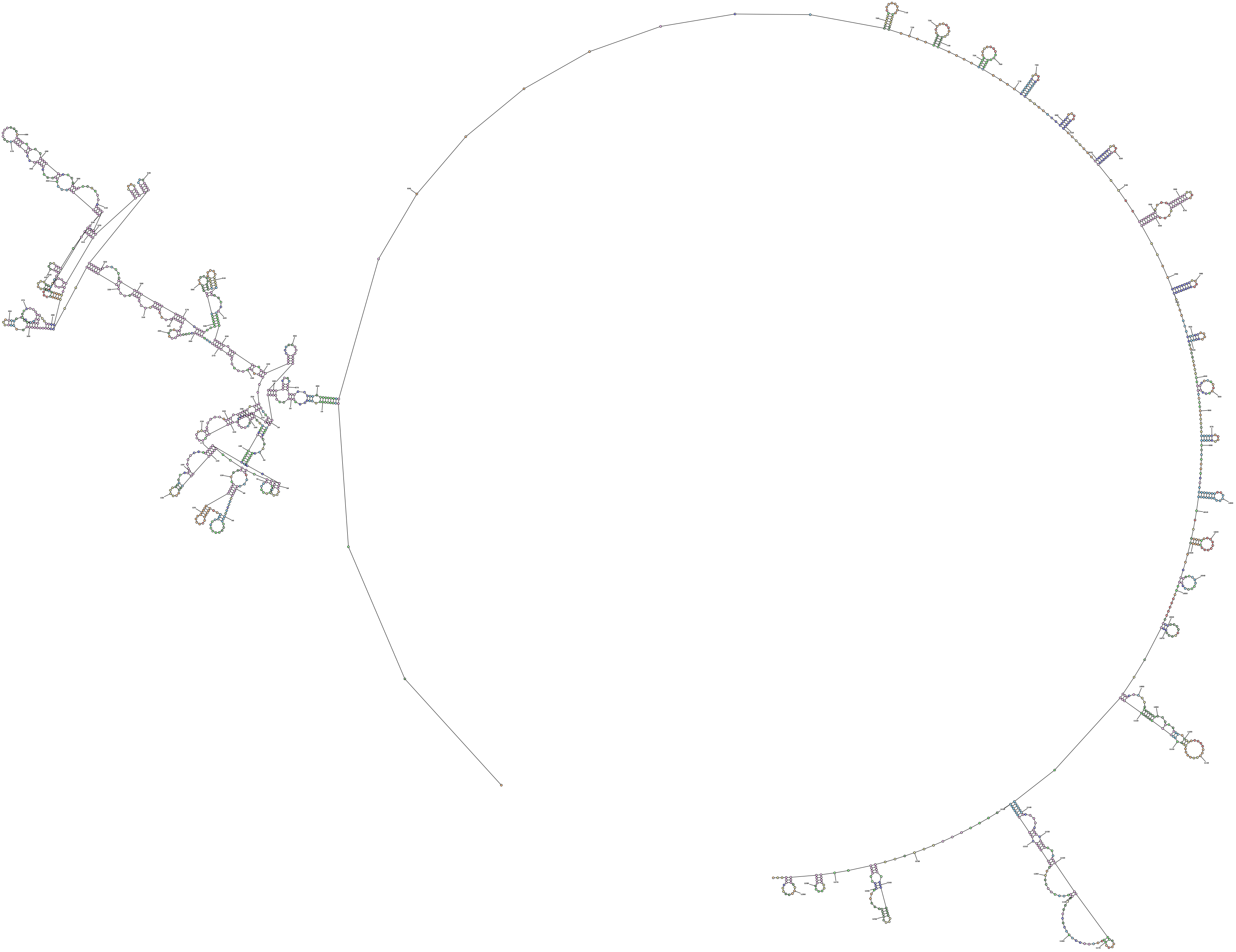

Probability >= 99%
99% > Probability >= 95%
95% > Probability >= 90%
90% > Probability >= 80%
80% > Probability >= 70%
70% > Probability >= 60%
60% > Probability >= 50%
50% > Probability

ENERGY = -287.8 CR_T_pinchaque

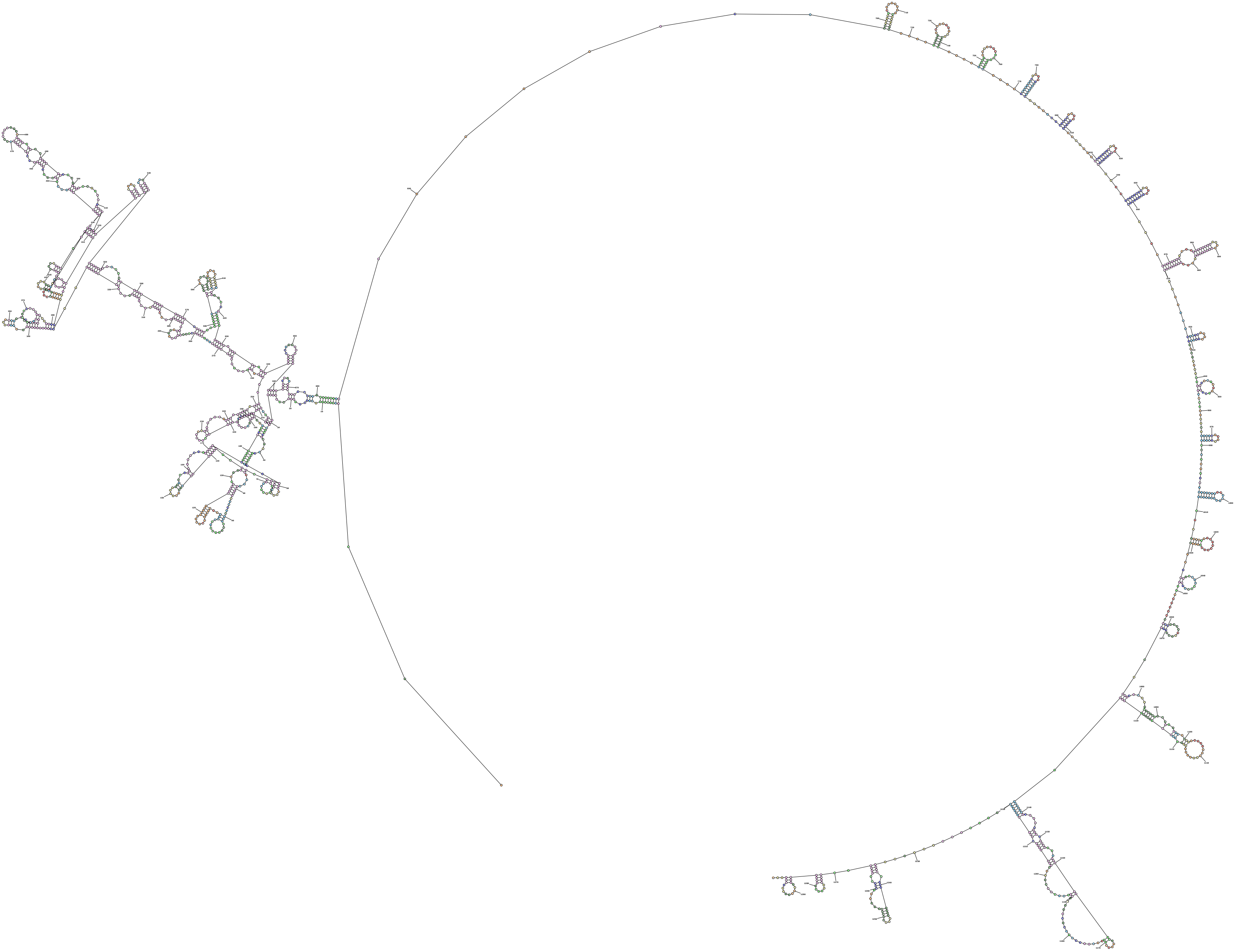

Probability >= 99%
99% > Probability >= 95%
95% > Probability >= 90%
90% > Probability >= 80%
80% > Probability >= 70%
70% > Probability >= 60%
60% > Probability >= 50%
50% > Probability

ENERGY = -287.8 CR_T_pinchaque

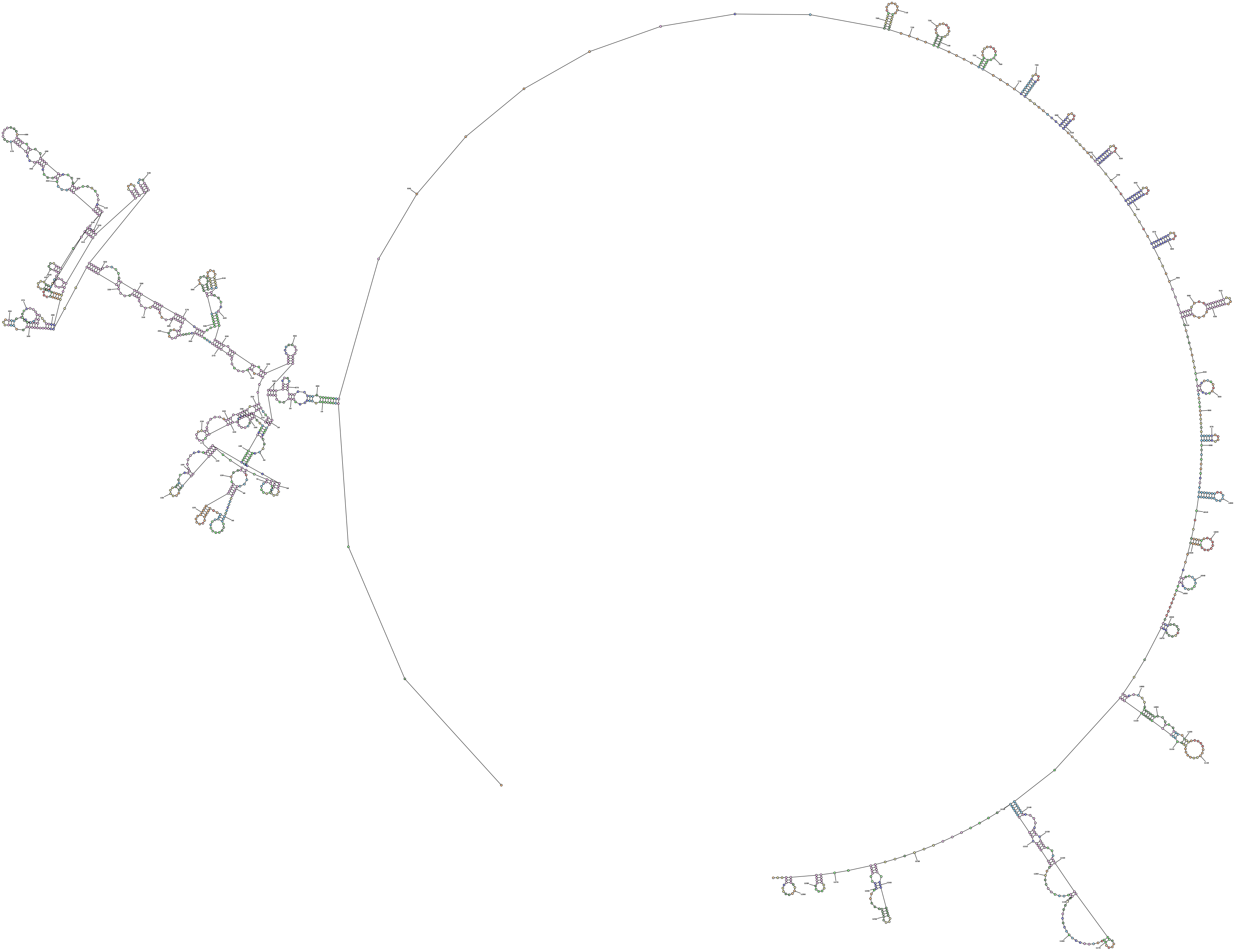

Probability >= 99%
99% > Probability >= 95%
95% > Probability >= 90%
90% > Probability >= 80%
80% > Probability >= 70%
70% > Probability >= 60%
60% > Probability >= 50%
50% > Probability

ENERGY = -287.8 CR_T_pinchaque

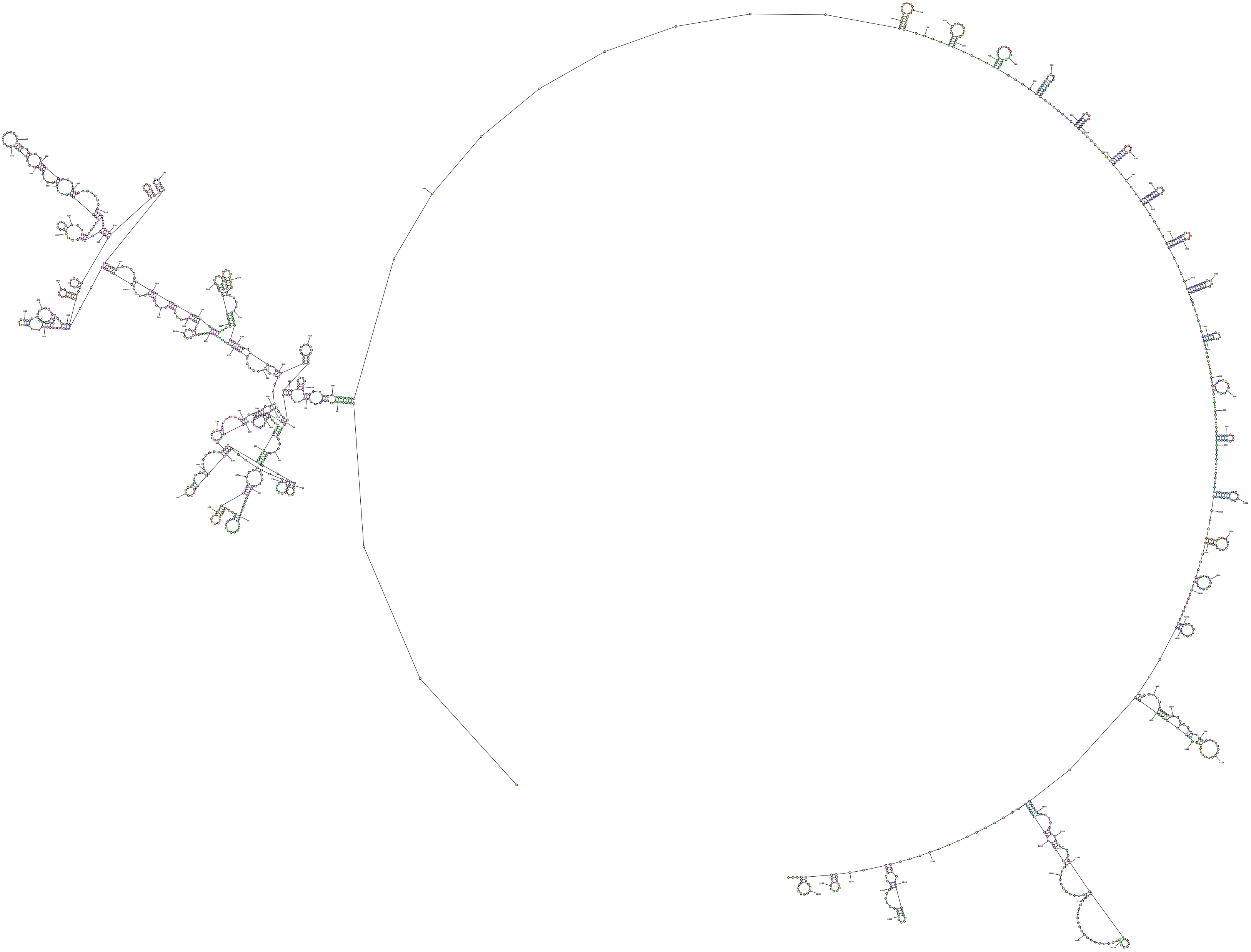

Probability >= 99%
99% > Probability >= 95%
95% > Probability >= 90%
90% > Probability >= 80%
80% > Probability >= 70%
70% > Probability >= 60%
60% > Probability >= 50%
50% > Probability

ENERGY = -287.8 CR_T_pinchaque

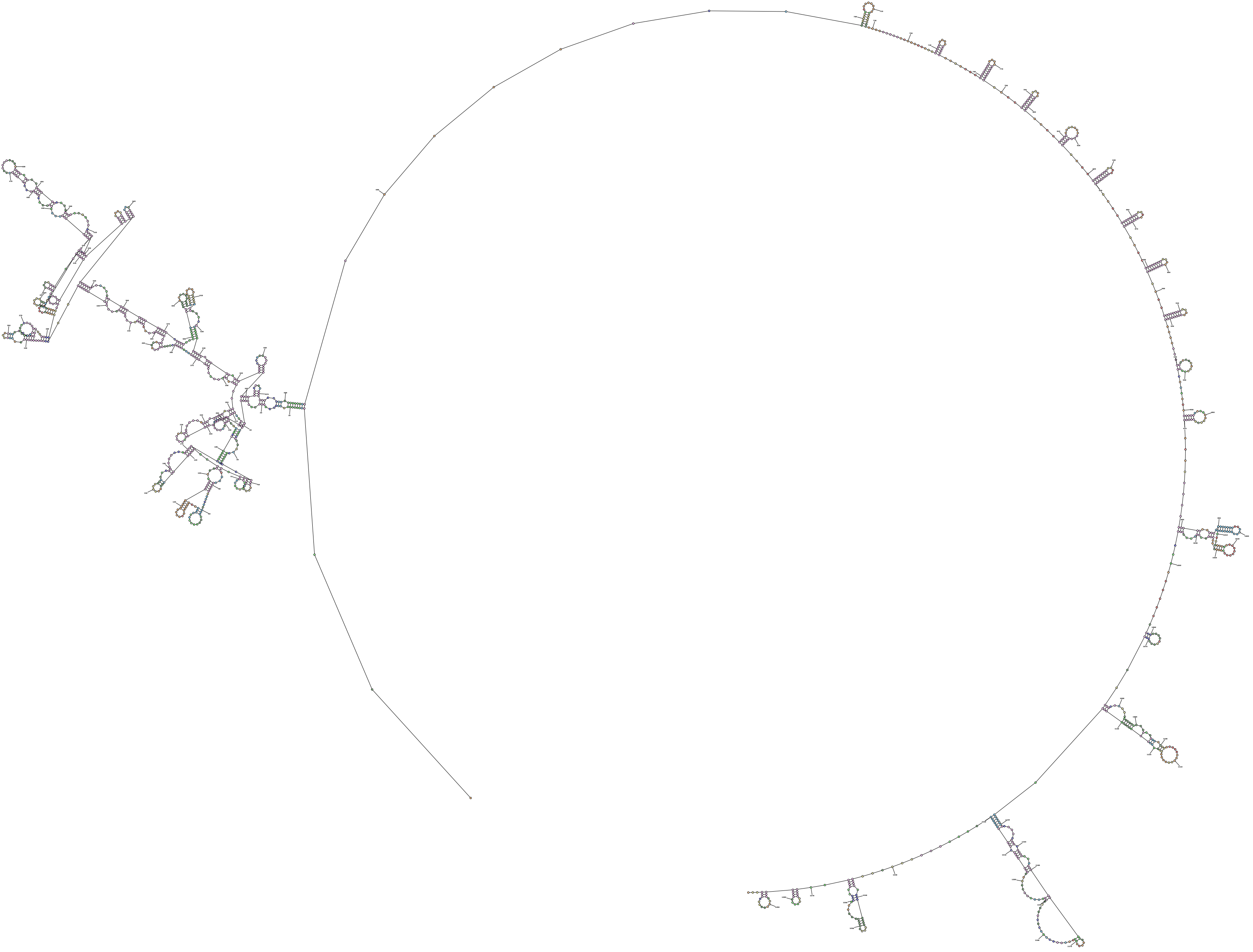

Probability >= 99%
99% > Probability >= 95%
95% > Probability >= 90%
90% > Probability >= 80%
80% > Probability >= 70%
70% > Probability >= 60%
60% > Probability >= 50%
50% > Probability

ENERGY = -287.7 CR_T_pinchaque

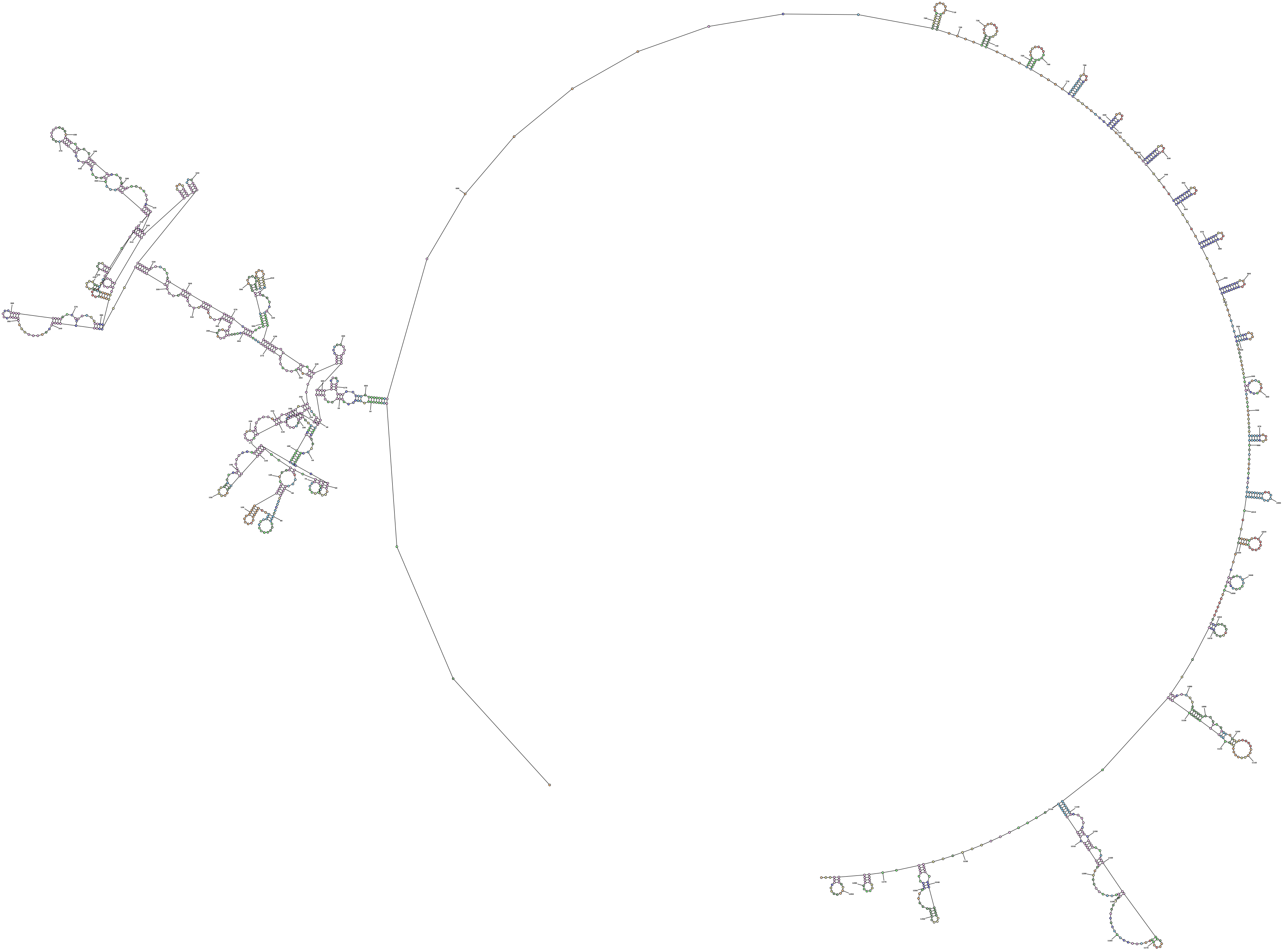

Probability >= 99%
99% > Probability >= 95%
95% > Probability >= 90%
90% > Probability >= 80%
80% > Probability >= 70%
70% > Probability >= 60%
60% > Probability >= 50%
50% > Probability

ENERGY = -287.7 CR_T_pinchaque

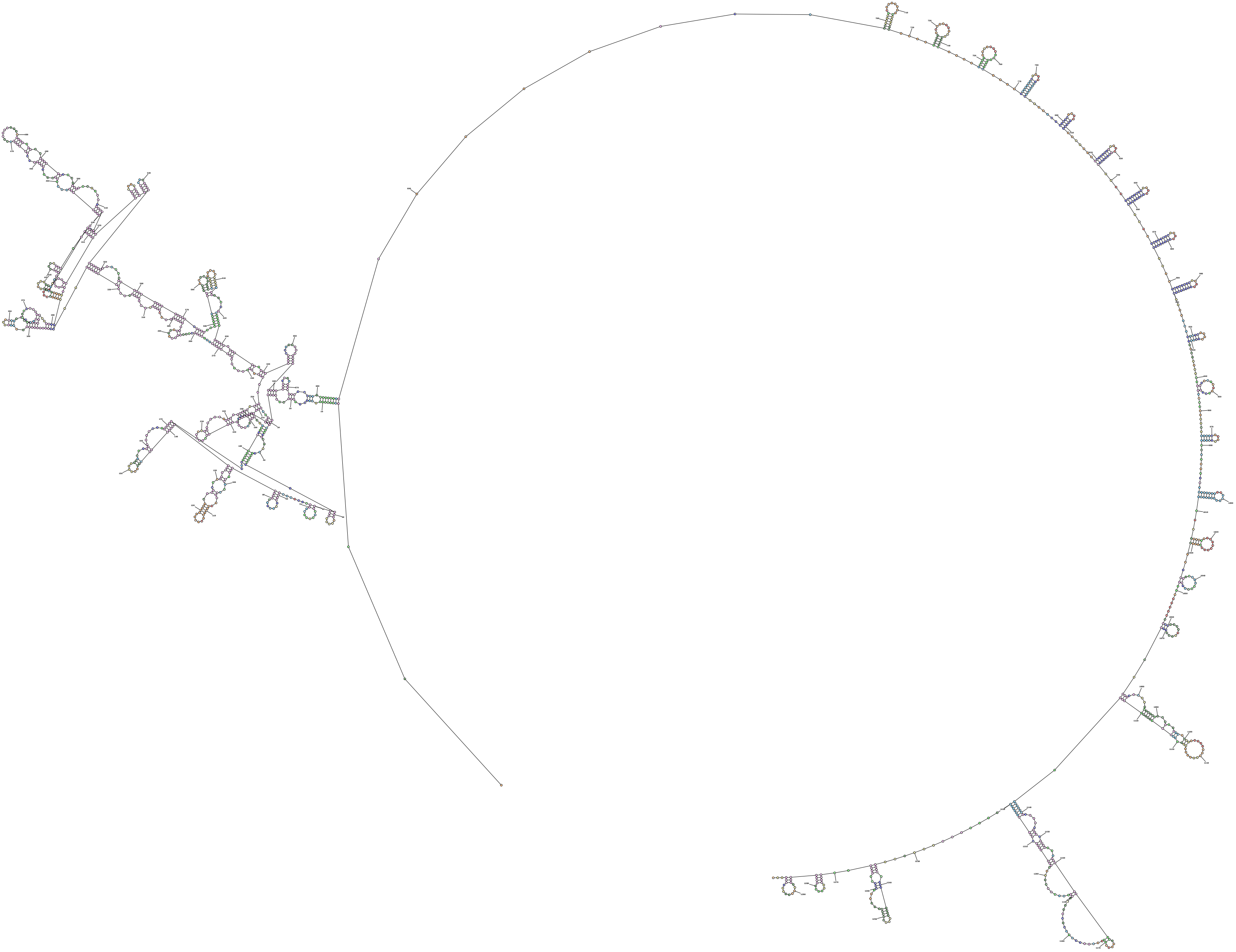

Probability >= 99%
99% > Probability >= 95%
95% > Probability >= 90%
90% > Probability >= 80%
80% > Probability >= 70%
70% > Probability >= 60%
60% > Probability >= 50%
50% > Probability

ENERGY = -287.6 CR_T_pinchaque

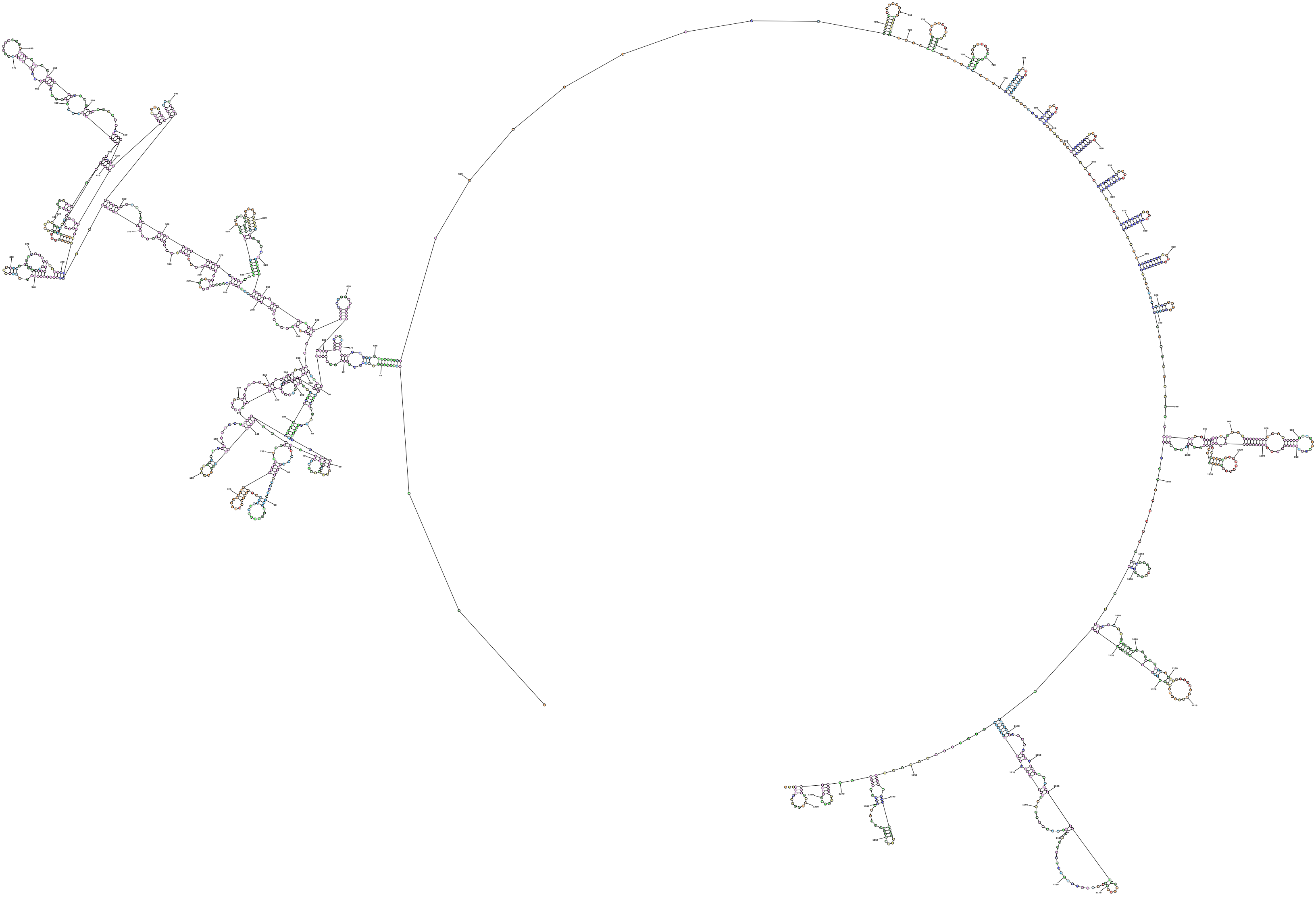

Probability >= 99%
99% > Probability >= 95%
95% > Probability >= 90%
90% > Probability >= 80%
80% > Probability >= 70%
70% > Probability >= 60%
60% > Probability >= 50%
50% > Probability

ENERGY = -287.6 CR_T_pinchaque

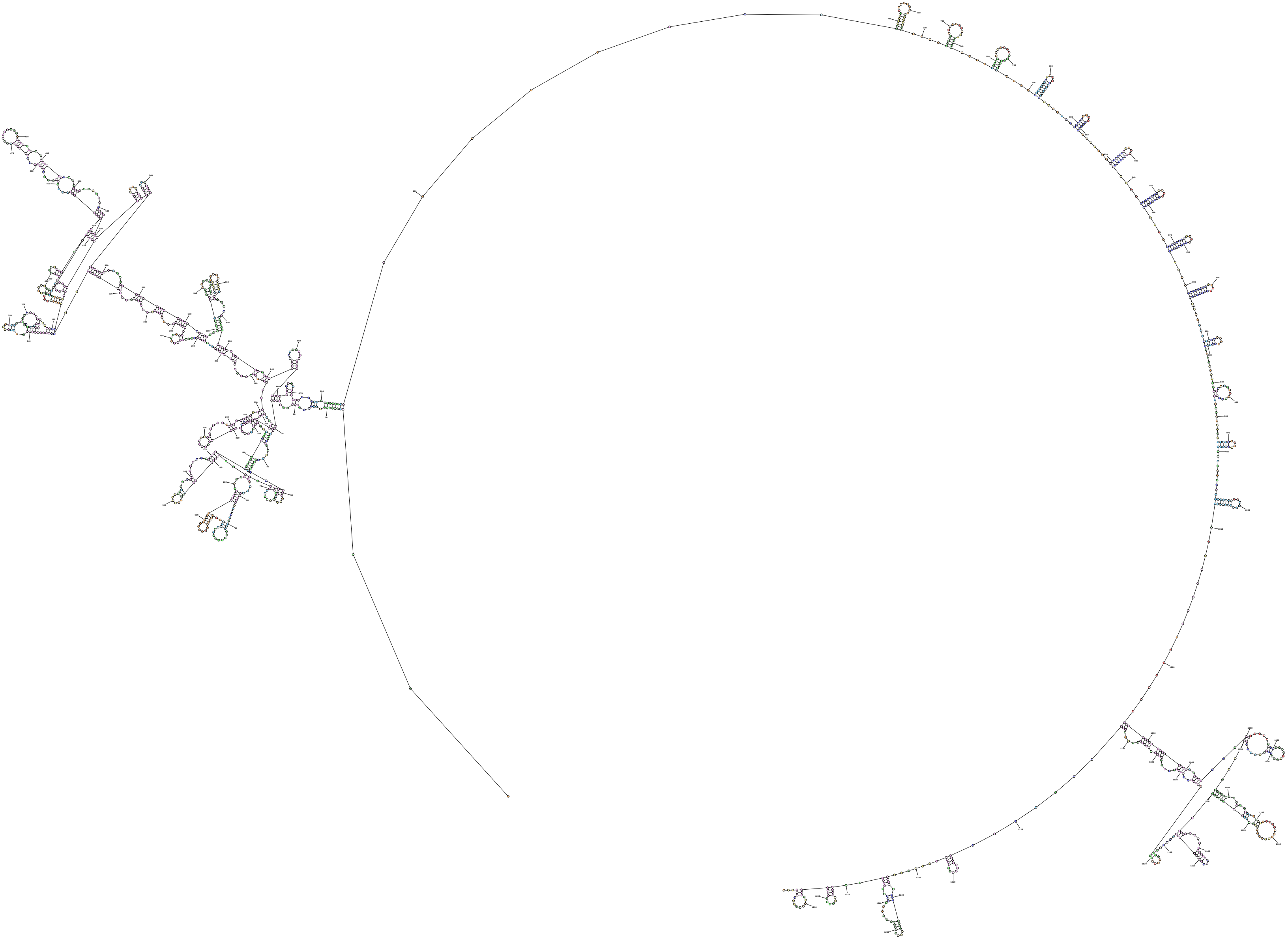

Probability >= 99%
99% > Probability >= 95%
95% > Probability >= 90%
90% > Probability >= 80%
80% > Probability >= 70%
70% > Probability >= 60%
60% > Probability >= 50%
50% > Probability

ENERGY = -287.6 CR_T_pinchaque
