# Supplementary material for: The mitochondrial genome of the mountain wooly tapir, Tapirus pinchaque and a formal test of the effect of altitude on the adaptive evolution of mitochondrial protein coding genes in odd-toed ungulates
Source: BMC Genomics. 2023 Sep 6;24:527. doi: 10.1186/s12864-023-09596-8 (PMC10481570; doi:10.1186/s12864-023-09596-8)
Supplement: Supplementary file 2 — Additional file 2: Supplementary Figure S1. Secondary structure predictions of the Control Region. [file 12864_2023_9596_MOESM2_ESM.zip › 12864_2023_9596_MOESM4_ESM.pdf]

Conservation Genetics

The mitochondrial genome of the mountain wooly tapir, *Tapirus pinchaque* and a formal test of the effect of altitude on the adaptive evolution of mitochondrial protein coding genes in odd-toed ungulates

Edgar G. Gutiérrez[1], Jorge Ortega[1], and J. Antonio Baeza[2,3,4] *

1Laboratorio de Bioconservación y Manejo, Posgrado en Ciencias Químicobiológicas, Departamento de Zoología, Escuela Nacional de Ciencias Biológicas, Instituto Politécnico Nacional, Prolongación Carpio y Plan de Ayala s/n, Col. Santo Tomás, C.P. 11340, Ciudad de México, México.
2 Department of Biological Sciences, 132 Long Hall, Clemson University, Clemson, SC 29634, USA.
3 Smithsonian Marine Station at Fort Pierce, 701 Seaway Drive, Fort Pierce, FL 34949, USA.
4 Departamento de Biología Marina, Facultad de Ciencias del Mar, Universidad Católica del Norte, Larrondo 1281, Coquimbo, Chile.

*Correspondence author: Email: baeza.antonio@gmail.com

Supplementary Figure S1. Secondary structure predictions of the Control Region

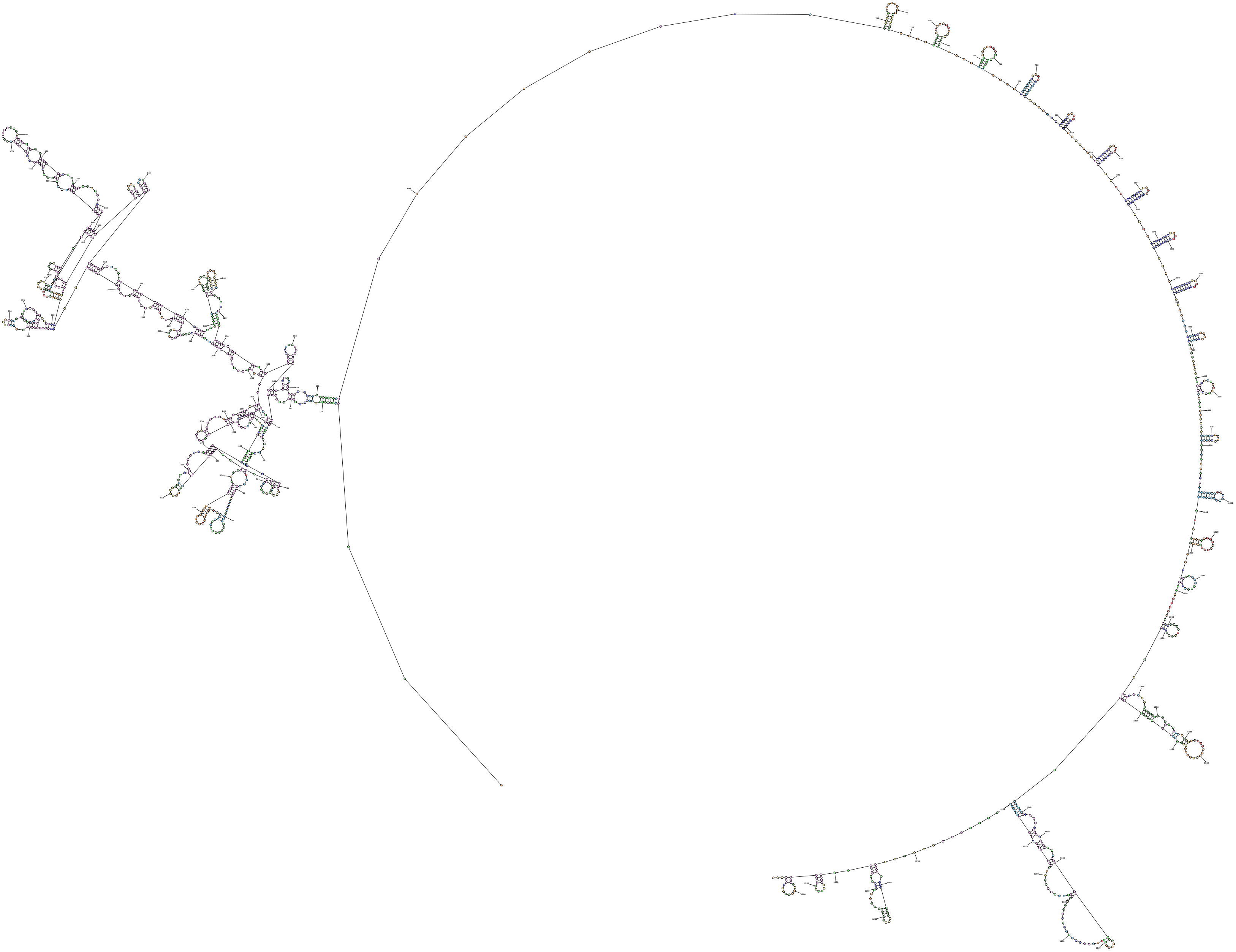

Supplementary Figure S1-1. Secondary structure predictions of the Control Region

Probability >= 99%
99% > Probability >= 95%
95% > Probability >= 90%
90% > Probability >= 80%
80% > Probability >= 70%
70% > Probability >= 60%
60% > Probability >= 50%
50% > Probability

ENERGY = -288.9 CR_T_pinchaque

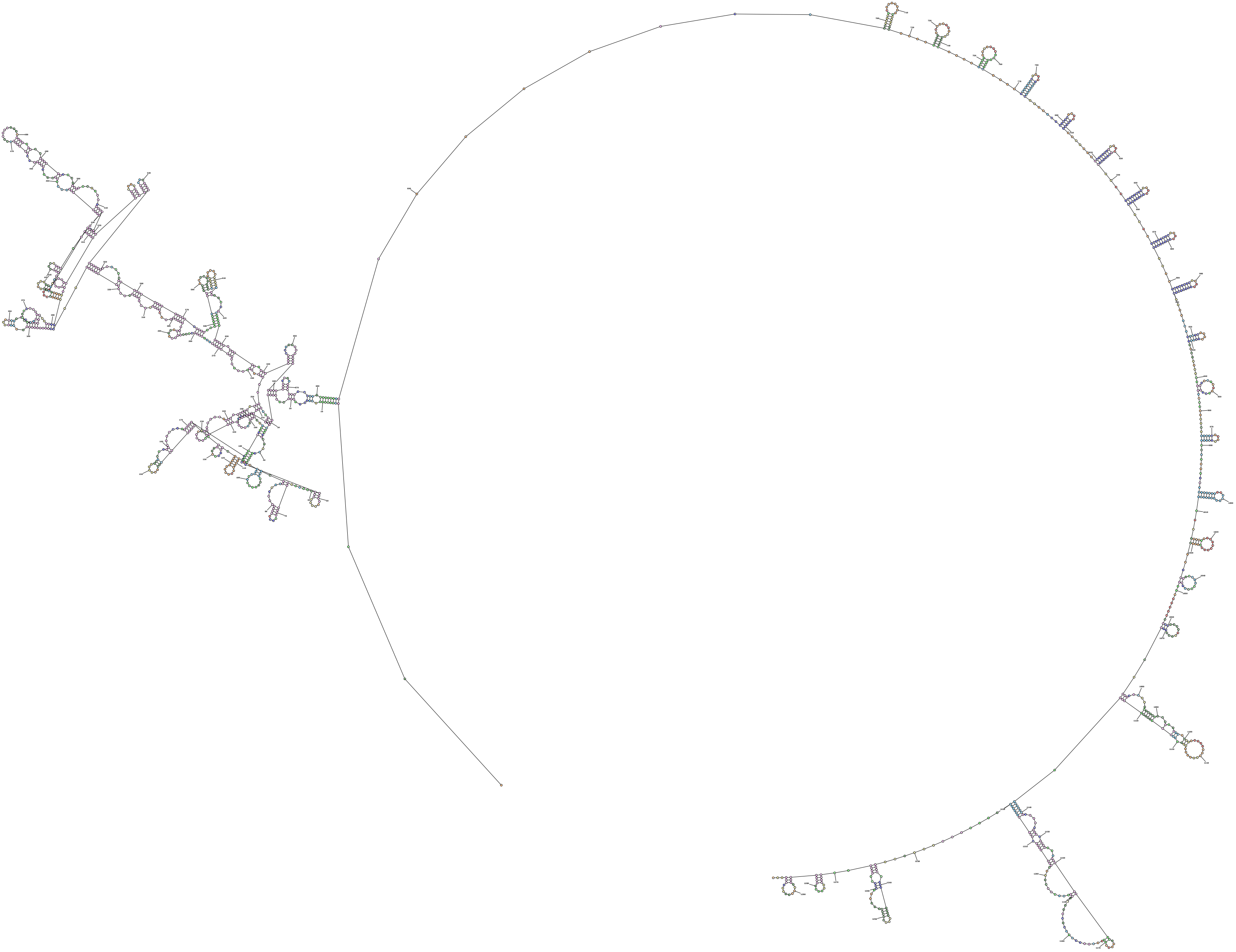

Supplementary Figure S1-2. Secondary structure predictions of the Control Region

Probability >= 99%
99% > Probability >= 95%
95% > Probability >= 90%
90% > Probability >= 80%
80% > Probability >= 70%
70% > Probability >= 60%
60% > Probability >= 50%
50% > Probability

ENERGY = -288.9 CR_T_pinchaque

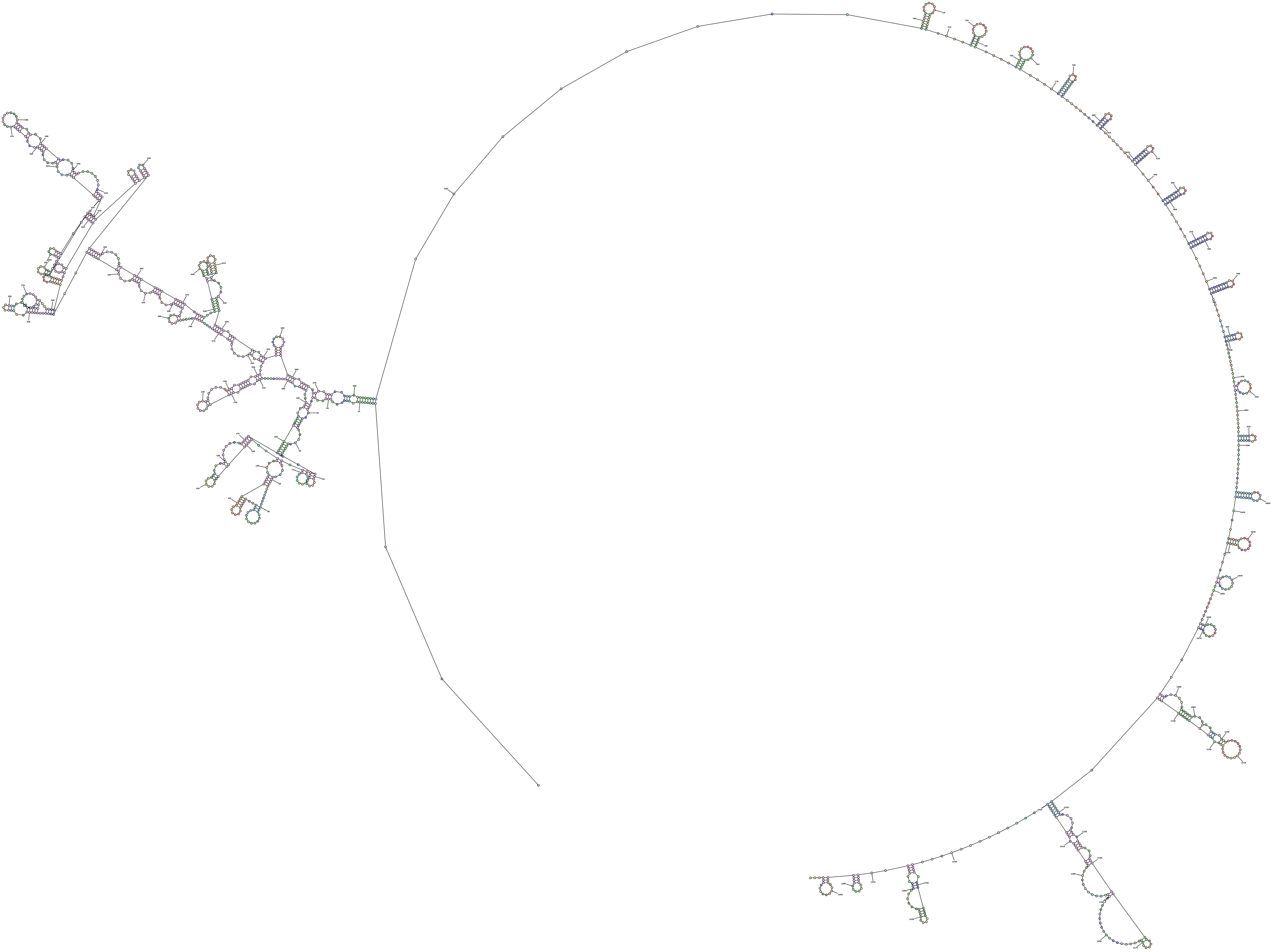

Supplementary Figure S1-3. Secondary structure predictions of the Control Region

Probability >= 99%
99% > Probability >= 95%
95% > Probability >= 90%
90% > Probability >= 80%
80% > Probability >= 70%
70% > Probability >= 60%
60% > Probability >= 50%
50% > Probability

ENERGY = -288.6 CR_T_pinchaque

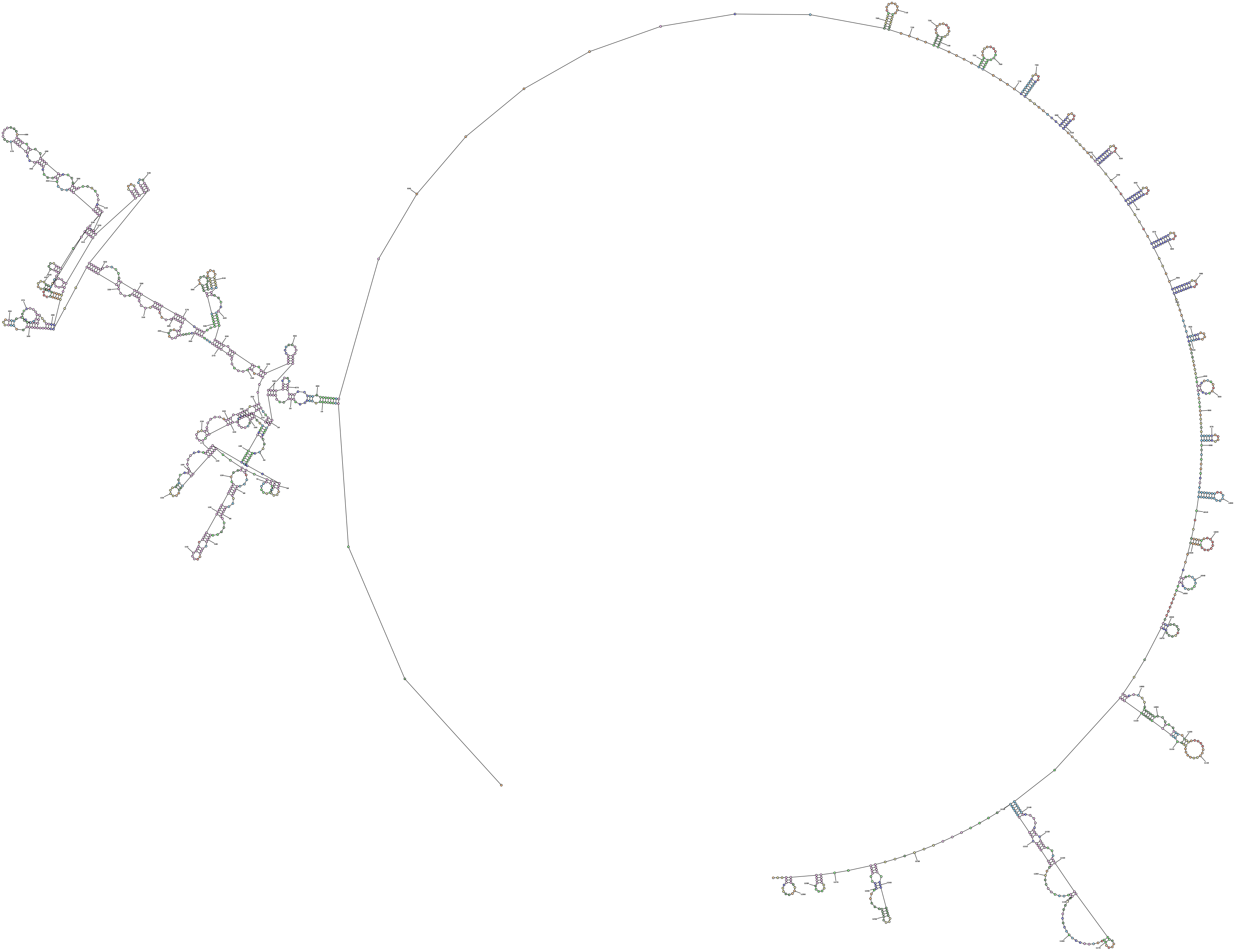

Supplementary Figure S1-4. Secondary structure predictions of the Control Region

Probability >= 99%
99% > Probability >= 95%
95% > Probability >= 90%
90% > Probability >= 80%
80% > Probability >= 70%
70% > Probability >= 60%
60% > Probability >= 50%
50% > Probability

ENERGY = -288.5 CR_T_pinchaque

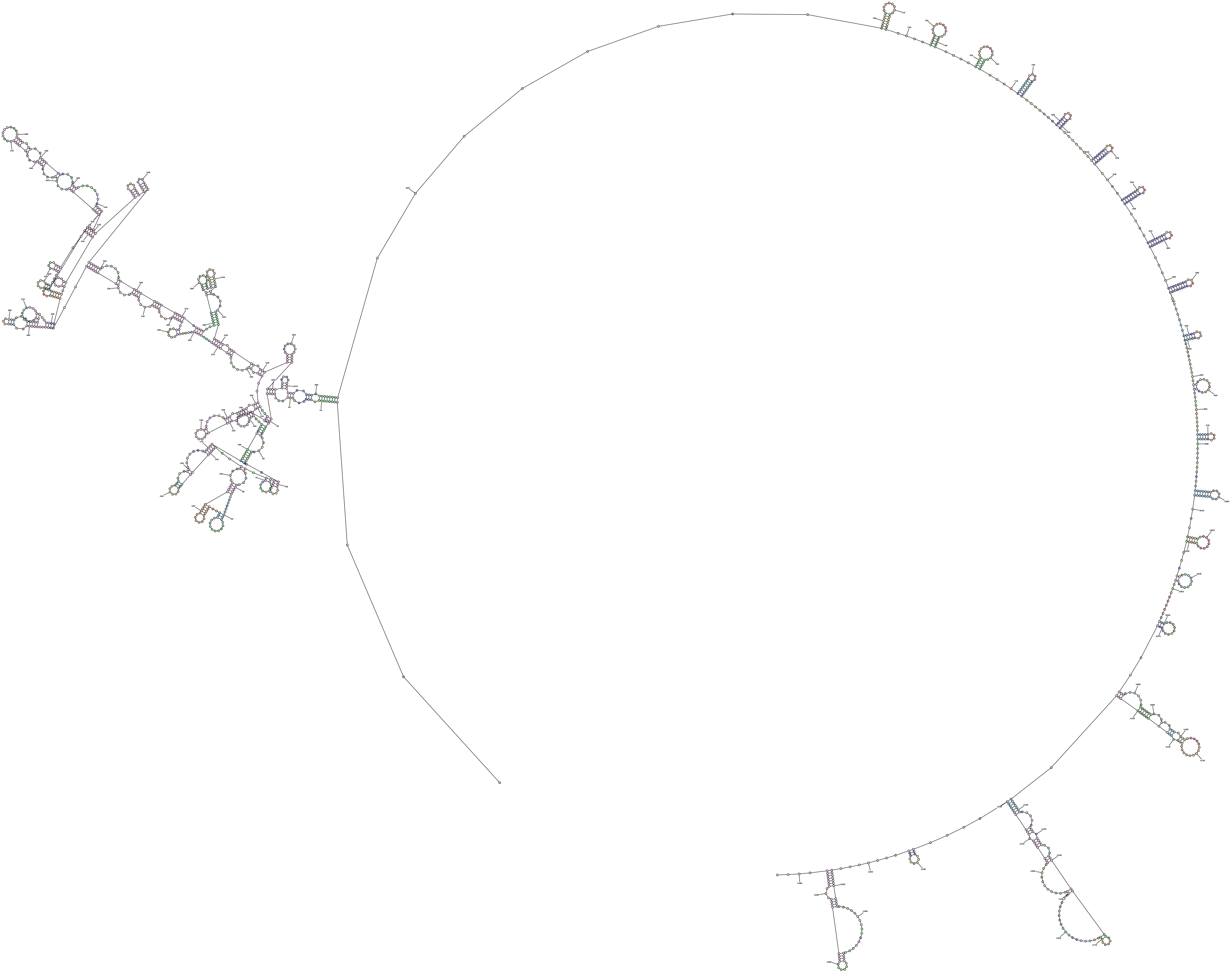

Supplementary Figure S1-5. Secondary structure predictions of the Control Region

Probability >= 99%
99% > Probability >= 95%
95% > Probability >= 90%
90% > Probability >= 80%
80% > Probability >= 70%
70% > Probability >= 60%
60% > Probability >= 50%
50% > Probability

ENERGY = -288.1 CR_T_pinchaque

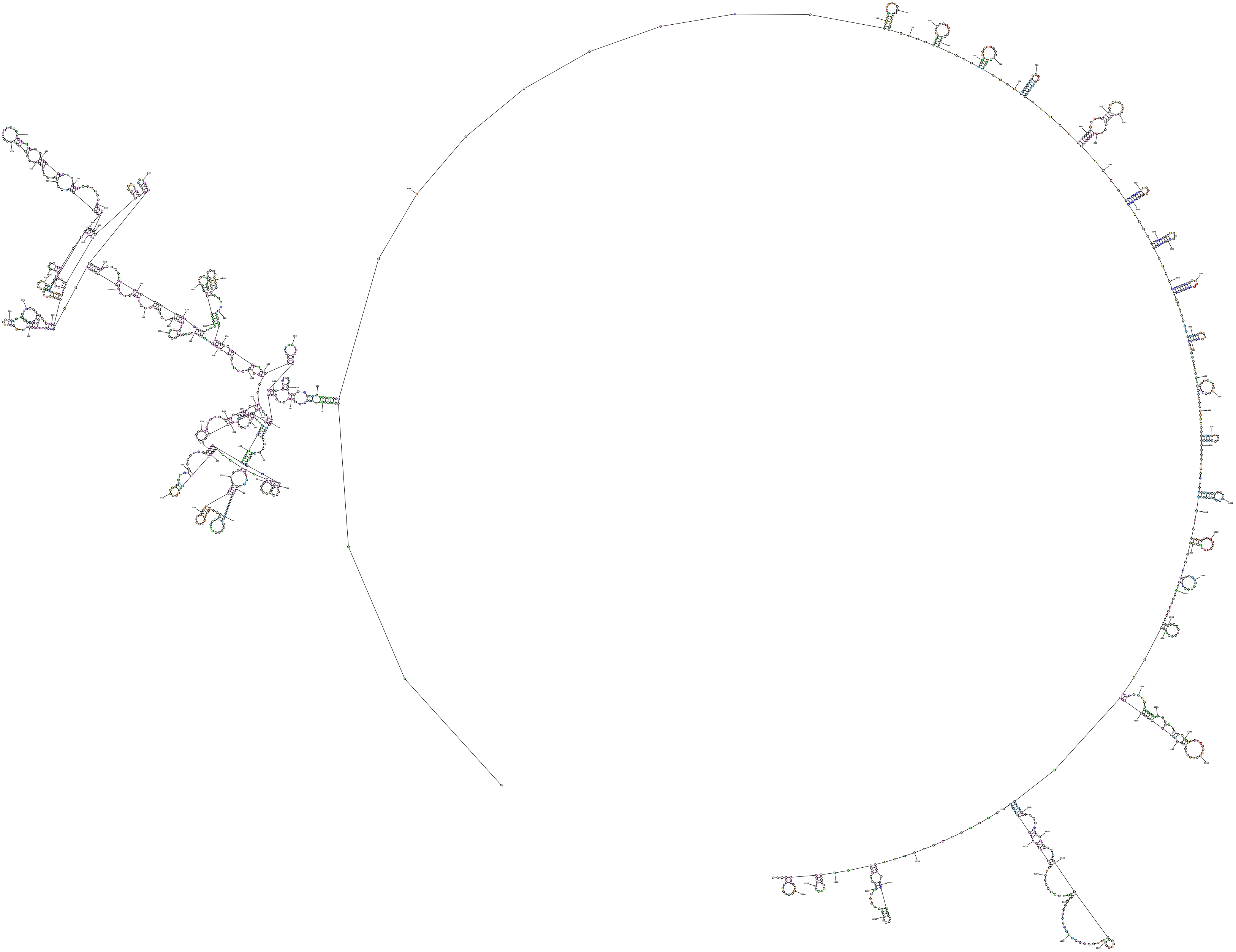

Supplementary Figure S1-6. Secondary structure predictions of the Control Region

Probability >= 99%
99% > Probability >= 95%
95% > Probability >= 90%
90% > Probability >= 80%
80% > Probability >= 70%
70% > Probability >= 60%
60% > Probability >= 50%
50% > Probability

ENERGY = -287.9 CR_T_pinchaque

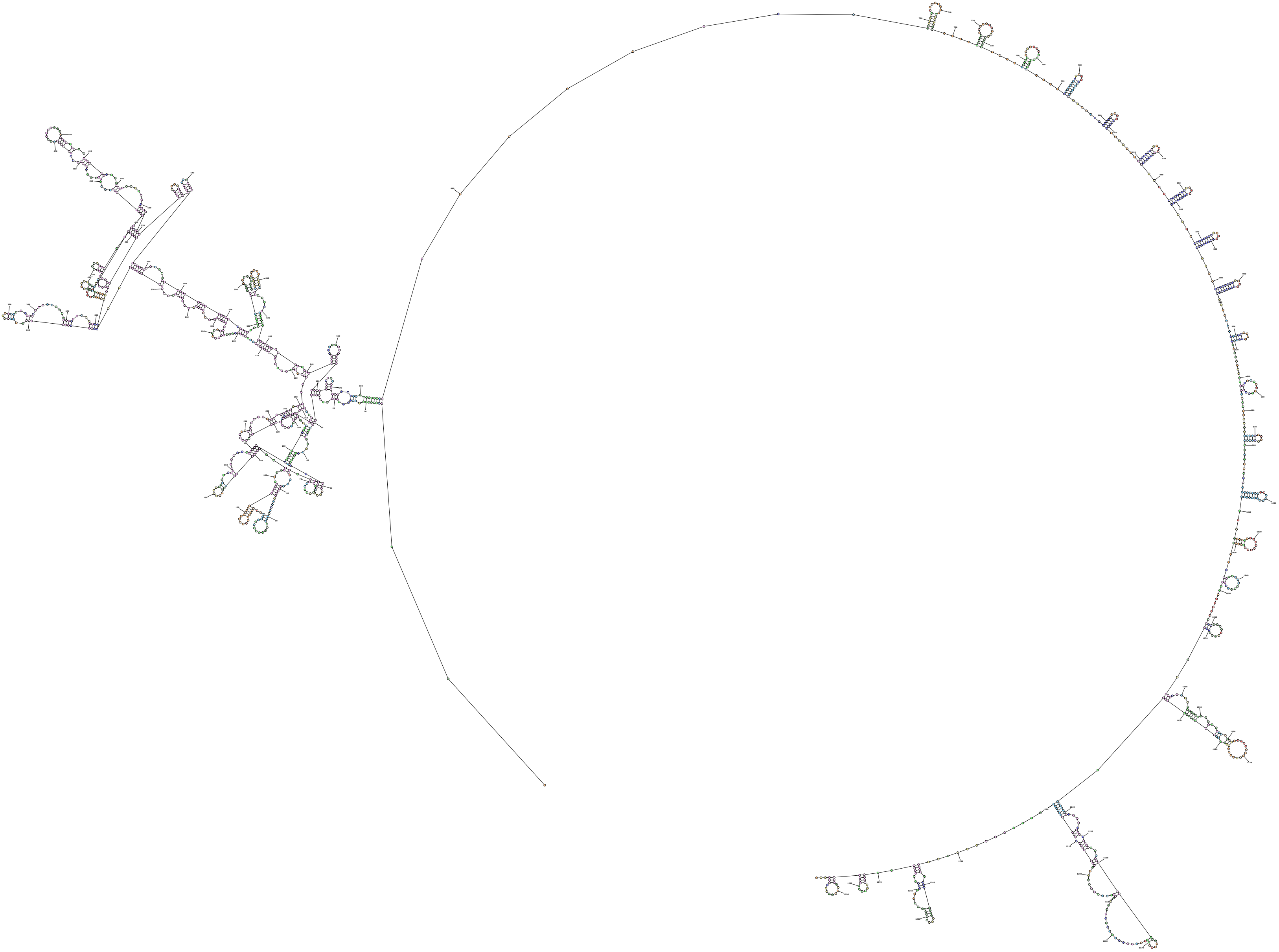

Supplementary Figure S1-7. Secondary structure predictions of the Control Region

Probability >= 99%
99% > Probability >= 95%
95% > Probability >= 90%
90% > Probability >= 80%
80% > Probability >= 70%
70% > Probability >= 60%
60% > Probability >= 50%
50% > Probability

ENERGY = -287.9 CR_T_pinchaque

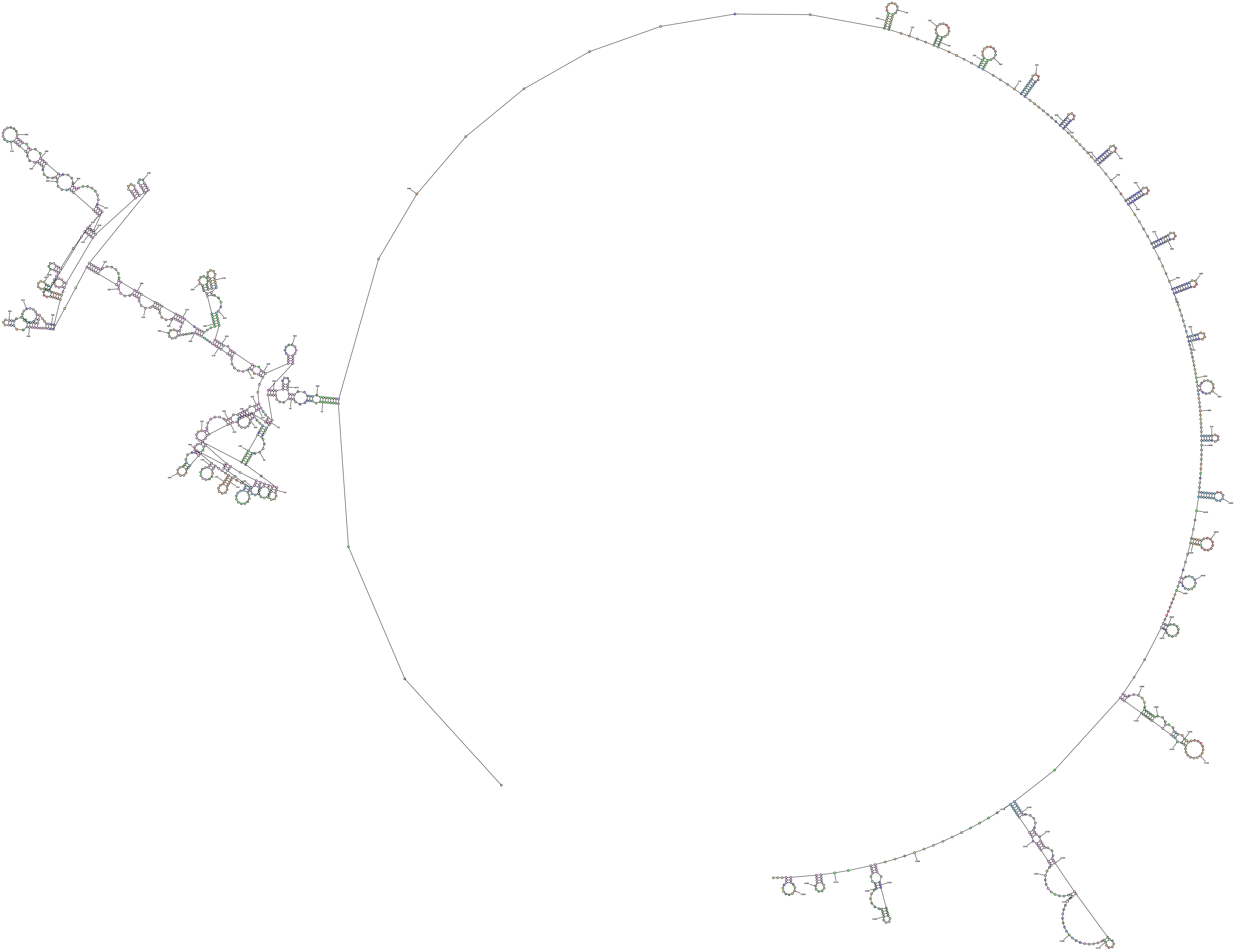

Supplementary Figure S1-8. Secondary structure predictions of the Control Region

Probability >= 99%
99% > Probability >= 95%
95% > Probability >= 90%
90% > Probability >= 80%
80% > Probability >= 70%
70% > Probability >= 60%
60% > Probability >= 50%
50% > Probability

ENERGY = -287.8 CR_T_pinchaque

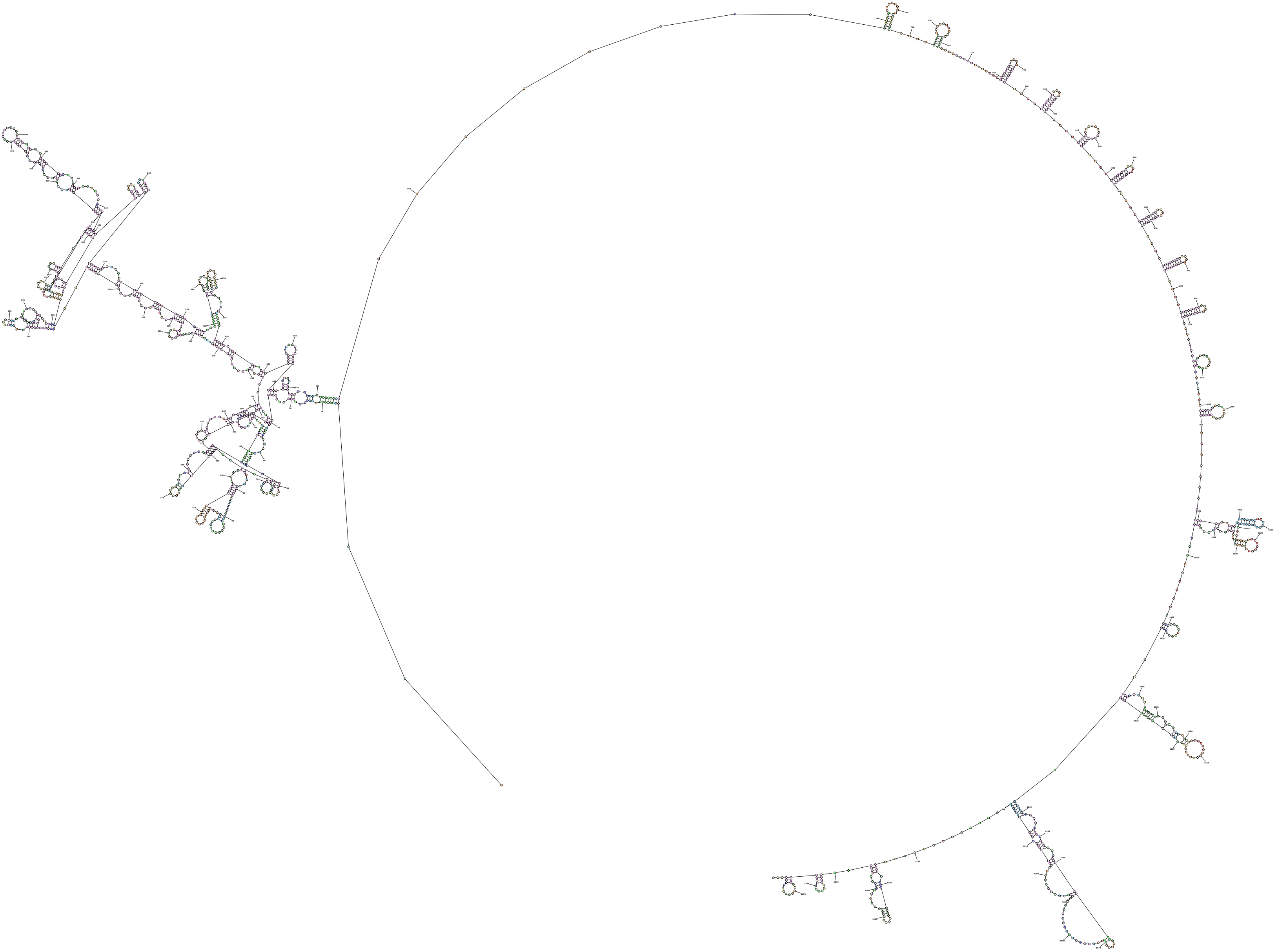

Supplementary Figure S1-9. Secondary structure predictions of the Control Region

Probability >= 99%
99% > Probability >= 95%
95% > Probability >= 90%
90% > Probability >= 80%
80% > Probability >= 70%
70% > Probability >= 60%
60% > Probability >= 50%
50% > Probability

ENERGY = -287.8 CR_T_pinchaque

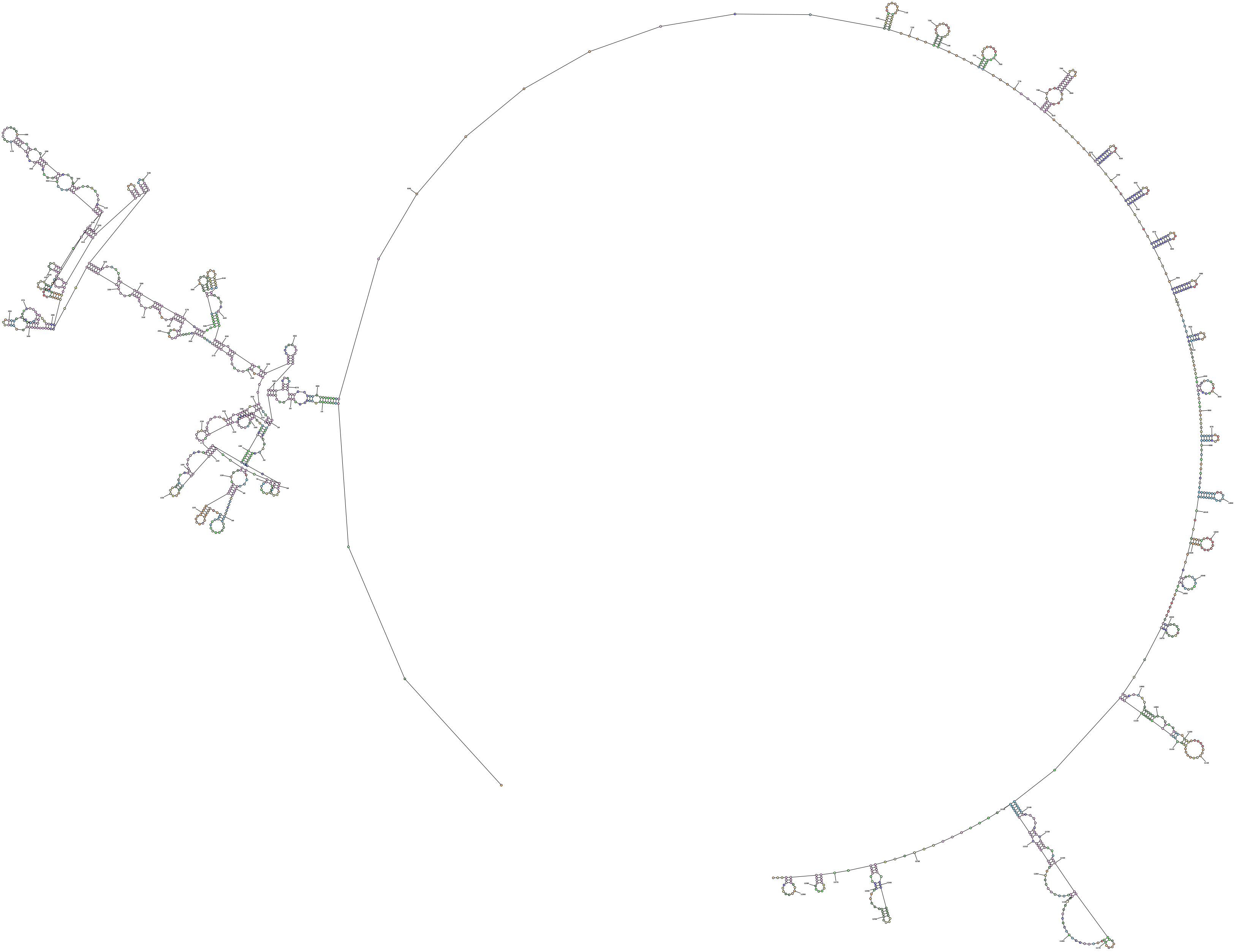

Supplementary Figure S1-10. Secondary structure predictions of the Control Region

Probability >= 99%
99% > Probability >= 95%
95% > Probability >= 90%
90% > Probability >= 80%
80% > Probability >= 70%
70% > Probability >= 60%
60% > Probability >= 50%
50% > Probability

ENERGY = -287.8 CR_T_pinchaque

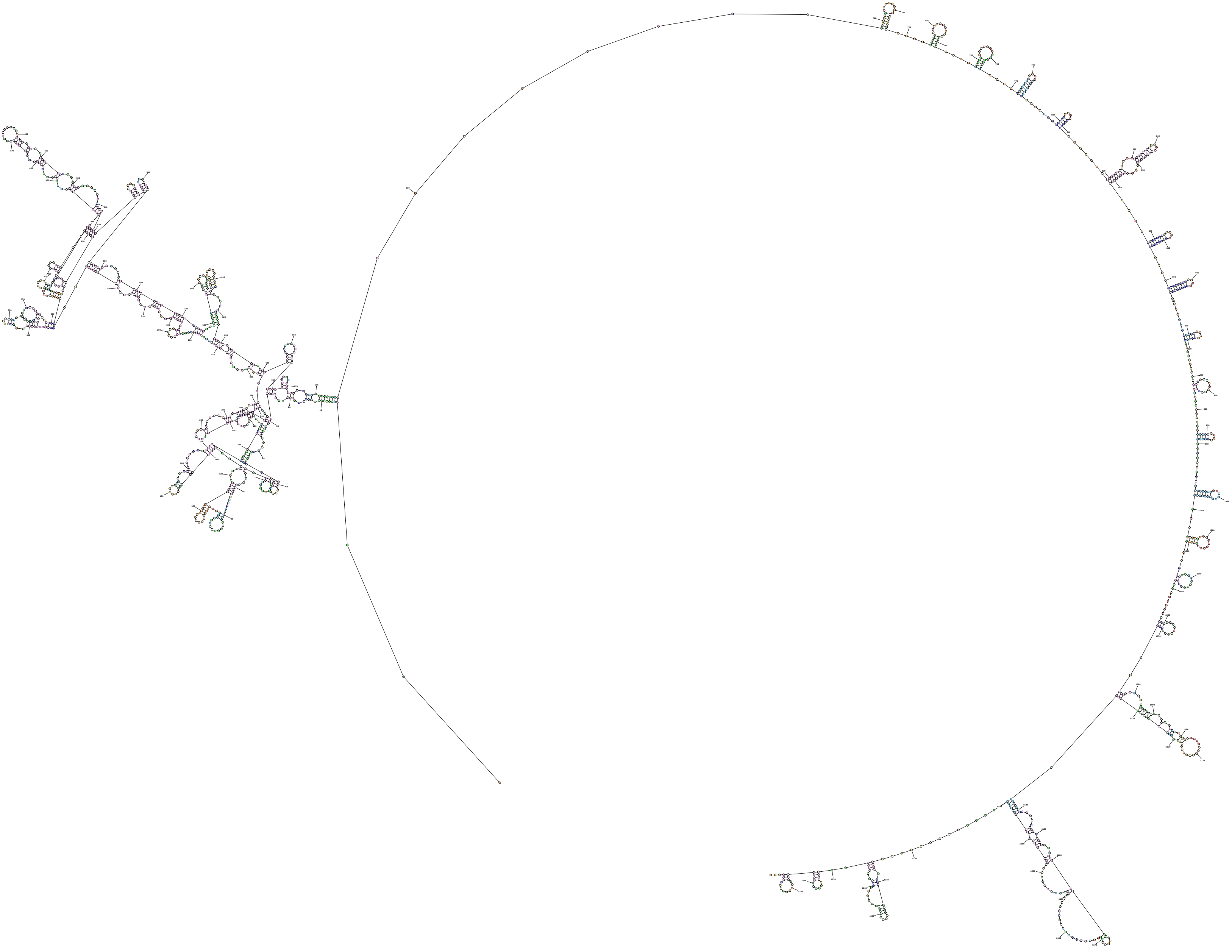

Supplementary Figure S1-11. Secondary structure predictions of the Control Region

Probability >= 99%
99% > Probability >= 95%
95% > Probability >= 90%
90% > Probability >= 80%
80% > Probability >= 70%
70% > Probability >= 60%
60% > Probability >= 50%
50% > Probability

ENERGY = -287.8 CR_T_pinchaque

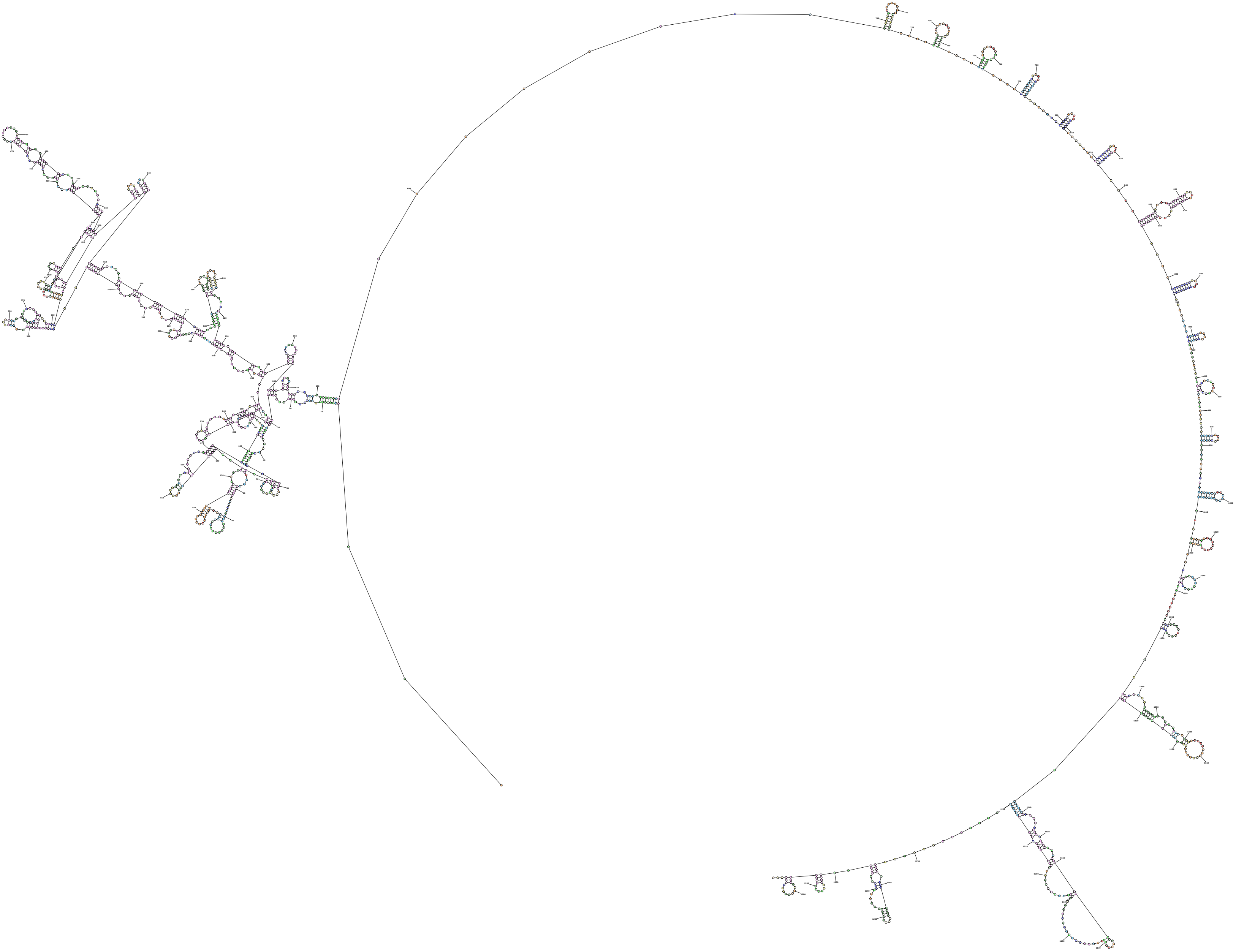

Supplementary Figure S1-12. Secondary structure predictions of the Control Region

Probability >= 99%
99% > Probability >= 95%
95% > Probability >= 90%
90% > Probability >= 80%
80% > Probability >= 70%
70% > Probability >= 60%
60% > Probability >= 50%
50% > Probability

ENERGY = -287.8 CR_T_pinchaque

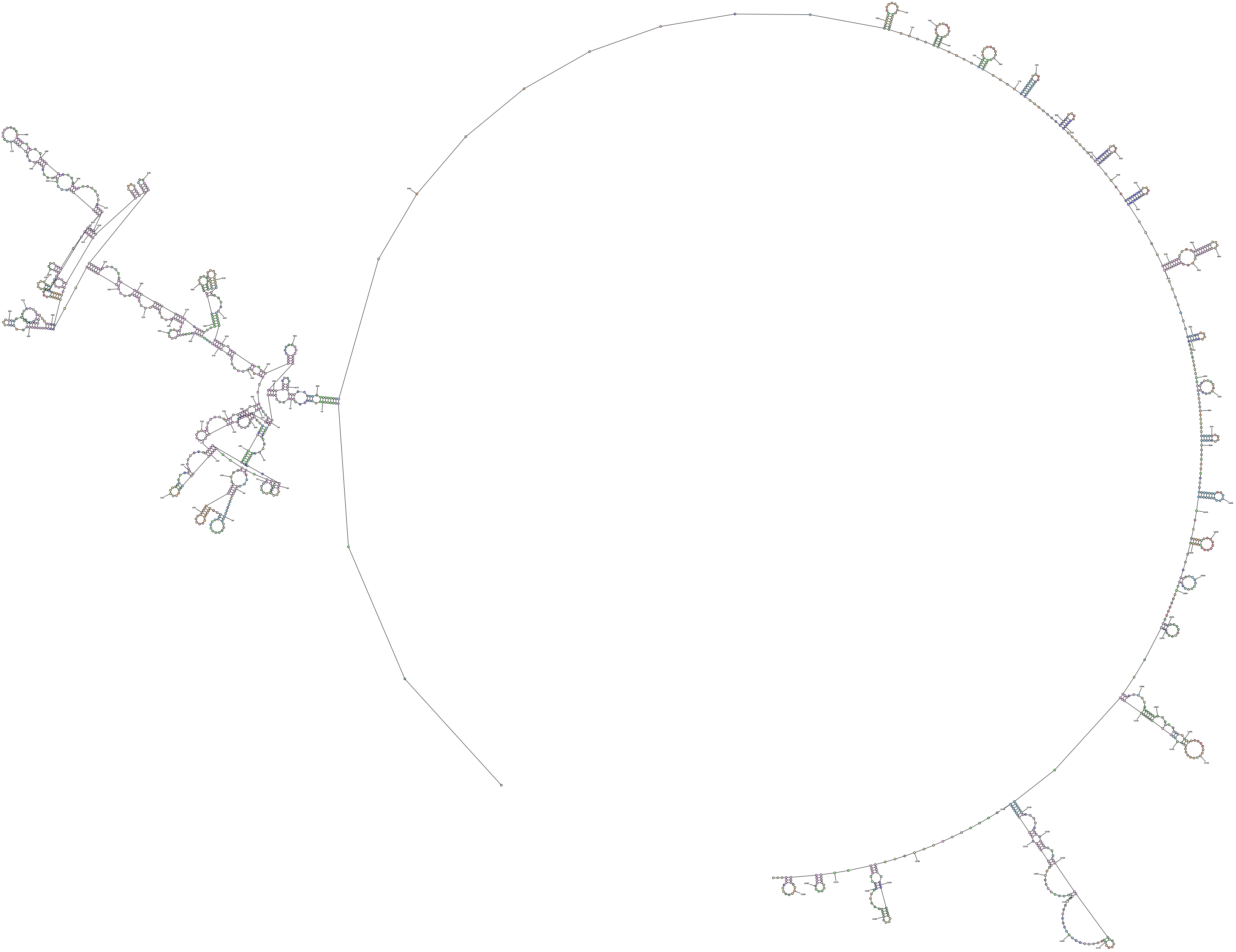

Supplementary Figure S1-13. Secondary structure predictions of the Control Region

Probability >= 99%
99% > Probability >= 95%
95% > Probability >= 90%
90% > Probability >= 80%
80% > Probability >= 70%
70% > Probability >= 60%
60% > Probability >= 50%
50% > Probability

ENERGY = -287.8 CR_T_pinchaque

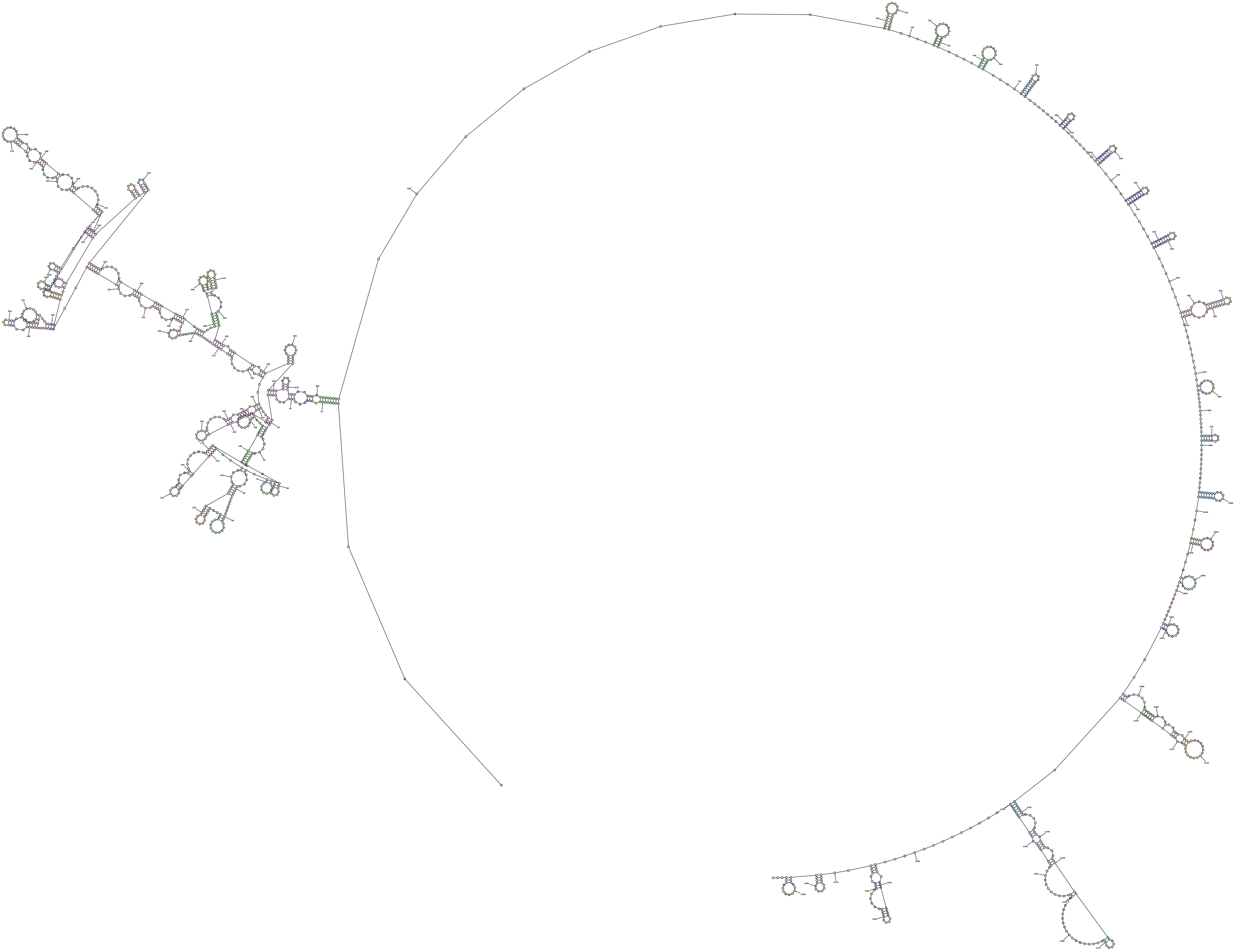

Supplementary Figure S1-14. Secondary structure predictions of the Control Region

Probability >= 99%
99% > Probability >= 95%
95% > Probability >= 90%
90% > Probability >= 80%
80% > Probability >= 70%
70% > Probability >= 60%
60% > Probability >= 50%
50% > Probability

ENERGY = -287.8 CR_T_pinchaque

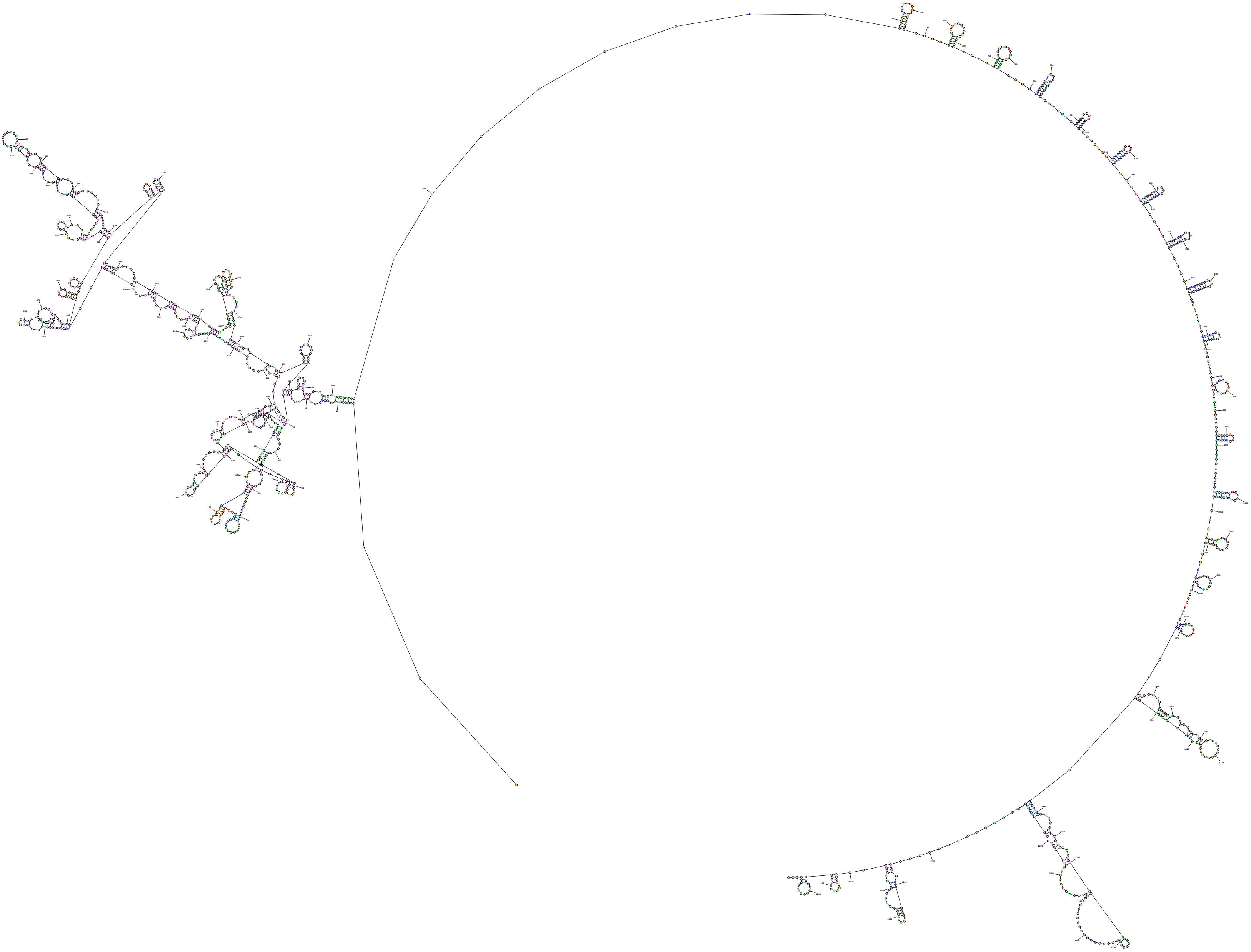

Supplementary Figure S1-15. Secondary structure predictions of the Control Region

Probability >= 99%
99% > Probability >= 95%
95% > Probability >= 90%
90% > Probability >= 80%
80% > Probability >= 70%
70% > Probability >= 60%
60% > Probability >= 50%
50% > Probability

ENERGY = -287.8 CR_T_pinchaque

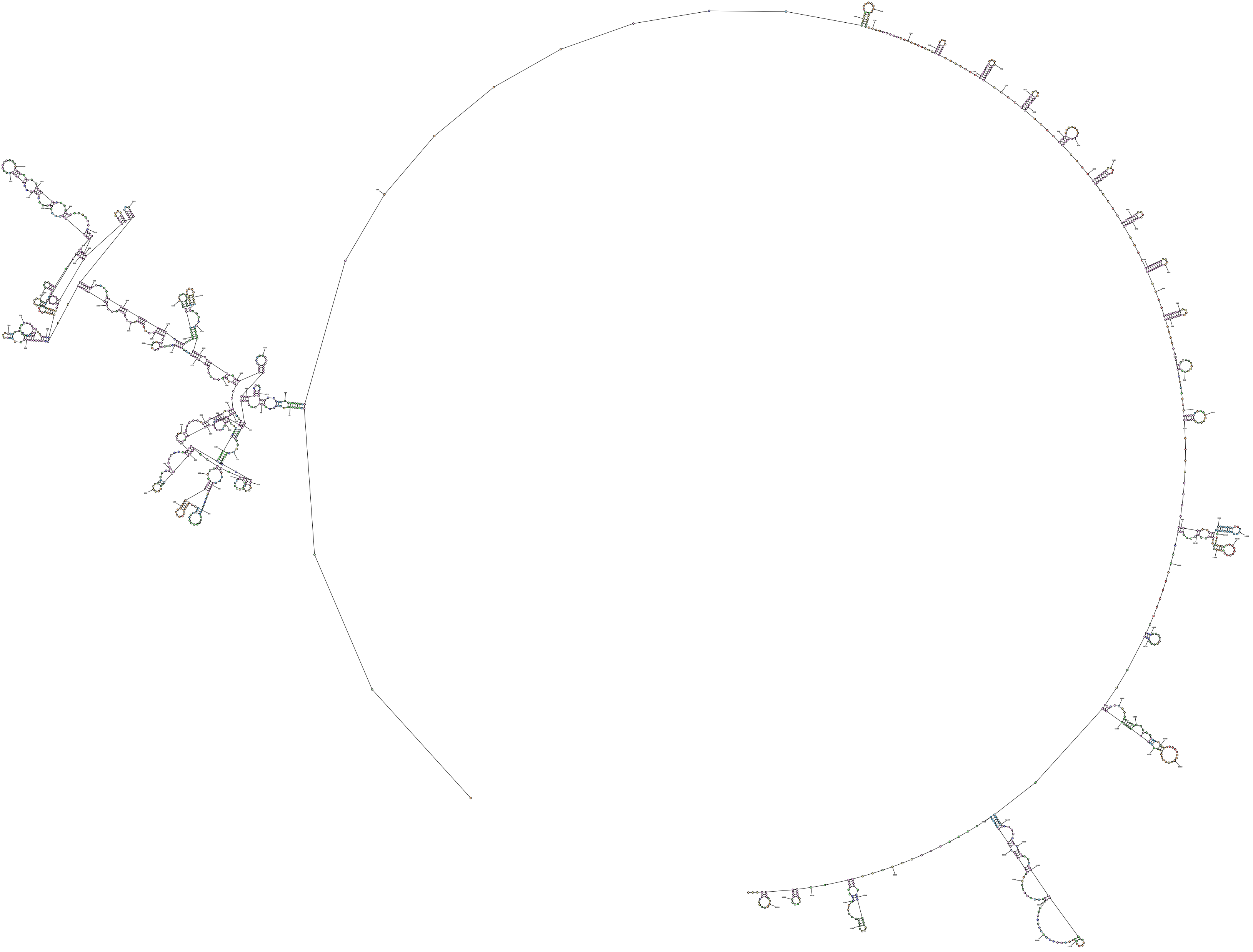

Supplementary Figure S1-16. Secondary structure predictions of the Control Region

Probability >= 99%
99% > Probability >= 95%
95% > Probability >= 90%
90% > Probability >= 80%
80% > Probability >= 70%
70% > Probability >= 60%
60% > Probability >= 50%
50% > Probability

ENERGY = -287.7 CR_T_pinchaque

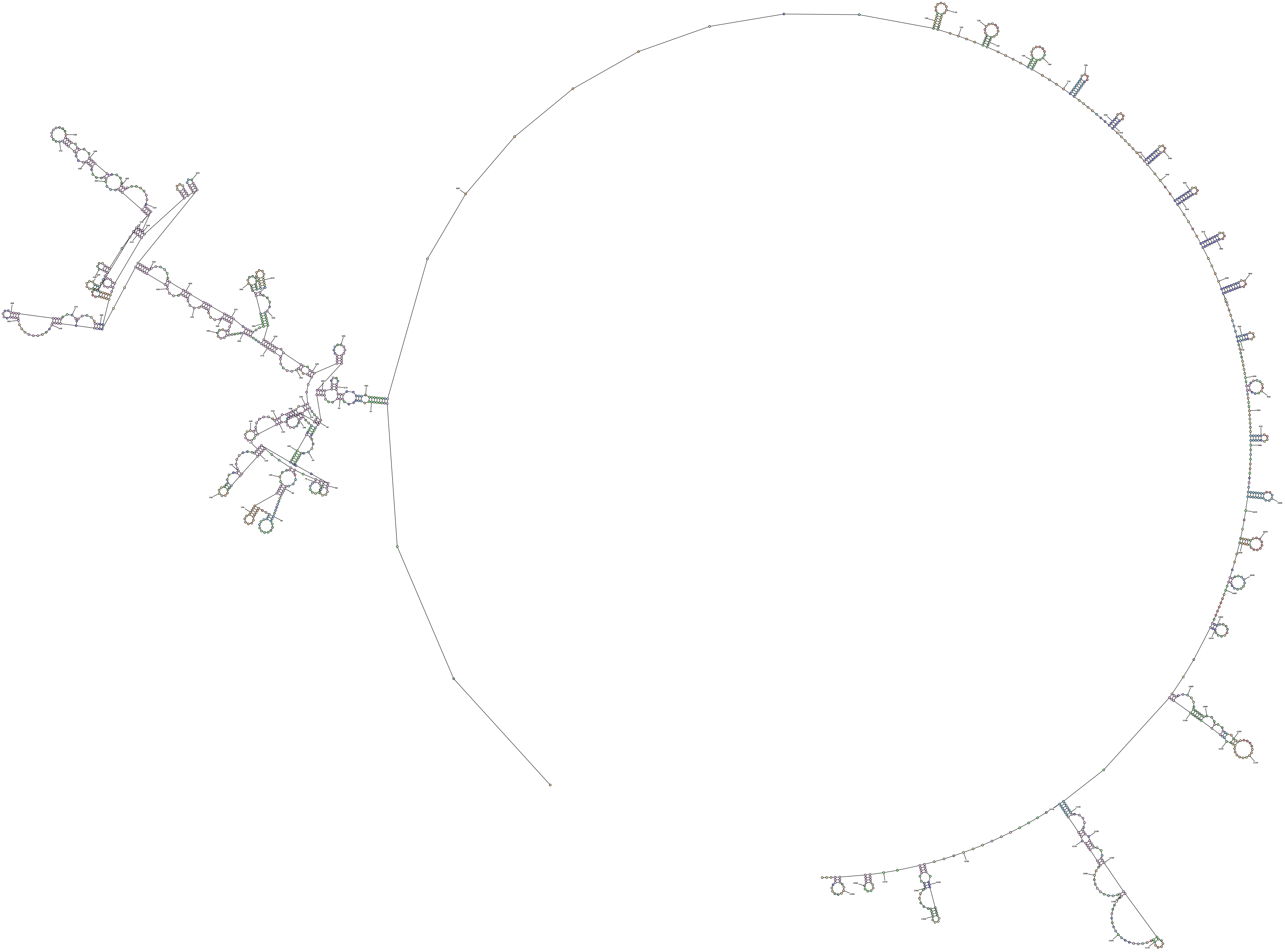

Supplementary Figure S1-17. Secondary structure predictions of the Control Region

Probability >= 99%
99% > Probability >= 95%
95% > Probability >= 90%
90% > Probability >= 80%
80% > Probability >= 70%
70% > Probability >= 60%
60% > Probability >= 50%
50% > Probability

ENERGY = -287.7 CR_T_pinchaque

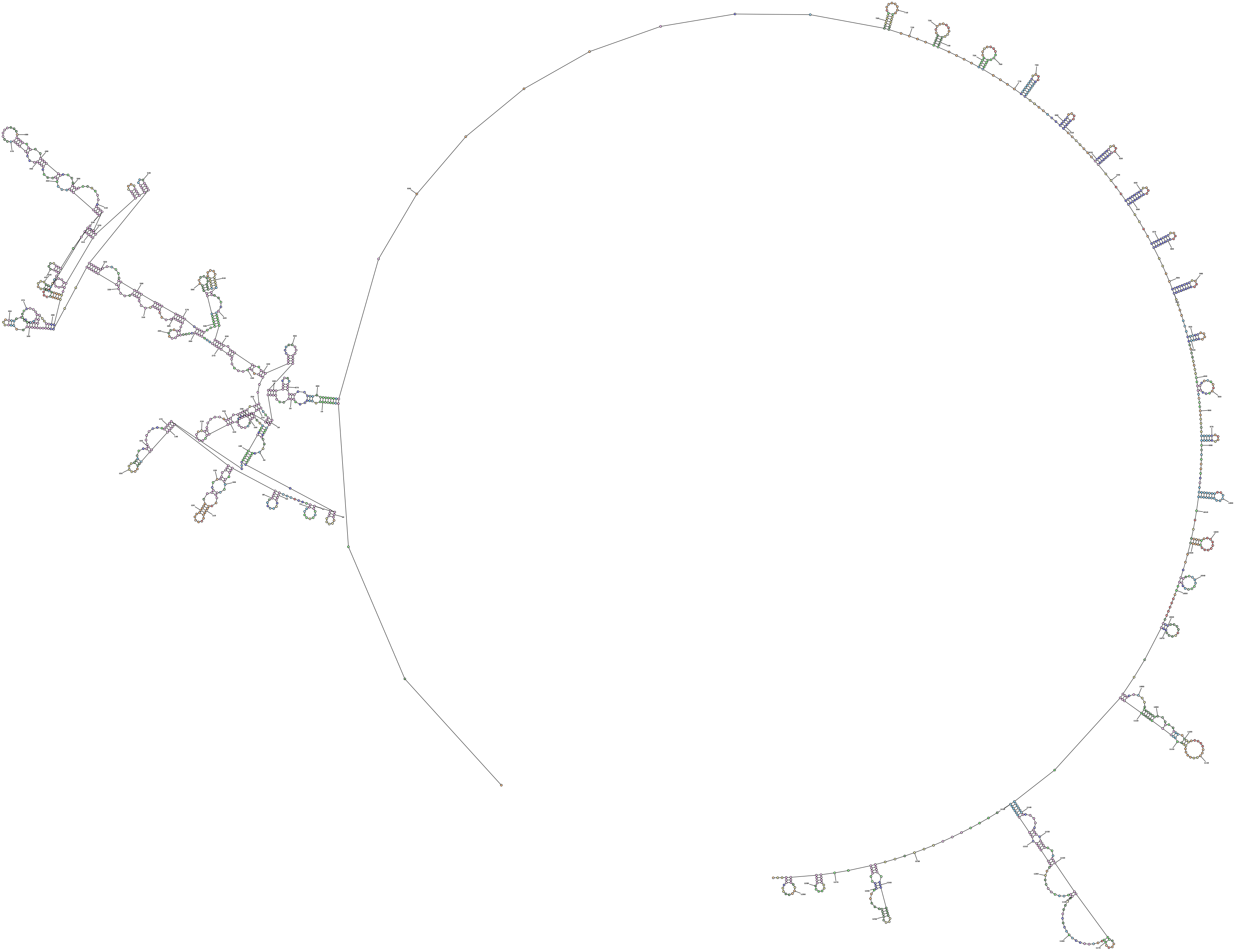

Supplementary Figure S1-18. Secondary structure predictions of the Control Region

Probability >= 99%
99% > Probability >= 95%
95% > Probability >= 90%
90% > Probability >= 80%
80% > Probability >= 70%
70% > Probability >= 60%
60% > Probability >= 50%
50% > Probability

ENERGY = -287.6 CR_T_pinchaque

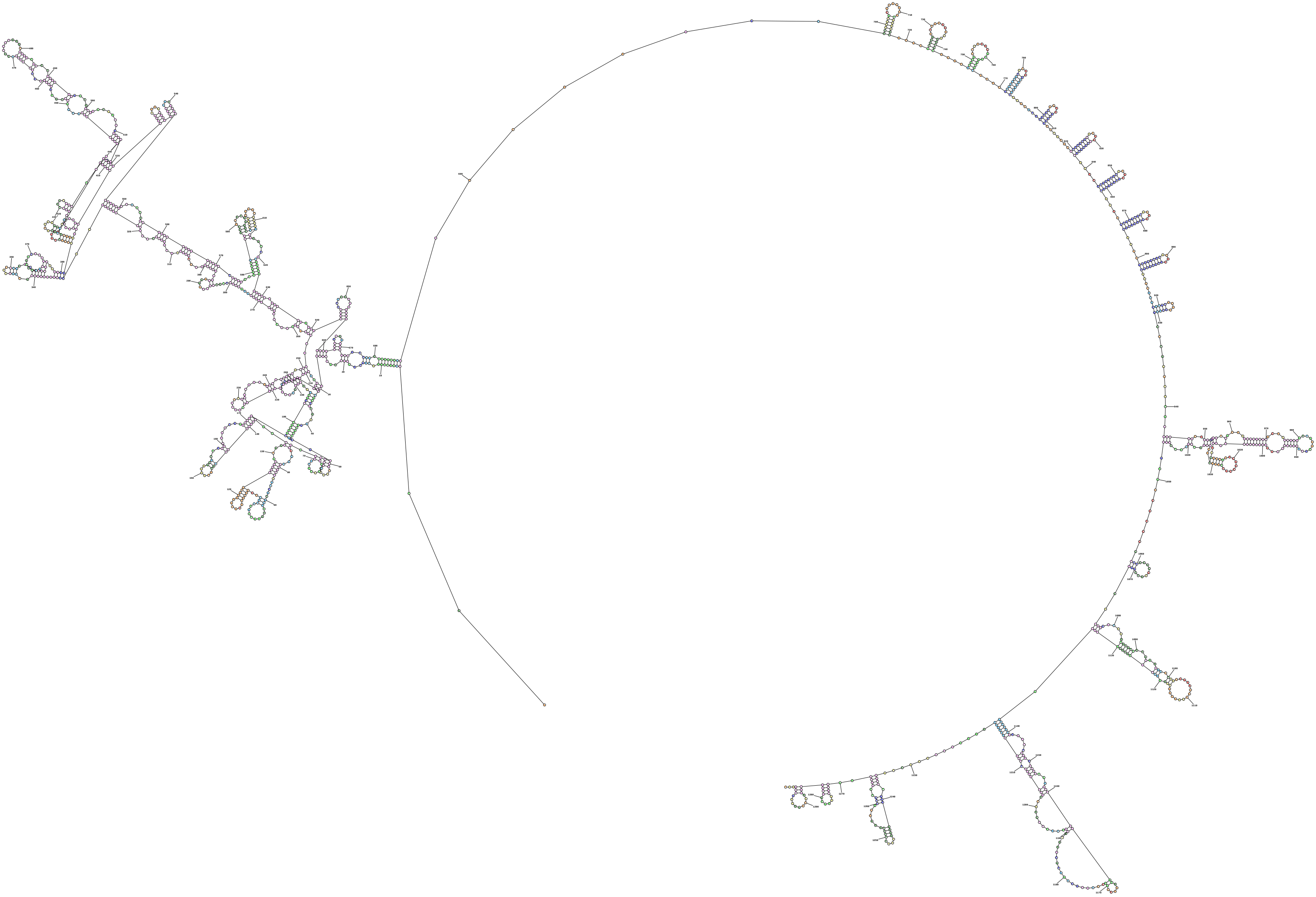

Supplementary Figure S1-19. Secondary structure predictions of the Control Region

Probability >= 99%
99% > Probability >= 95%
95% > Probability >= 90%
90% > Probability >= 80%
80% > Probability >= 70%
70% > Probability >= 60%
60% > Probability >= 50%
50% > Probability

ENERGY = -287.6 CR_T_pinchaque

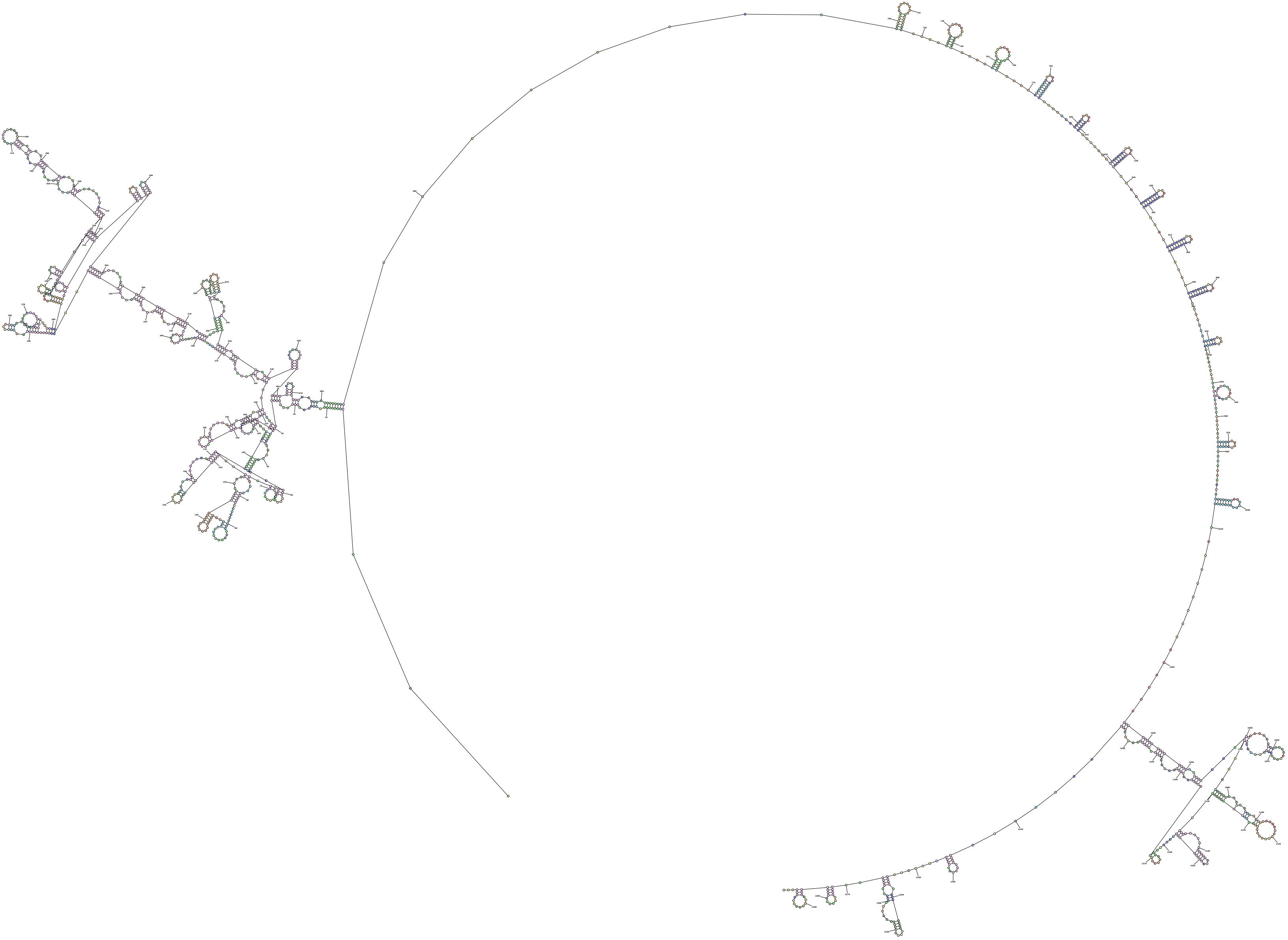

Supplementary Figure S1-20. Secondary structure predictions of the Control Region

Probability >= 99%
99% > Probability >= 95%
95% > Probability >= 90%
90% > Probability >= 80%
80% > Probability >= 70%
70% > Probability >= 60%
60% > Probability >= 50%
50% > Probability

ENERGY = -287.6 CR_T_pinchaque
